# Supplementary figures and images for: Modulation of fungal virulence through CRZ1 regulated F-BAR-dependent actin remodeling and endocytosis in chickpea infecting phytopathogen Ascochyta rabiei
Source: PLoS Genet. 2021 May 17;17(5):e1009137. doi: 10.1371/journal.pgen.1009137 (PMC8158962; doi:10.1371/journal.pgen.1009137)

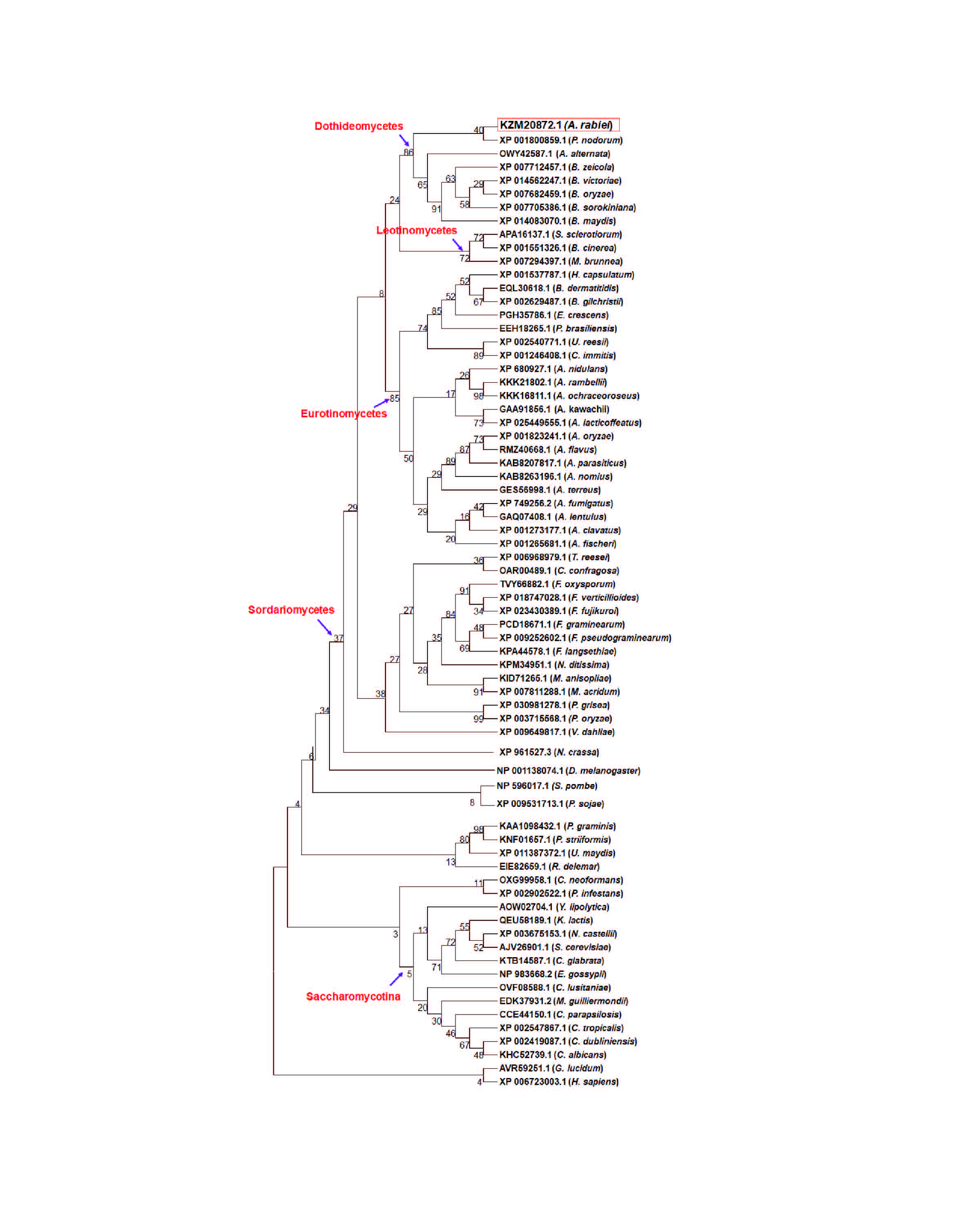

Supplement: S1 Fig — Sequences used: Ascochyta rabiei, Parastagonospora nodorum, Alternaria alternate, Bipolaris zeicola, Bipolaris victoriae, Bipolaris oryzae, Bipolaris sorokiniana, Bipolaris maydis, Sclerotinia sclerotiorum, Botrytis cinerea, Marssonina brunnea, Histoplasma capsulatum, Blastomyces dermatitidis, Blastomyces gilchristii, Emmonsia crescens, Paracoccidioides brasiliensis, Uncinocarpus reesii, Coccidioides immitis, Aspergillus nidulans, Aspergillus rambellii, Aspergillus ochraceoroseus, Aspergillus kawachii, Aspergillus lacticoffeatus, Aspergillus oryzae, Aspergillus flavus, Aspergillus parasiticus, Aspergillus nomius, Aspergillus terreus, Aspergillus fumigatus, Aspergillus lentulus, Aspergillu sclavatus, Aspergillus fischeri, Trichoderma reesei, Cordyceps confragosa, Fusarium oxysporum, Fusarium verticillioides, Fusarium fujikuroi, Fusarium graminearum, Fusarium pseudograminearum, Fusarium langsethiae, Neonectria ditissima, Metarhizium anisopliae, Metarhizium acridum, Pyricularia grisea, Pyricularia oryzae, Verticillium dahlia, Neurospora crassa, Drosophila melanogaster, Schizosaccharomyces pombe, Phytophthora sojae, Phytophthora graminis, Phytophthora striiformis, Ustilago maydis, Rhizopus delemar, Cryptococcus neoformans, Phytophthora infestans, Yarrowia lipolytica, Kluyveromyces lactis, Naumovozyma castellii, Saccharomyces cerevisiae, Candida glabrata, Eremothecium gossypii, Clavispora lusitaniae, Meyerozyma guilliermondii, Candida parapsilosis, Candida tropicalis, Candida dubliniensis, Candida albicans, Ganoderma lucidum, and Homo sapiens. The multiple sequence alignment of protein was performed by PROMALS3D software and the phylogeny was constructed using a software MEGA7.0.21. The bootstrap values, derived from 1000 iterations, validated the obtained phylogeny. (TIF) [file pgen.1009137.s001.tif]

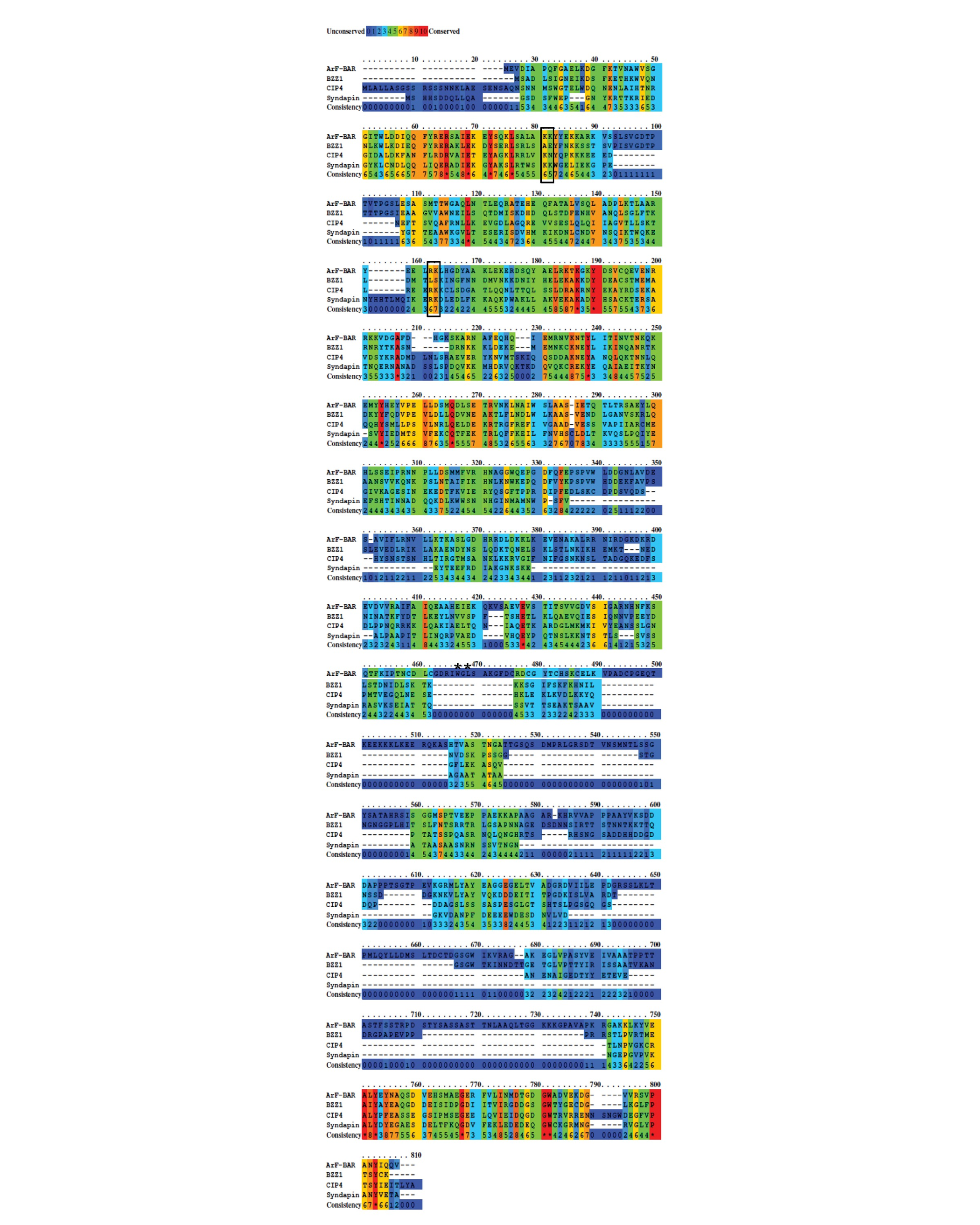

Supplement: S2 Fig — Multiple sequence alignment showing the conservation of ArF-BAR protein with BZZ1p of S. cerevisiae, Cdc42-interacting protein 4 (CIP4) of Drosophila and Syndapin proteins of Drosophila. Colour code for sequence conservation varies from blue (least conserved) to red (highly conserved). The alignment of the protein is determined by Praline software using default parameters. The black box marks the presence of positively charged residues of F-BAR domain. Asterisk (*) represents the residues in C1 domain of protien Kinase C1 (PKC1), required for interaction with DAG. (TIF) [file pgen.1009137.s002.tif]

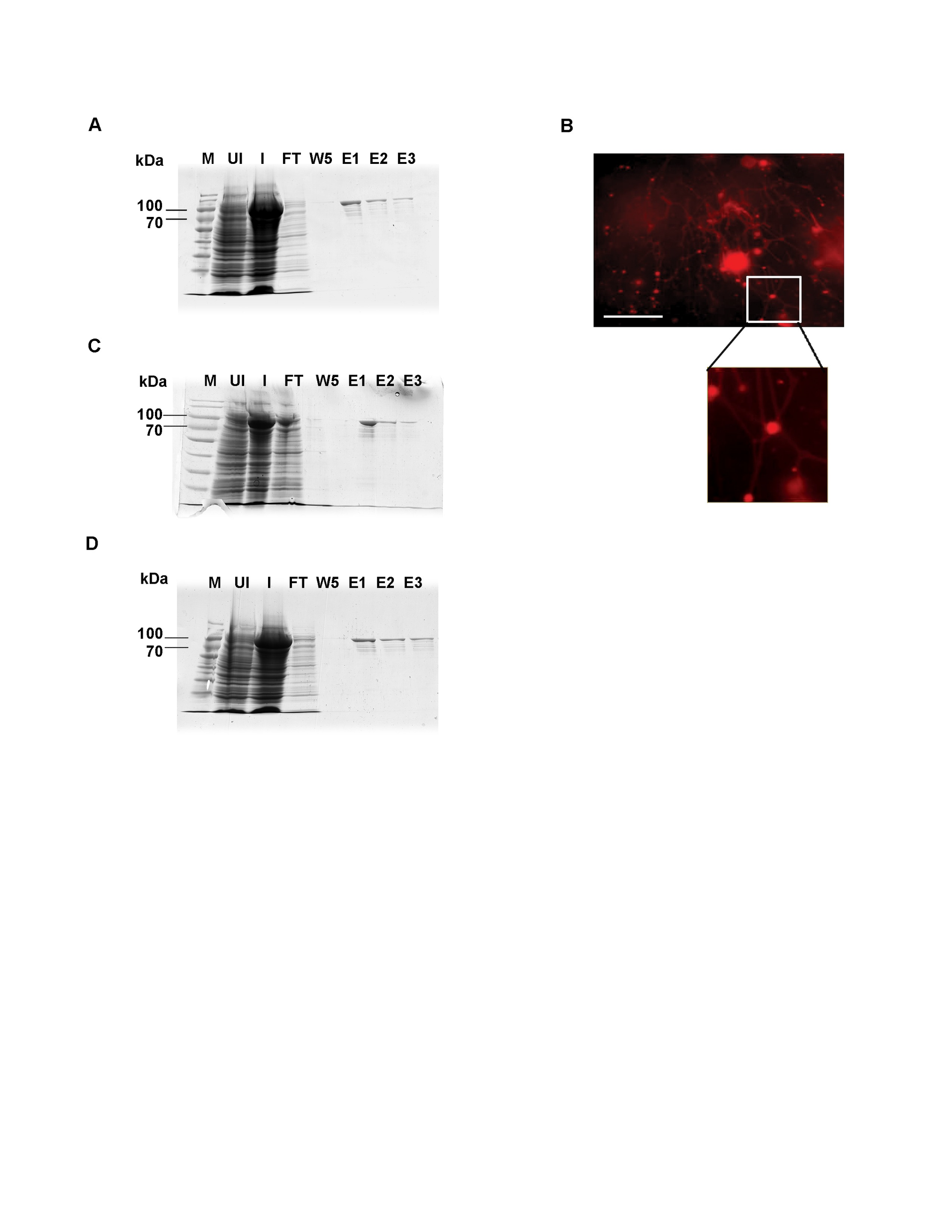

Supplement: S3 Fig — (A) His purification of bacterially expressed ArF-BAR protein. Analysis of the purification of recombinant ArF-BAR as shown by SDS-PAGE. (B) Intense tubular network in synthetic liposomes is formed after 30 min incubation with purified recombinant ArF-BAR protein. Inset showing the enlarged view of dense tubular network originating from a liposome. (C- D) His purification of ArF-BARmut1 and ArF-BARmut2. The protein was visualised by Coomassie Brilliant Blue staining. (UI- crude extract of un-induced samples after centrifugation; I- crude extract of induced samples after centrifugation; FT- flow-through fraction of the Nickel chelating resin column; W5- 5th wash fraction of the Nickel chelating resin column; E1, E2 and E3- eluate fractions of the Nickel chelating resin column showing the purified ArF-BAR protein). Protein standards are shown (M) and their masses are indicated in kDa. (TIF) [file pgen.1009137.s003.tif]

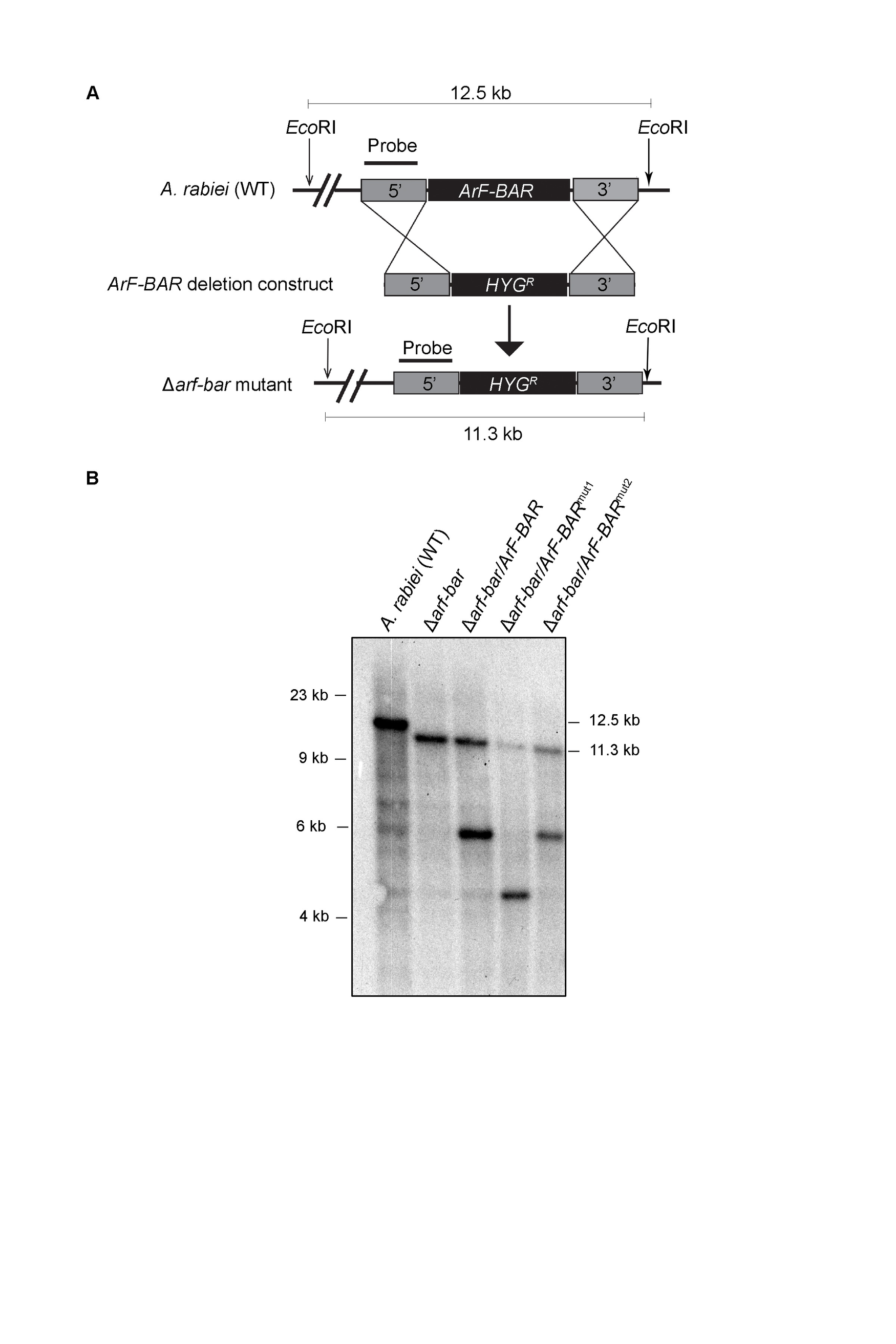

Supplement: S4 Fig — (A) The schematic representation of A. rabiei knockout mutant generation by the homologous recombination approach to obtain targeted ArF-BAR gene deletion mutants (Δarf-bar). The bar represents the genomic region used to generate probe for Southern confirmation. (B) The representative Southern blot confirming successful ArF-BAR gene deletion (Δarf-bar), with a single integration of hph at replacement site. Along with the confirmation of ArF-BAR complementation in Δarf-bar, followed successful generation of Δarf-bar/ArF-BARmut1and Δarf-bar/ArF-BARmut2 complementation. (TIF) [file pgen.1009137.s004.tif]

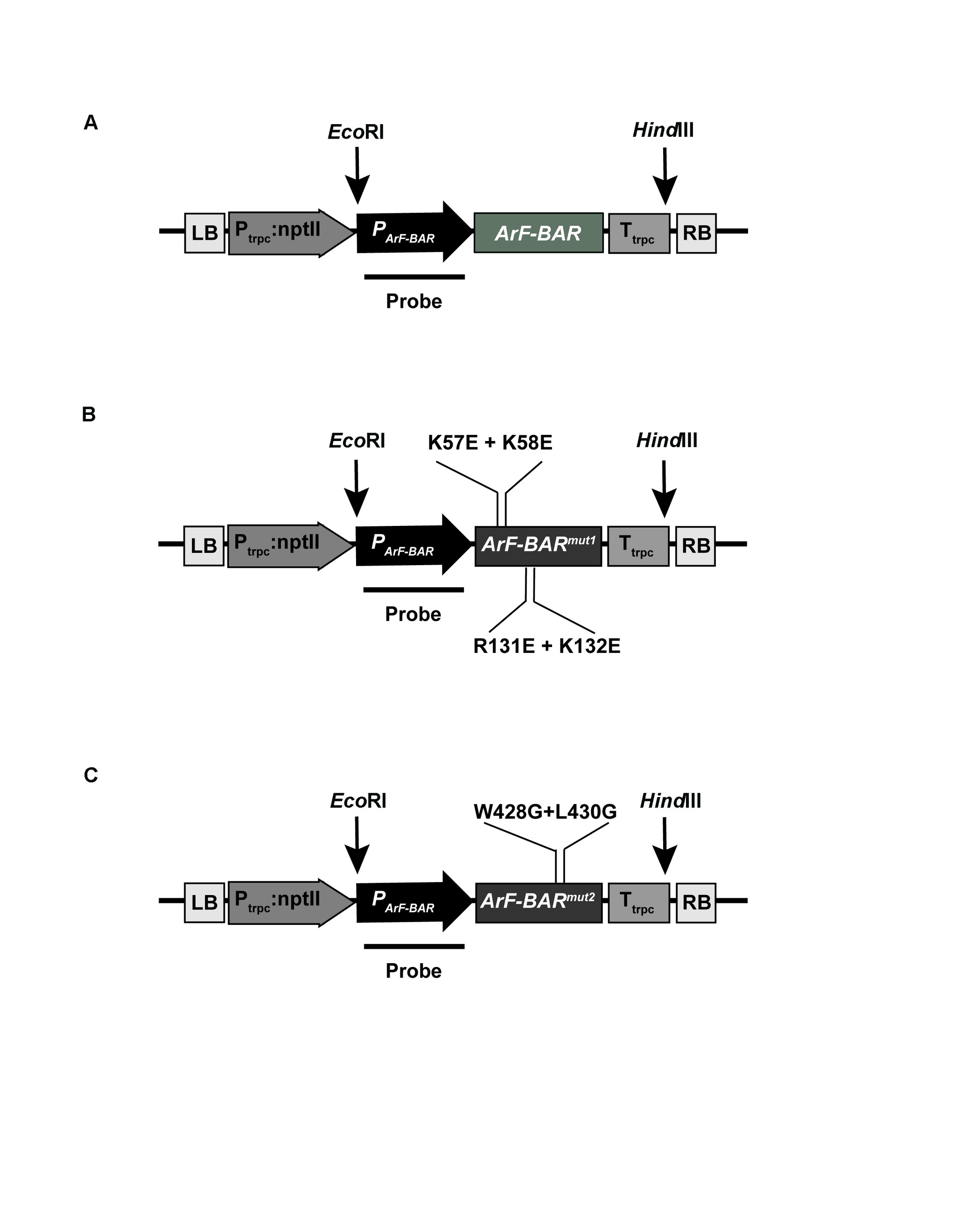

Supplement: S5 Fig — (A, B, C) Constructs used to generate different Δarf-bar mutant complemented strains (Δarf-bar/ArF-BAR, Δarf-bar/ArF-BARmut1, and Δarf-bar/ArF-BARmut2) under the control of native promoter of ArF-BAR gene. (TIF) [file pgen.1009137.s005.tif]

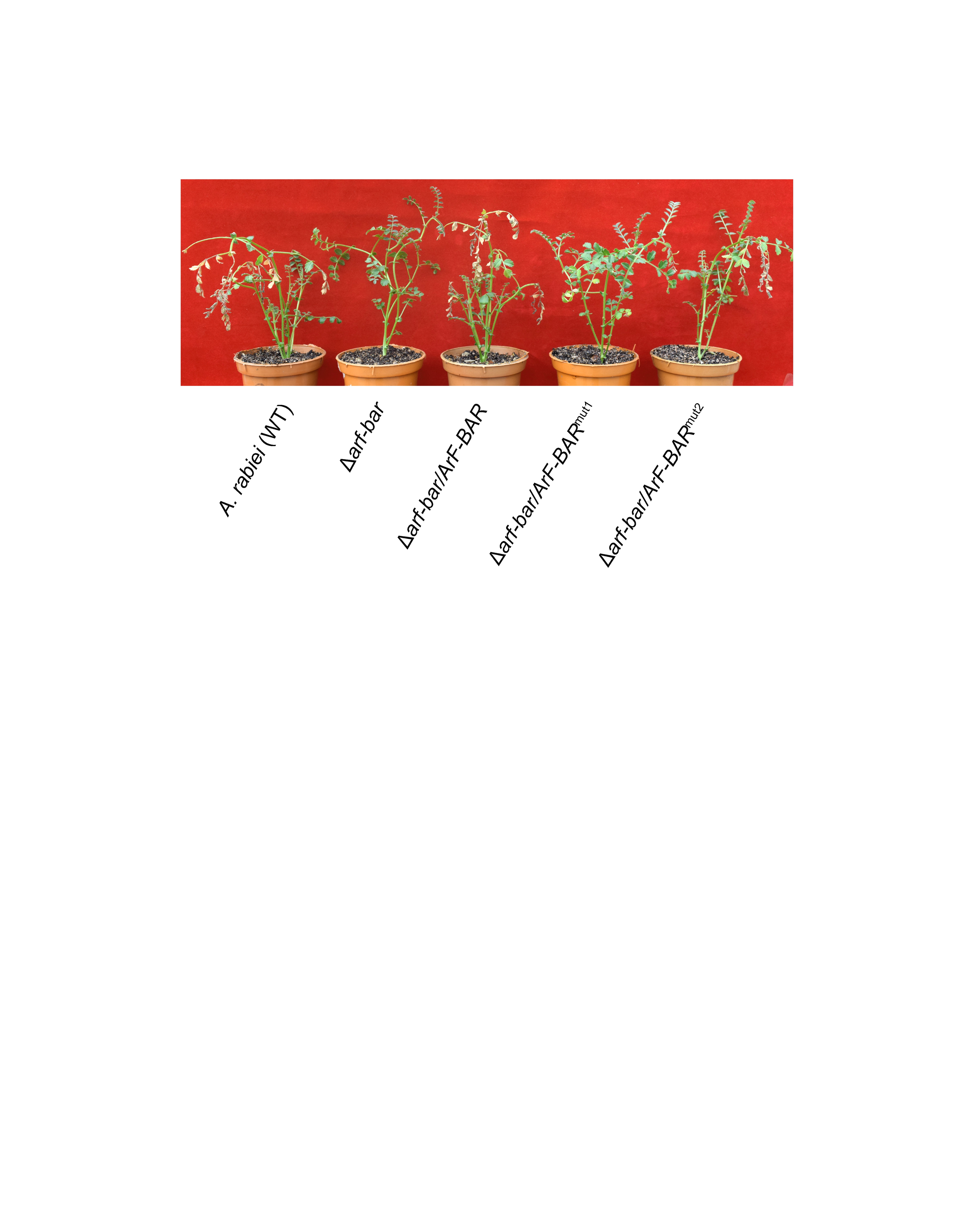

Supplement: S6 Fig — The susceptible plants inoculated with conidia of A. rabiei (WT), Δarf-bar/ArF-BAR, and Δarf-bar/ArF-BARmut2, showed severe disease symptoms with the increasing duration after inoculation. Δarf-bar and Δarf-bar/ArF-BARmut1 challenged plants showed no or very mild symptoms after 10 dpi. (TIF) [file pgen.1009137.s006.tif]

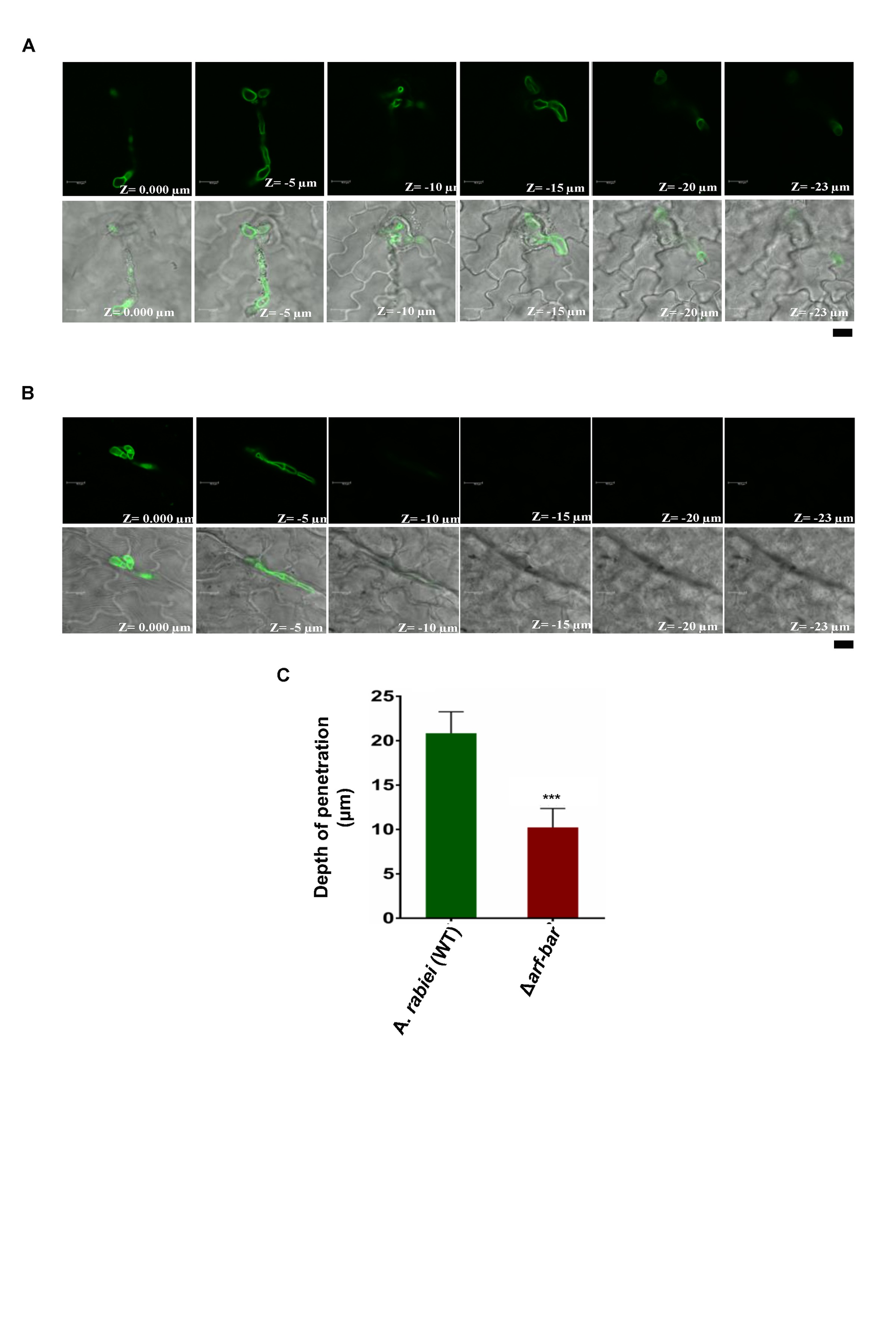

Supplement: S7 Fig — (A, B) Confocal images showing the depth of penetration 48 hpi by A. rabiei (WT) and Δarf-bar strains, respectively, in AB susceptible chickpea leaves. Fungal hyphae were stained with WGA-488 for visualization, prior to microscopy. The Z-stacked images were acquired till 23 μm depth, starting from the surface of the leaves. The image is the representation of maximum projections of all the Z-stacks. Scale bar = 5 μm. (C) The bar graph, representing mean and SD, shows the difference in ability to penetrate within the host by A. rabiei (WT) and Δarf-bar. The results were analysed using Student’s t-test one tailed compared to its control (*p = 0.0079). (TIF) [file pgen.1009137.s007.tif]

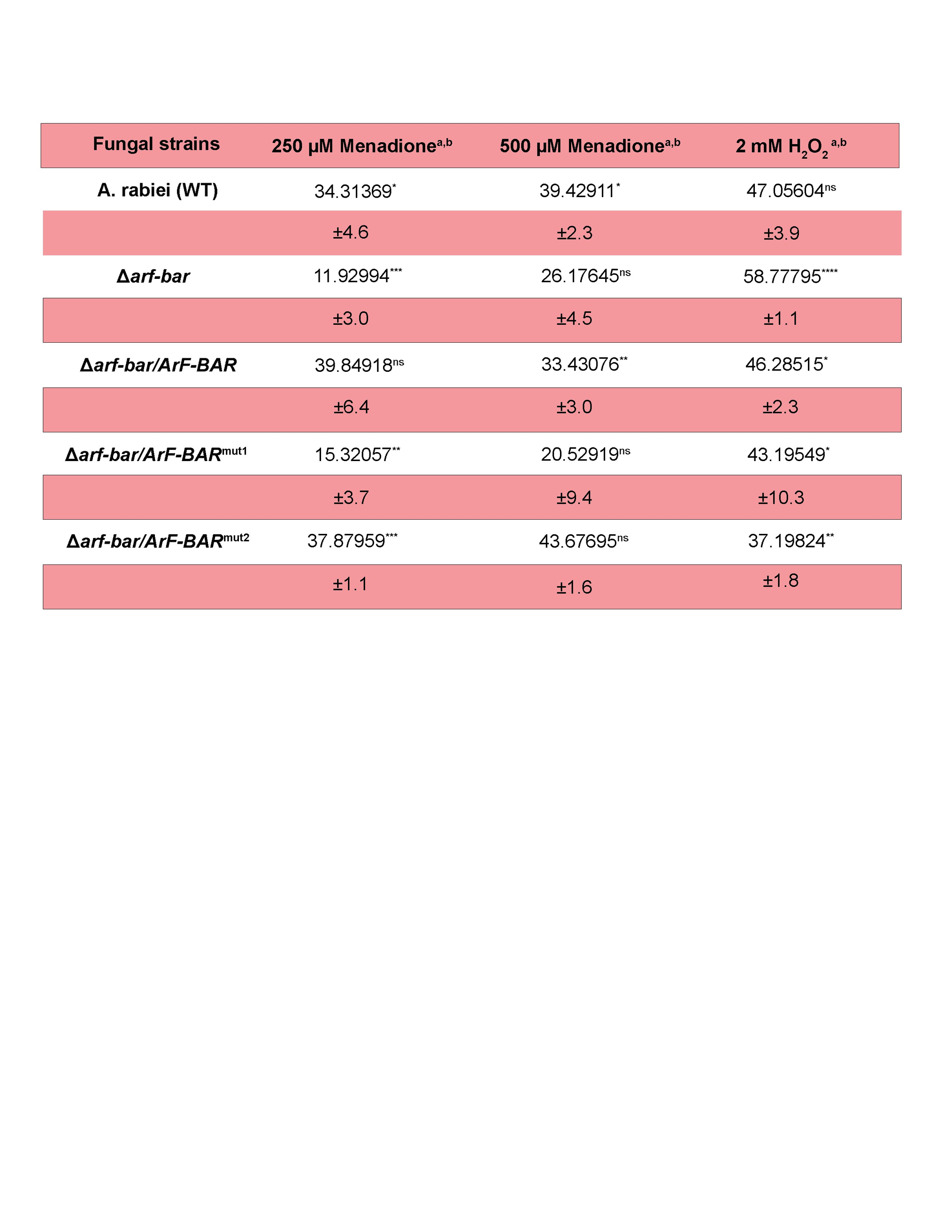

Supplement: S8 Fig — The A. rabiei (WT), Δarf-bar mutant and mutant complemented strains, observed 10 days after incubation at 22°C. For oxidative stress, the PDA was supplemented with 250 μM and 500 μM menadione and 2 mM H2O2. Strains of Δarf-bar and Δarf-bar/ArF-BARmut1 exhibited more growth inhibition towards oxidative stress condition as compared to WT. (TIF) [file pgen.1009137.s008.tif]

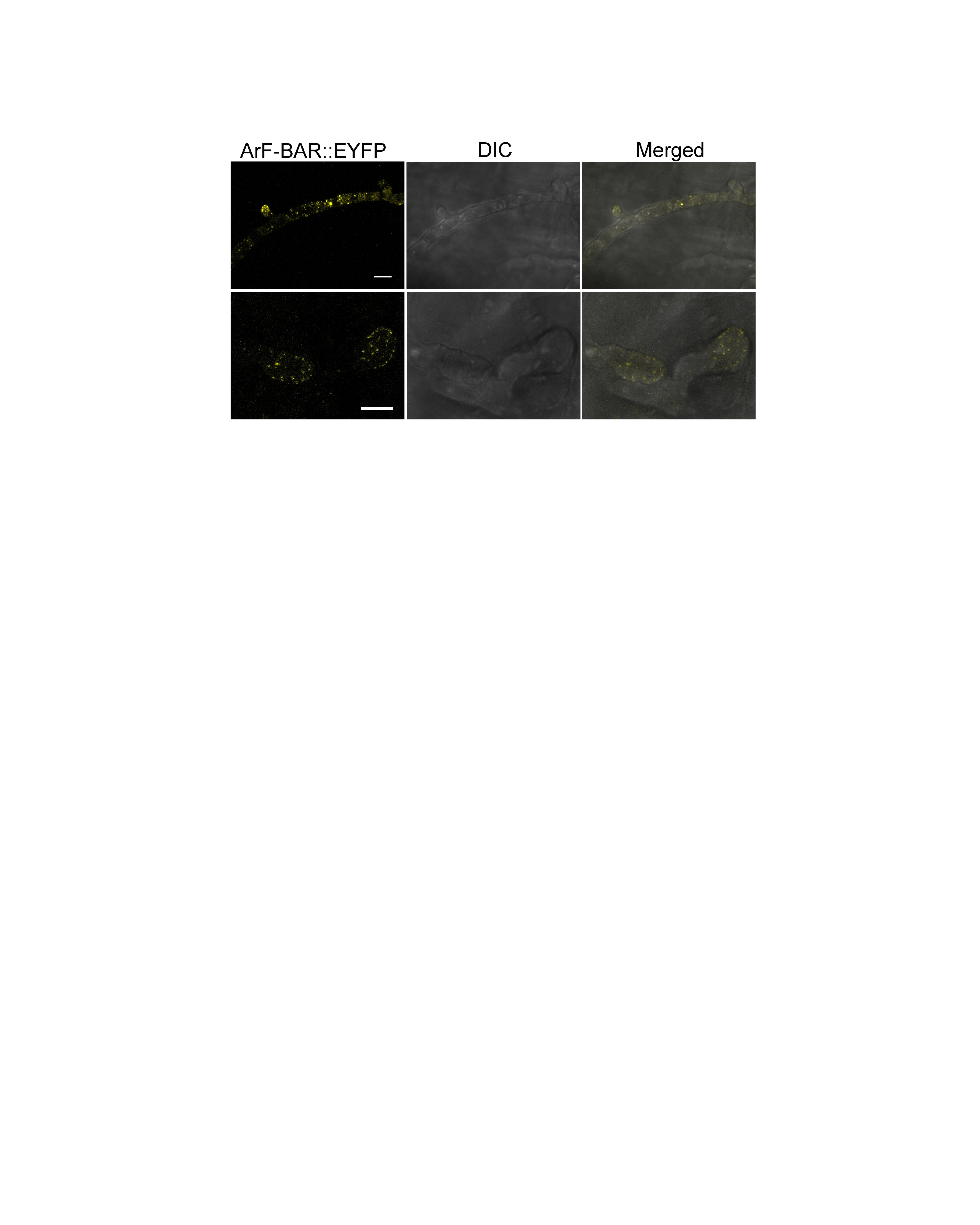

Supplement: S9 Fig — Confocal micrographs showing the punctate distribution of ArF-BAR::EYFP during host infection. The representative image is the maximum intensity projection of all the Z-stack images with 0.5 μm step size, acquired after 48 hpi of susceptible chickpea with fungal conidia expressing chimeric ArF-BAR::EYFP. Scale bar = 5 μm (n = 12). (TIF) [file pgen.1009137.s009.tif]

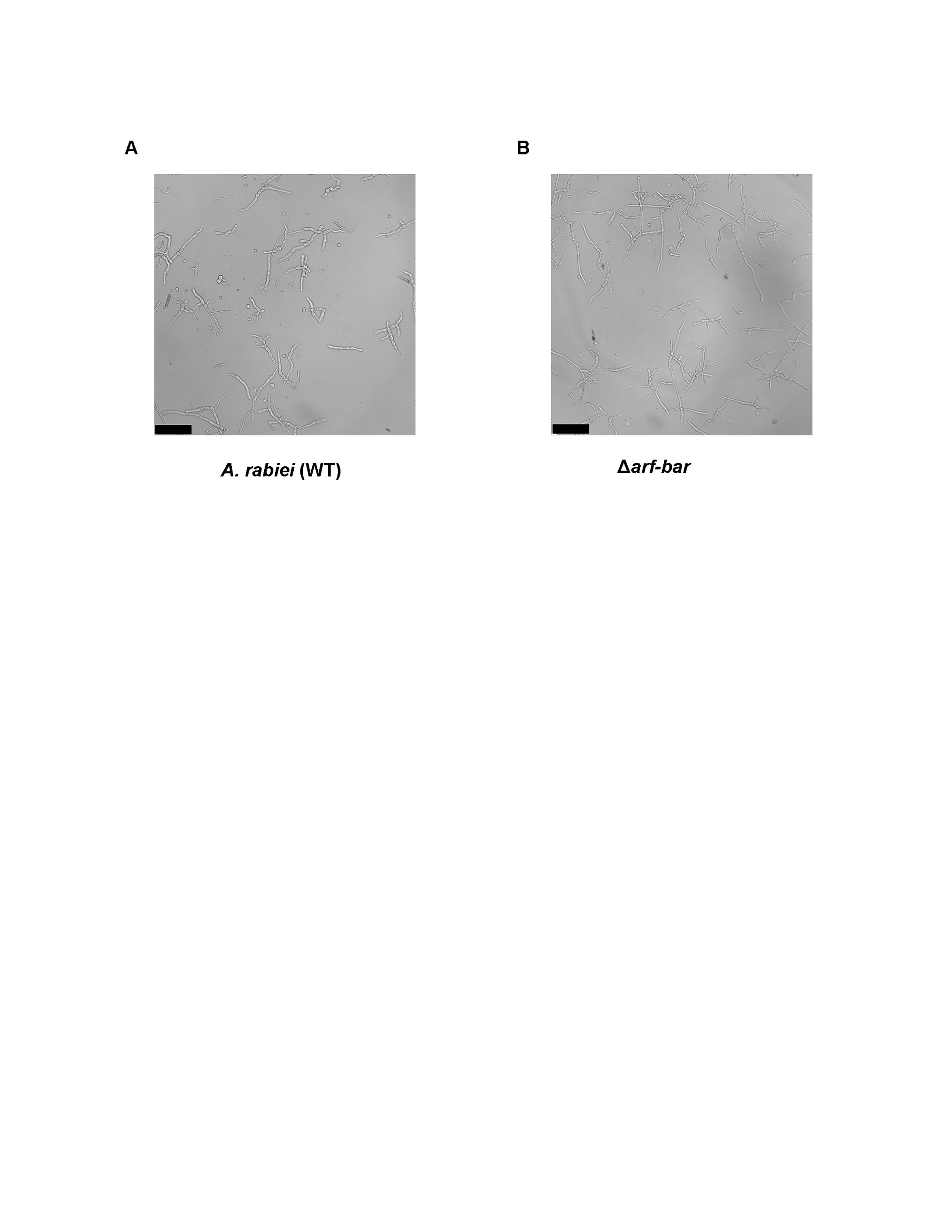

Supplement: S10 Fig — Difference in growth pattern on hydrophobic surfaces was slightly observed and microscopic photographs (20X) were taken after 12 h of conidial spread. Scale bar = 50μm. (TIF) [file pgen.1009137.s010.tif]

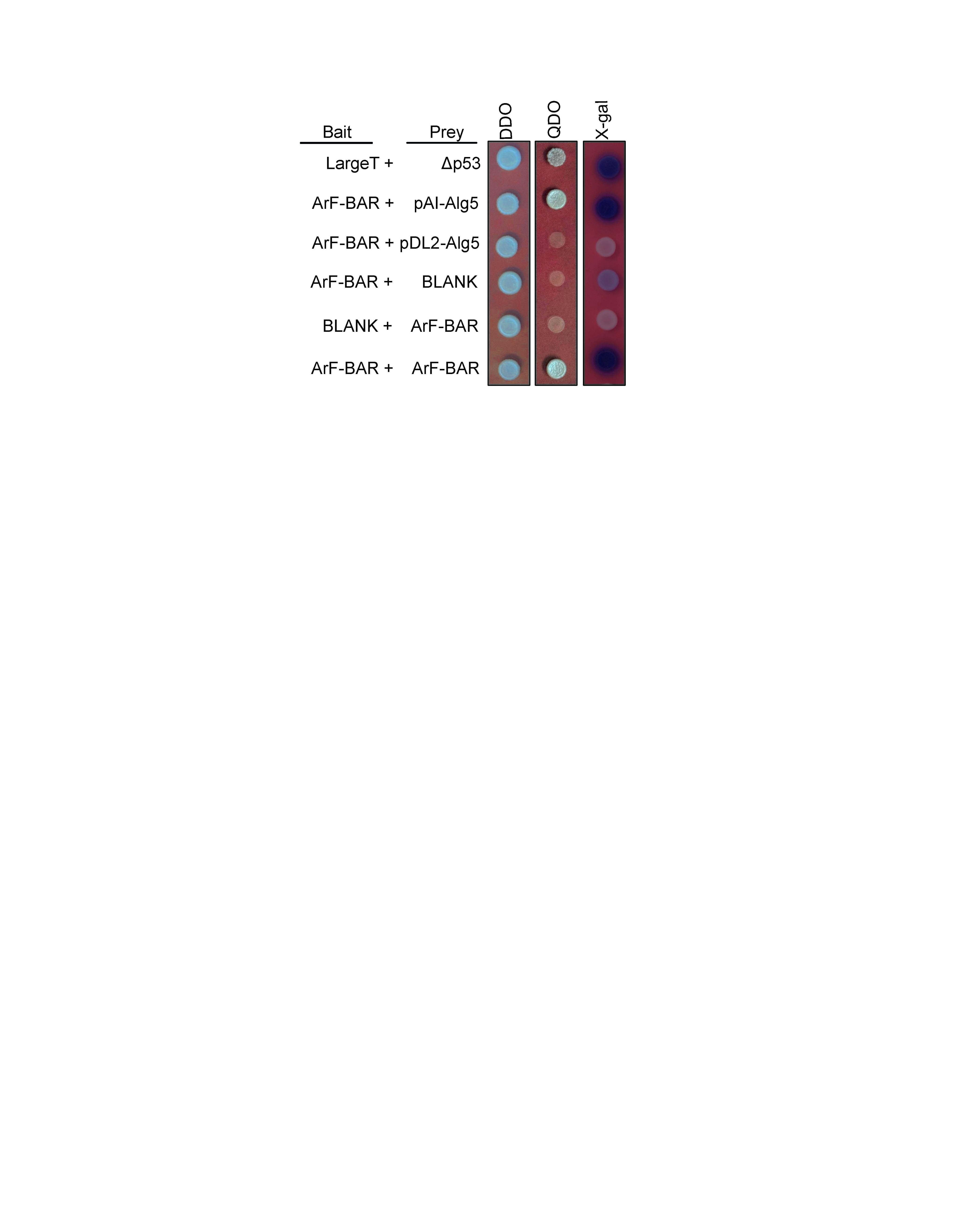

Supplement: S11 Fig — Split-Ubiquitin based Y2H system was used to determine the homodimerization between ArF-BAR proteins. Plates were photographed after 48 h of yeast growth. Strong positive interaction between two ArF-BAR proteins was reflected with the growth on QDO (SD/-L/-W/-A/-H) media and X-gal overlay assay to check the activation LacZ gene. (TIF) [file pgen.1009137.s011.tif]

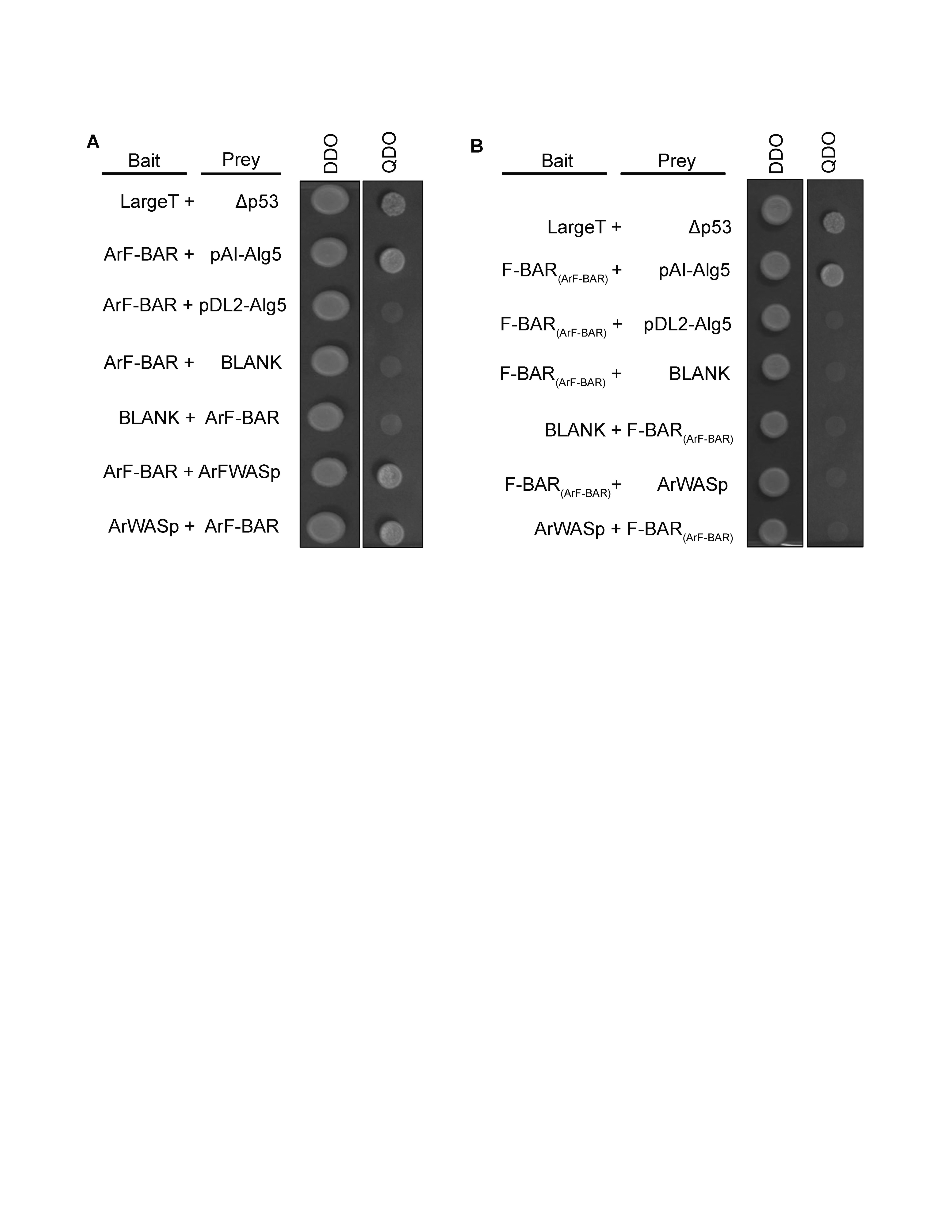

Supplement: S12 Fig — (A) The yeast two-hybrid result showing the positive interaction of ArF-BAR protein with ArWASp. (B) F-BAR domain (amino acids 1–325) of ArF-BAR protein [F-BAR(ArF-BAR)] failed to interact with ArWASp in Y2H system. Plates were photographed 48 h after yeast spotting. The interaction was confirmed by three independent replicates. (TIF) [file pgen.1009137.s012.tif]

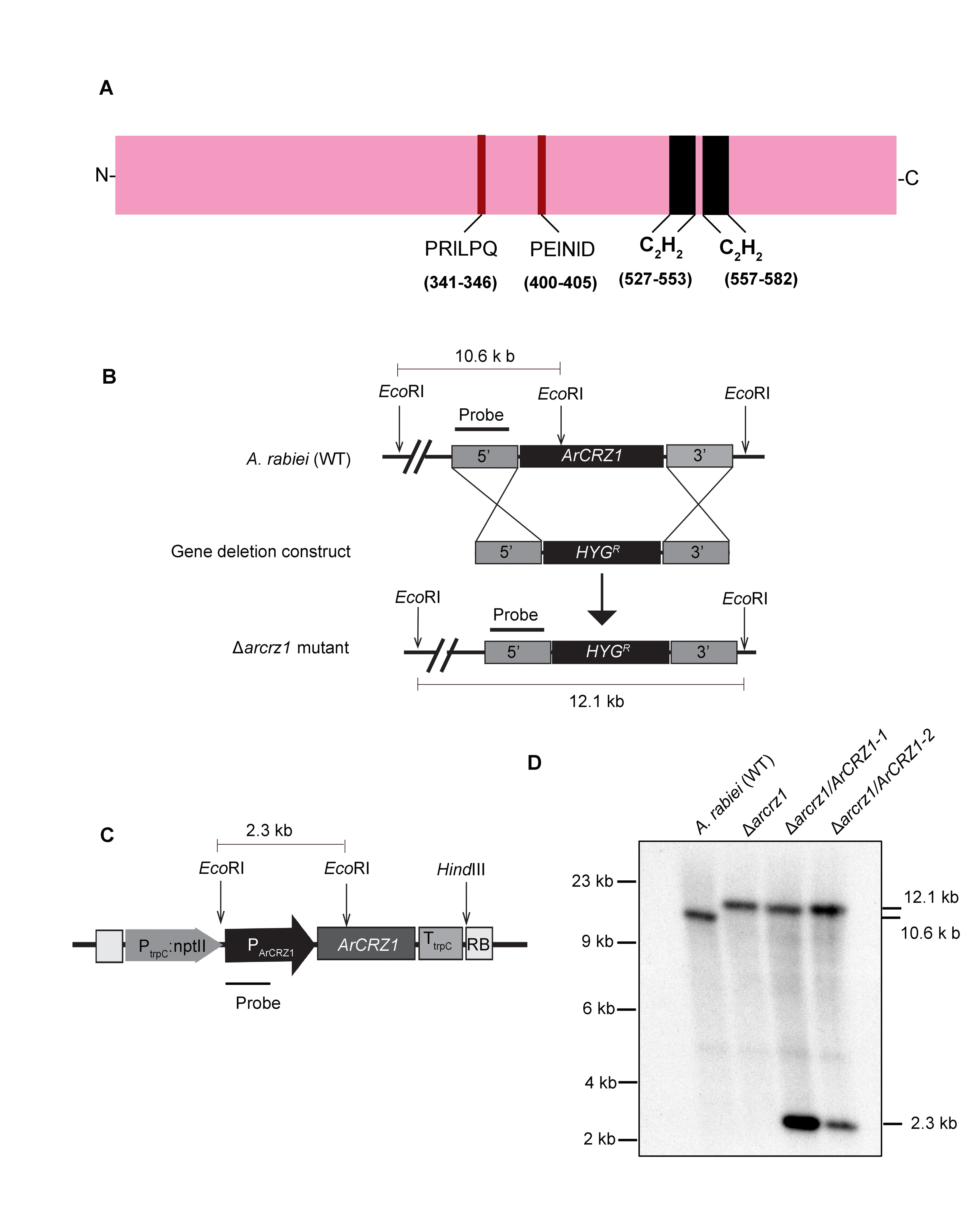

Supplement: S13 Fig — (A) Schematic representation of domain organisation of ArCRZ1 protein. (B) The schematic map showing the homologous recombination based knockout approach used for targeted ArCRZ1 gene deletion mutant (Δcrz1) strain generation. (C) Schematic representation of Δarcrz1/ArCRZ1 complementation construct under the native promoter of ArCRZ1. The genomic region used to generate probe for confirmation by Southern hybridization is being highlighted. (D) The Southern blot result confirmed successful ArCRZ1 gene deletion (Δarcrz1), with single integration of hph at replacement site along with the complementation confirmation of ArCRZ1 in Δarcrz1 mutant. (TIF) [file pgen.1009137.s013.tif]
