# Supplementary material for: Modulation of fungal virulence through CRZ1 regulated F-BAR-dependent actin remodeling and endocytosis in chickpea infecting phytopathogen Ascochyta rabiei
Source: PLoS Genet. 2021 May 17;17(5):e1009137. doi: 10.1371/journal.pgen.1009137 (PMC8158962; doi:10.1371/journal.pgen.1009137)

**S1 Table:** YEASTRACT result for the putative transcription factors bindings on the *cis*-acting regulatory sequences of ArF-BAR. The position of each core-motif is shown in a box below table.

| **S.**  **No.** | **Transcription Factor** | **Core binding site** | **Function** |
| --- | --- | --- | --- |
| 1 | **Adr1p** | TTGGRG | Essential for fatty acid degradation and peroxisome proliferation. |
| 2 | **Crz1p** | GAGCCC | TF that activates transcription of genes involved in stress response. |
| 3 | **Cup2p** | HTHNNGCTGD | Copper-binding TF that activates transcription of the metallothionein genes CUP 1-1 and CUP 1-2 in response to elevated copper. |
| 4 | **Aft2p** | TGCACCC | Iron-regulated transcriptional activator |
| 5 | **Hac1p** | CCAGC | bZIP transcription factor that regulates the unfolded protein response |
| 6 | **Mot3p** | CAGGYA | Involved in repression of a subset of hypoxic genes by Rox1p, repression of several DAN/TIR genes during aerobic growth |
| 7 | **Stb5p** | CGGNS | Activates genes involved in NADPH production |


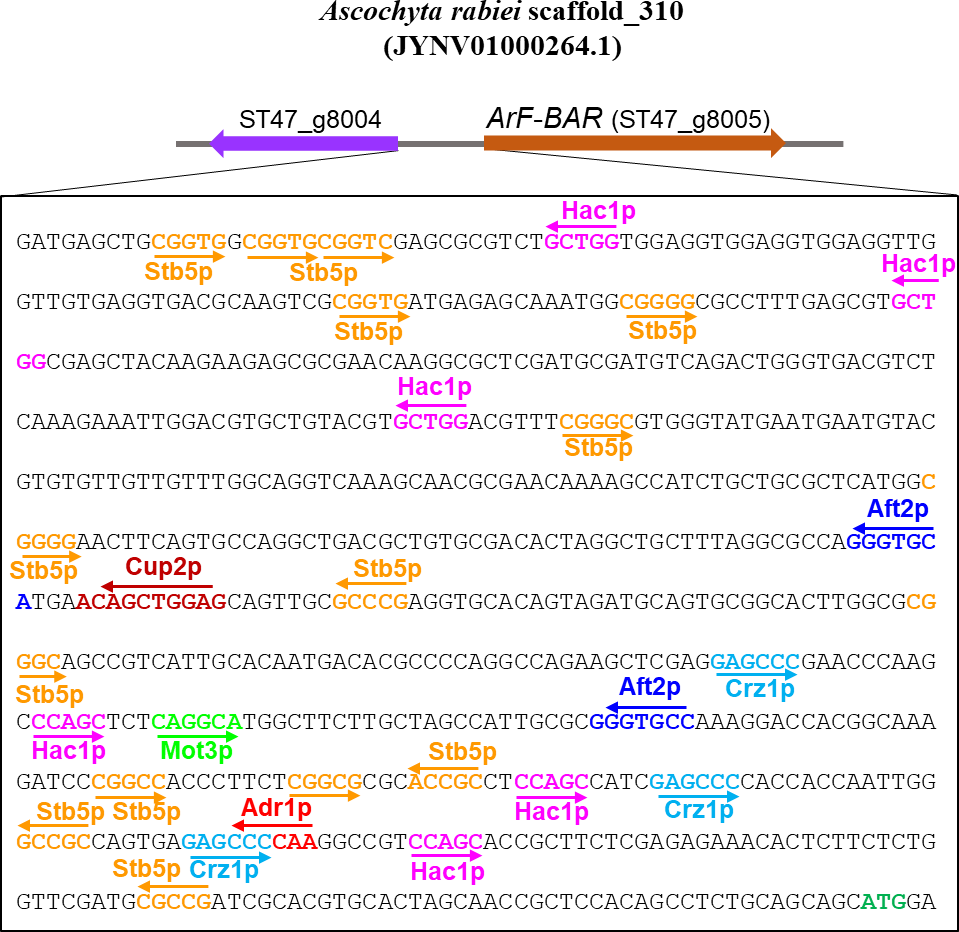

Supplement: S1 Table — (DOCX) [file pgen.1009137.s014.docx]
