# Supplementary material for: Modulation of fungal virulence through CRZ1 regulated F-BAR-dependent actin remodeling and endocytosis in chickpea infecting phytopathogen Ascochyta rabiei
Source: PLoS Genet. 2021 May 17;17(5):e1009137. doi: 10.1371/journal.pgen.1009137 (PMC8158962; doi:10.1371/journal.pgen.1009137)
Supplement: S2 Table — (DOCX) [file pgen.1009137.s015.docx]

**S2 Table. *Ascochyta rabiei* strains used in this study.**

| Strains | Relevant Genotype | Reference |
| --- | --- | --- |
| ArD2 (Indian type culture  collection no. 4638) | Wild-type | Verma et  al., 2016 |
| *∆arf-bar* | *ArF-BAR* deletion mutant of ArD2 | This study |
| *∆arf-bar/ArF-BAR* | Complemented transformants of *∆arf-bar* mutant with WT  *ArF-BAR*, under its native promoter | This study |
| *∆arf-bar/ArF-BAR mut1* | Complemented transformants of *∆arf-bar* mutant with F- BAR  domain mutation of *ArF-BAR* | This study |
| *∆arf-bar/ArF-BAR mut2* | Complemented transformants of *∆arf-bar* mutant with C1 domain mutation of *ArF-BAR* | This study |
| *ArF-BAR:EYFP* | Localization of ArF-BAR | This study |
| *ArRab5:EGFP and ArF-BAR:*  *mCherry* | Colocalization of ArF-BAR and ArRab5 | This study |
| *ArRAB7:EGFP* and  *ArF:BAR:mCherry* | colocalization of ArRAB7 and ArF-BAR | This study |
| *ArF-BAR -EGFP* and  *ArSNC1:*mCherry | colocalization of ArF-BAR and ArSNC1 | This study |
| *Lifeact:mCherry* | Localization of F-actin in WT | This study |
| *∆arf-bar /Life-*  *act:mCherry* | Localization of F-actin in *∆arf-bar* mutant | This study |
| *∆arcrz1* | *ArCRZ1* deletion mutant of ArDII | This study |
| *∆arcrz1/ArCRZ1* | Complemented transformants of ∆*Arcrz*1 mutant with WT  *ArCRZ*1, under its native promoter | This study |
| *ArCRZ1:EYFP* | Localization of ArCRZ1 | This study |
