# Supplementary material for: Modulation of fungal virulence through CRZ1 regulated F-BAR-dependent actin remodeling and endocytosis in chickpea infecting phytopathogen Ascochyta rabiei
Source: PLoS Genet. 2021 May 17;17(5):e1009137. doi: 10.1371/journal.pgen.1009137 (PMC8158962; doi:10.1371/journal.pgen.1009137)
Supplement: S3 Table — (DOCX) [file pgen.1009137.s016.docx]

**S3 Table: List of oligonucleotides used in this study:**

| **Oligo’s** | **Sequence (5′→ 3′)** | **RE(*)** |
| --- | --- | --- |
|  | **qRT-PCR of *ArF*-*BAR* gene** |  |
| ArF-BARRT1F | 5’ GGCAGCATCCATCGAAACTC 3’ |  |
| ArF-BARRT1R | 5’ GATCTCAGAGGAAAGGTGTTGCA 3’ |  |
| ArEFUTRF | 5’ GATCACTTCTTCGGTCGTTTGTT 3’ |  |
| ArEFUTRR | 5’ CTTCGTTCCACCAGACCGTAA 3’ |  |
|  | ***ArF*-*BAR* gene knockout mutant generation** |  |
| ArF-BARKOif5F | 5’ TATAGGGCGAATTGGGTACCTCTTGGATCTTCGATGGCGTGC 3’ | *Kpn*I |
| ArF-BARKOif5R | 5’ ATATCAGTTAACGTCTGCAGAGGCTGTGGAGCGGTTGCTAGT 3’ | *Pst*I |
| Ar72KO3 | 5’ CGGATCCGACAGTCGTTCAAGAACGCG 3’ | *Bam*HI |
| Ar72KO4 | 5’ CGAATTCGGAAGAGAAGCATGTGGAGC 3’ | *Eco*RI |
|  | ***ArF*-*BAR* gene knockout mutant confirmation** |  |
| Ar725’KOC1 | 5' GAATGTGCGCCTCGTACTTCAG 3' |  |
| Ar725’KOC2 | 5' TCAGCTGTGAGAAGTCGACACGC 3' |  |
| 5’KONst1 | 5' GCTAATAAGAGTCACACTTCGAGCG 3'(binds TrpC promoter) |  |
| 5’KONst2 | 5’ GTTGACCTCCACTAGCTCCAGC 3’(binds TrpC promoter) |  |
| 3’KONst1 | 5' CTCTATCAGAGCTTGGTTGACG 3'(binds *Hph* gene) |  |
| 3’KONst2 | 5' CAATCGTCCGATCCGGAGCC 3'(binds *Hph* gene) |  |
| Ar723’KOC1 | 5' GGACGAGGTGGGAAAGAGAAACTG 3' |  |
| Ar723’KOC2 | 5' ATCAAAGCAACGGAGAGCAAAGGC 3' |  |
|  | **Complementation of *ArF*-*BAR* mutant** |  |
| ArF-BARCompif3 | 5’ GCCCTGGGTTGAATTCCACCTCGACGTTGAACTCGGTC 3’ | *Eco*RI |
| ArF-BARfusionR | 5' GTCGACCTCCATGCTGCTGCAGAGGCTGTG 3' |  |
| ArF-BARfusionF | 5’ CAGCAGCATGGAGGTCGACATTGCG 3' |  |
| ArF-BARCompif4 | 5’ GGCCAGTGCCAAGCTTCCTCTAAACAAGTGTACCTGTGC 3’ | *Hind*III |
|  | **Site-directed mutagenesis of *ArF*-*BAR*** |  |
| ArF-BARMut1F | 5' CTCAGCGCACTCGCAGAGGAGTACTATGAGAAGAAG 3' |  |
| ArF-BARMut1R | 5' CTTCTTCTCATAGTACTCCTCTGCGAGTGCGCTGAG 3' |  |
| ArF-BARMut2F | 5' CGATACGAAGAGCTTGAGGAGCTCCACGGTGACTAC 3' |  |
| ArF-BARMut2R | 5' GTAGTCACCGTGGAGCTCCTCAAGCTCTTCGTATCG 3' |  |
| ArF-BARC1MutF | 5' GCGGCGACCGTATAGGGGGAGGGTCTGCAAAGGGATTC 3' |  |
| ArF-BARC1MutR | 5' GAATCCCTTTGCAGACCCTCCCCCTATACGGTCGCCGC 3' |  |
|  | **Cloning of *ArF*-*BAR* CDS in pET28a(+)** |  |
| pET-ArF-BARF | 5’ AATGGGTCGCGGATCCATGGAGGTCGACATTGCGC 3’ | *Bam*HI |
| pET-ArF-BARR | 5’ GGTGGTGGTGCTCGAGCTAGACCTGCTGAATGTAGTTTGC 3’ | *Xho*I |
|  | **Cloning of full-length *ArRAB5* CDS** |  |
| ArRab5F | 5’ ATGTCGCAGCAGAGAGTCCAG 3’ |  |
| ArRab5R | 5’ CTAGCAGTTGCAGCCTGCAG 3’ |  |
|  | **Cloning of *ArRAB5* tagged with EGFP in pBIF2** |  |
| Rab5ifR | 5’ AACGTTAAGTGGATCCCTAGCAGTTGCAGCCTGC 3’ | *Kpn*I |
| Rab5fusionF | 5’ GCTGTACAAGATGTCGCAGCAGAGAGTC 3’ | *Bam*HI |
|  | **Knock-out construct of *ArCRZ1* gene** |  |
| 5’crz1KOF | 5’ CGGGCCCCCCCTCGAGGCACACGTTGGTGGTGCCTGGATG 3’ | *Xho*I |
| 5’crz1KOR | 5’ ACGTCTGCAGAAGCTTGGCGTAGGGAAGCGTGCGGTTACG3’ | *Pst*I |
| 3’crz1KOF | 5’ GAATAGATCTGGATCCGTCGCTTCCTCTCAGTGAAGCACC3’ | *Bam*HI |
| 3’crz1KOR | 5’ TACCCAATTCGAATTCGCGCTTGAAGTGCTCGCCATGTAC3’ | *Eco*RI |
|  | **Knock-out confirmation of *ArCRZ1*** |  |
| Crzkoc5F1 | 5’ CTTGGAGGCTGTGGCTGTGCCAG 3’ |  |
| Crzkoc5F2 | 5’ GATAGAAGAACAGAAGTGGACGG 3’ |  |
| Crzkoc5R1 | 5’ GCTAATAAGAGTCACACTTCGAGCG 3’(binds TrpC promoter) |  |
| Crzkoc5R2 | 5’ CATTGTTGACCTCCACTAGCTCCAG 3’(binds TrpC promoter) |  |
| Crzkoc3F1 | 5’ CTCTATCAGAGCTTGGTTGACGGC 3’(binds *Hph* gene) |  |
| Crzkoc3F2 | 5’ CGACGCAATCGTCCGATCCGGAGCCG 3’(binds *Hph* gene) |  |
| Crzkoc3R1 | 5’ GACGAAGATGGACGACGACAGCGC 3’ |  |
| Crzkoc3R2 | 5’ TCTTCGGGCGGGCGAGCGTGTGGTG 3’ |  |
|  | **Complementation construct of *ArCRZ1*** |  |
| ArcrzcifF | 5’ ATCTCTGCAGTCTAGAGCACACGTTGGTGGTGCCTGGATG 3’ | *Eco*RI |
| ArcrzcifR | 5’ AAGTGGATCCACTAGTTTAGTTTCGCCCATCGTAGCTATC 3’ | *Hind*III |
|  | **Cloning of *ArCRZ1* in pET28a(+)** |  |
| pET:CRZ1F | 5’ AATGGGTCGCGGATCCATGGACGGCTTGCAGCAGCGCG 3’ | *Bam*HI |
| pET:CRZ1R | 5’ GGTGGTGGTGCTCGAGTTAGTTTCGCCCATCGTAGCTATC 3’ | *Xho*I |

|  | **Cloning of Lifeact fused with mCherry** |  |
| --- | --- | --- |
| LAmCF | 5’CCGAGCTCATGGGTGTCGCAGATTTGATCAAGAAATTCGAAAGCATCTCAAAGG AAGAAGGCTCGATGGTGAGCAAGGGCGAGGAGG 3’ | *Sac*I |
| LAmCR | 5’CCGGATCCCTACTTGTACAGCTCGTCCATGC 3’ | *Bam*HI |
|  | ***ArACTIN*** |  |
| ACTifF | 5’CTAAGAACGCGGCCATTACGGCCATGGAAGAGGAAGTCGCAGCC 3’ |  |
| ACTifR | 5’CCCCCGACATGGCCGAGGCGGCCAAGAAGCACTTGCGGTGGACGATG 3’ |  |
|  | **Localization of F-BAR domain of ArF-BAR** |  |
| ArF-BARBmcL1 | 5’ATCTCTGCAGTCTAGAATGGAGGTCGACATTGCGCCCCAG 3’ | *Xba*I |
| ArF-BARBmcL2 | 5’AACGTTAAGTGGATCCCTACTTGTACAGCTCGTCCATGCC 3’ | *Bam*HI |
|  | **Yeast two-hybrid Assays** |  |
| ArF-BARFLENF | 5' TCCGCGGCCGCCATGGAGGTCGACATTGCGCCCCAG 3' | *Not*I |
| ArF-BARFLENR | 5' GTCGGCGCGCCCGACCTGCTGAATGTAGTTTGCAG 3' | *Asc*I |
| ArBARENR | 5' GTCGGCGCGCCCCGTCTTCAACAAGACGTTCCGTAAG 3' | *Asc*I |
| ArSHENF | 5' TCCGCGGCCGCCCTTAGCTCCGGATACTCTGCAAC 3' | *Not*I |
| ArWASPENF | 5' GCAGGCTCCGCGGCCGCCATGCCCTCCATCCTGTCCGACGAG 3' | *Not*I |
| ArWASPENR | 5' AGCTGGGTCGGCGCGCCCCCAGTCGTCCTTGTCGGACTCATCG 3' | *Asc*I |
|  | **Electrophoretic mobility shift assay** |  |
| GRF | 5’CTCCAGCCATCGAGCCCCACCACCAATTGGGCCGCCAGTGAGAGCCCCAAGGCC GTCC 3’ |  |
| GRR | 5’GGACGGCCTTGGGGCTCTCACTGGCGGCCCAATTGGTGGTGGGGCTCGATGGCTG GAG 3’ |  |
| GRM1F | 5’CTCCAGCCATCGGGCCCCACCACCAATTGGGCCGCCAGTGAGGGCCCCAAGGCC GTCC 3’ |  |
| GRM1R | 5’GGACGGCCTTGGGGCCCTCACTGGCGGCCCAATTGGTGGTGGGGCCCGATGGCT GGAG 3’ |  |
| GRM2F | 5’CTCCAGCCATCGAGCAACACCACCAATTGGGCCGCCAGTGAGAGCAACAAGGCC GTCC 3’ |  |
| GRM2R | 5’GGACGGCCTTGTTGCTCTCACTGGCGGCCCAATTGGTGGTGTTGCTCGATGGCTG GAG 3’ |  |
| GRM3F | 5’CTCCAGCCATCGGGCAACACCACCAATTGGGCCGCCAGTGAGGGCAACAAGGCC GTCC 3’ |  |
| GRM3R | 5’GGACGGCCTTGTTGCCCTCACTGGCGGCCCAATTGGTGGTGTTGCCCGATGGCTG GAG 3’ |  |
|  | **Subcellular localization of ArF-BAR** |  |
| ArF-BARifLocF | 5’AGACATCAGAGCTCGGTACCATGGAGGTCGACATTGCGC 3’ | *Kpn*I |
| ArF-BARifLocR | 5’TGCTCACCATTCTAGAGACCTGCTGAATGTAGTTTGCAGG 3’ | *Xba*I |
|  | **Subcellular localization of ArCRZ1** |  |
| CRZloc1 | 5’TTGAGCAGACATCAGAGCTCATGGACGGCTTGCAGCAGCGCG 3’ | *Kpn*I |
| CRZloc2 | 5’TGCTCACCATTCTAGAGTTTCGCCCATCGTAGCTATCGCTC 3’ | *Xba*I |
|  | **Subcellular localization of ArSNC1** |  |
| ArSNC1ifF | 5’TTGAGCAGACATCAGAGCTCATGTCAAGCCGCGAGGAACC 3’ |  |
| ArSNC1ifR | 5’TGCTCACCATTCTAGATTTGTTTTTTGTCGCAACGAC 3’ |  |
