# Supplementary material for: Modulation of fungal virulence through CRZ1 regulated F-BAR-dependent actin remodeling and endocytosis in chickpea infecting phytopathogen Ascochyta rabiei
Source: PLoS Genet. 2021 May 17;17(5):e1009137. doi: 10.1371/journal.pgen.1009137 (PMC8158962; doi:10.1371/journal.pgen.1009137)
Supplement: Attachment — Submitted filename: PGENETICS-D-20-01505_reviewed-V1_reviewed.pdf [file pgen.1009137.s021.pdf]

PLOS Genetics

Modulation of fungal virulence through CRZ1 regulated F-BAR-dependent actin remodeling and endocytosis in chickpea infecting phytopathogen Ascochyta rabiei

--Manuscript Draft--

**Manuscript Number:**	PGENETICS-D-20-01505R1
**Full Title:**	Modulation of fungal virulence through CRZ1 regulated F-BAR-dependent actin remodeling and endocytosis in chickpea infecting phytopathogen Ascochyta rabiei
**Short Title:**	Role of CRZ1 regulated F-BAR in fungal virulence
**Article Type:**	Research Article
**Section/Category:**	General
**Keywords:**	Plant-fungus interactions, phtopathogenic fungi, calcineurin, effector
**Abstract:**	Polarized hyphal growth of filamentous pathogenic fungi is an essential event for host penetration and colonization. The long-range early endosomal trafficking during the hyphal growth is crucial for nutrient uptake, sensing of host-specific cues, and regulation of effector production. Bin1/Amphiphysin/Rvs167 (BAR) domain-containing proteins mediate fundamental cellular processes, including membrane remodeling and endocytosis. Here, we identified a F-BAR domain protein (ArF-BAR) in the necrotrophic fungus Ascochyta rabiei and demonstrate its involvement in endosome-dependent fungal virulence on the host plant Cicer arietinum . We show that ArF-BAR regulates endocytosis at the hyphal tip, localizes to the early endosomes, and is involved in actin dynamics. Functional studies involving gene knockout and complementation experiments reveal that ArF-BAR is essential for virulence. The loss-of-function of ArF-BAR results in delayed formation of apical septum from the growing hyphal tip, crucial for host penetration and impaired secretion of a candidate effector having signal peptide. The mRNA transcripts of ArF-BAR were induced in response to oxidative stress and infection. We also show that ArF-BAR is able to tubulate synthetic liposomes, suggesting the functional role of F-BAR domain in membrane tubule formation in vivo . Further, our studies identified a stress-induced transcription factor, ArCRZ1 (Calcineurin-responsive zinc finger 1) as key transcriptional regulator of ArF-BAR. We propose a model in which ArCRZ1 functions upstream of ArF-BAR to regulate A . rabiei virulence through a mechanism that involves endocytosis, effector secretion, and actin cytoskeleton regulation.
**Additional Information:**	
**Question**	**Response**
**Financial Disclosure**<br><br>Enter a financial disclosure statement that describes the sources of funding for the work included in this submission. Review the submission guidelines for detailed requirements. View published research articles from *PLOS Genetics* for specific examples.<br><br>This statement is required for submission and **will appear in the published article** if the submission is accepted. Please make sure it is accurate.	This research received funding from Council of Scientific and Industrial Research, Government of India, Grant number: 19/06/2016(i)EU-V to AS, Department of Biotechnology, Government of India, Grant number: BT/PR10605/PBD/16/791/2008, Department of Biotechnology, Government of India, Grant number: BT/AGR/CG-Phase II/01/2014 and National Institute of Plant Genome research to PKV. The funders had no role in study design, data collection and analysis, decision to publish, or preparation of the manuscript.

Powered by Editorial Manager® and ProduXion Manager® from Aries Systems Corporation

**Unfunded studies**<br><br>Enter: *The author(s) received no specific funding for this work.*<br><br>**Funded studies**<br><br>Enter a statement with the following details:<br>• Initials of the authors who received each award<br>• Grant numbers awarded to each author<br>• The full name of each funder<br>• URL of each funder website<br>• Did the sponsors or funders play any role in the study design, data collection and analysis, decision to publish, or preparation of the manuscript?<br>• **NO** - Include this sentence at the end of your statement: *The funders had no role in study design, data collection and analysis, decision to publish, or preparation of the manuscript.*<br>• **YES** - Specify the role(s) played.<br><br>* typeset	
**Competing Interests**<br><br>Use the instructions below to enter a competing interest statement for this submission. On behalf of all authors, disclose any competing interests that could be perceived to bias this work—acknowledging all financial support and any other relevant financial or non-financial competing interests.<br><br>This statement **will appear in the published article** if the submission is accepted. Please make sure it is accurate. View published research articles from *PLOS Genetics* for specific examples.	The authors have declared that no competing interests exist.

Powered by Editorial Manager® and ProduXion Manager® from Aries Systems Corporation

**NO authors have competing interests**<br><br>Enter: *The authors have declared that no competing interests exist.*<br><br>**Authors with competing interests**<br><br>Enter competing interest details beginning with this statement:<br><br>*I have read the journal's policy and the authors of this manuscript have the following competing interests: [insert competing interests here]*<br><br>* typeset<br><br>This statement is required for submission and **will appear in the published article** if the submission is accepted. Please make sure it is accurate and that any funding sources listed in your Funding Information later in the submission form are also declared in your Financial Disclosure statement.	
**Data Availability**<br><br>Authors are required to make all data underlying the findings described fully available, without restriction, and from the time of publication. PLOS allows rare exceptions to address legal and ethical concerns. See the PLOS Data Policy and FAQ for detailed information.<br><br>A Data Availability Statement describing where the data can be found is required at submission. Your answers to this question constitute the Data Availability Statement and will be published in the article, if accepted.	Yes - all data are fully available without restriction

**Important:** Stating 'data available on request from the author' is not sufficient. If your data are only available upon request, select 'No' for the first question and explain your exceptional situation in the text box.<br><br>Do the authors confirm that all data underlying the findings described in their manuscript are fully available without restriction?	
**Describe where the data may be found in full sentences. If you are copying our sample text, replace any instances of XXX with the appropriate details.**<br><br>• If the data are **held or will be held in a public repository**, include URLs, accession numbers or DOIs. If this information will only be available after acceptance, indicate this by ticking the box below. For example: *All XXX files are available from the XXX database (accession number(s) XXX, XXX.*).<br>• If the data are all contained **within the manuscript and/or Supporting Information files**, enter the following: *All relevant data are within the manuscript and its Supporting Information files.*<br>• If neither of these applies but you are able to provide **details of access elsewhere**, with or without limitations, please do so. For example:<br><br>*Data cannot be shared publicly because of [XXX]. Data are available from the XXX Institutional Data Access / Ethics Committee (contact via XXX) for researchers who meet the criteria for access to confidential data.*<br><br>*The data underlying the results presented in the study are available from (include the name of the third party and contact information or URL).*<br>• This text is appropriate if the data are owned by a third party and authors do not have permission to share the data.	All relevant data are within the manuscript and its Supporting Information files

* typeset	
Additional data availability information:	

Powered by Editorial Manager® and ProduXion Manager® from Aries Systems Corporation

Response to reviewers

Authors are thankful to the editor and anonymous reviewers for giving us positive and encouraging comments, and the opportunity to revise our research article manuscript (PGENETICS-D-20-01505). We hope that this improved manuscript will be accepted now for publication in PloS Genetics journal.

Response to Reviewer #1

General comments by Reviewer #1:

The paper setup is ok and the microscopy, and figures are pretty decent.

Response: Authors are thankful to the reviewer for the suggestions to improve the quality of our manuscript and words of encouragement. In the revised manuscript, we have made several changes in text and figures as per the overall suggestions of all the three reviewers. Point-by-point authors' responses on comments are given below;

Comment 1: The species name should include a mention of *Didymella rabiei*. Also, the pathovar should be indicated.

Response: *Didymella rabiei* is the teleomorph of *Ascochyta rabiei*, which is the anamorph. We had included '*Didymella rabiei*' name in the original manuscript under introduction section since both names are used in literature. We used *A. rabiei* mating type 2 (MAT1–2) isolate (ArD2; ITCC No. 4638) in the current study and based on Ascochyta blight disease development on chickpea differential lines it is 'pathotype II'. This is now mentioned in the revised manuscript's sub-section 'Fungal strains and growth conditions'. The same isolate was previously used in published articles for genome sequence (Verma et al., 2016; Scientific Reports 6, 24638) and resistance QTLs analysis in chickpea (Kumar et al, 2018; Plant Cell & Environ. 41:2128–2140) by our research group.

Comment 2: L 23-25 The authors state "The loss-of- function of ArF-BAR results in delayed formation of first septa from the hyphal tip, crucial for host penetration and proliferation" Yet, a direct microscopic observation of appressorium formation/penetration of the plant cuticula is not presented. Instead, the paper turns to genetic regulation of ArF-BAR and identifies ArCrz1. The importance of this regulation is not clear, however, as a role for ArCrz1 in virulence has not been determined.

Response: We have shown in Fig 5B that Δ*arf-bar* mutant has delayed septa formation. However, we have quantified this data to make it more convincing, which is presented in the revised manuscript Fig 5C graph. It shows that Δ*arf-bar* mutant has average size (length) ~3 times of 1st cell and ~2 times of 2nd cell from the hyphal tip as compared to wild-type *A. rabiei* after *in vitro* spore germination. We had analyzed the fungal growth of WT and Δ*arf-bar* mutant on plant host in several independent experiments. Based on those observations, we have shown in Fig 5A and Fig S7 that the penetration of chickpea cuticula and internal cells is reduced to half in Δ*arf-bar* mutant as compared to wild-type *A. rabiei*. We had quantified the depth of chickpea tissue penetration by WT and Δ*arf-bar* mutant strains till 48 hpi so that most of Δ*arf-bar* hyphae can have enough time to penetrate host tissue. Still, majority of the Δ*arf-bar* mutant hyphae failed to penetrate beyond 10–15 μm.

In the manuscript, we aimed towards showing role of ArF-BAR in fungal disease development on chickpea and other molecular aspects of ArF-BAR based on its domains. As per our literature survey, the transcriptional regulation of *ArF-BAR* gene has not been shown in any of fungal phytopathogen so we focused on its transcriptional regulation by ArCRZ1. In the manuscript, we showed the role of ArCRZ1 in *Ascochyta rabiei* virulence in Fig 9. Our result suggests that Δ*arf-bar* mutant is less virulent as compared to WT. It can be inferred from reduced number of *Ascochyta* blight disease lesions per plant and the lesion diameter.

Comment 3: Fig 1A: beyond a comparison in suppl figures S1 and S2 it is of more interest to see if the domain structure is also conserved. The ScBzz1/Las7 actually interacts with type I myosins…

Response: As the current manuscript deals with initial characterization of ArF-BAR, therefore, we focused ourselves to identify the role of *Ascochyta rabiei* F-BAR on pathogen growth and virulence on host tissue. The molecular functions of tubulation, co-localization with endocytosis markers, actin-polymerization, SH3 interaction and dimerization, presented in this manuscript are based on the domains present in the ArF-BAR. Reviewers may agree with us on the point that the identification of novel interacting partners for ArF-BAR and ascertain novel function to it can itself become an independent study on ArF-BAR. We hope to carry out such interesting study in near future independent of the current manuscript.

Comment 4: Fig 1B, the authors show that expression of *ArF-BAR* is induced upon germination. Yet they state that this equal, "transcript induction during pathogenesis". I tend to disagree.

Response: The Fig 1B of our manuscript represents the qRT-PCR data of relative *ArF-BAR* transcript level expression in mycelia after menadione treatment as compared to the non-treated control mycelia. Here, the transcripts get induced due to oxidative stress (menadione treatment). However, we have shown the *ArF-BAR* transcript level expression during the host hyphae/mycelia growth on host tissue in Fig 1C. The time points selected in Fig 1C are up to 72 hrs post conidial inoculation on chickpea host. As during chickpea-*Ascochyta* interaction, basal defence response of chickpea generates oxidative stress, so we have presented this data in Fig 1C and *in vitro* oxidative stress treatment data in Fig 1B. Hope, now the reviewer will agree that *ArF-BAR* transcript show higher expression during oxidative stress (*in vitro* menadione treatment) and during proliferation on host tissue.

Comment 5: The mutant shows slow growth (Fig. 2). This could reduce virulence by itself as an indirect effect. This should be discussed more clearly.

Response: The Δ*arf-bar* mutant growth on PDA plate after 7 days of incubation show reduced growth (Fig 2A and Fig 3; radial diameter on PDA plates). However, our repeated analysis of *in vitro* conidial hyphae growth does not show such significant difference during early growth up to 48 hrs post conidial germination although septa formation is delayed. However, other stresses (like osmotic stress) during *in vitro* growth on PDA plates (up to 7 days) may hamper mycelial growth. The Δ*arf-bar* mutant strain was found to be defective in effector secretion as compared to WT in a new experiment performed during the revision (Fig 5D). Now, it is clear that inability of mutant strain to cope with the host generated stresses may reduce Δ*arf-bar* mutant growth and penetration of the host tissue. Thus, *in vitro* growth reduction in Δ*arf-bar* mutant is not comparable to the growth on host plant tissue. Although, indirect effect of delayed septation on virulence cannot be neglected that we have included this in the 'discussion' section of the revised manuscript.

Comment 6: Figure 2C shows that there are a few lesions formed in the plant virulence assay. Thus, the authors should not state that ArF is essential for virulence, but required for full virulence as stated in L179+.

Response: We agree with reviewer's suggested sentence and have modified it in the revised manuscript.

Comment 7: L172, Typical AB disease symptoms were observed "what is meant with AB?"

Response: *A. rabiei* infects all the above ground parts of the chickpea plant. Initially, disease appears as water-soaked lesions on the leaves, which at later stage turns brown. Disease symptoms become visible as 'small circular necrotic lesions' on leaves and 'elongated brown lesions' on stems. These are called typical *Ascochyta* blight symptoms. However, in the revised manuscript we have elaborated this point in more detail for the general readers.

Comment 8: L191, Again, the authors state that *ArF-BAR* is indispensable for fungal pathogenicity. This is double ungood. First, it is dispensable, but required for full virulence; second, the authors make an extrapolation on fungal virulence in general that is not warranted. In light of the results also the PKC domain is required for full virulence, a point the authors just dropped.

Response: We have toned down on some of these statements that talk about pathogenicity. In L191, of the original manuscript, we wanted to convey that *ArF-BAR* gene is required for full virulence as its mutant shows reduced *Ascochyta* blight (AB) symptoms (Fig 2B-D) on chickpea. Besides this, the complementation of Δ*arf-bar* mutant with *ArF-BAR*^{mut1} construct also has similar level of reduced symptoms. Hence, in L191, we also wanted to convey that ArF-BAR domain residues are important for virulence on chickpea. We have made appropriate changes in the revised manuscript as per your suggestions. The role of C1 domain of ArF-BAR (wrongly marked as PKC domain in the previous version of manuscript as it showed high similarity with C1 domain of PKC1 protein) has also been discussed in *Ascochyta rabiei* virulence.

Comment 9: The genetic analysis is ok – yet, there may be issues with protein stability of the mutants that at least need to be discussed.

Response: We had not tagged the complementation constructs with any of the epitope so checking protein stability is difficult. We avoided epitope tag so that in complementation constructs the protein expresses similar to wild-type. However, fluorescence of chimeric mutants with eYFP (Fig 4A) rules out the question of mutant proteins stability. We hope reviewer must be now satisfied that mutant proteins are stable. We have discussed the same in revised manuscript.

Comment 10: L193-205, the observed effect of reduced penetration could also be a consequence of the slower growth, this needs to be discussed.

Response: Our views on this point may have been cleared by our response to your comment#5. Still, we have appropriately taken this point in the discussion section of revised manuscript considering delayed septa formation in Δ*arf-bar* mutant.

Comment 11: L206, ArF-BAR contains a PKC domain. To me this hint to a cell wall remodelling role. Oxidative stress is not the first stress that comes to mind then but rather osmotic stresses.

Response: We regret for mentioning C1 domain (~50 amino acids) as PKC domain in the original manuscript as explained in the author response of your comment#8. Now, this anomaly has been corrected in the revised manuscript. We did not mention osmotic stress during conidial germination on host tissue as during conidial inoculation enough humidity is maintained in growth chambers. The chickpea aerial tissue also traps lot of moisture due to high trichome density. In field conditions too, the conidia start germinating on chickpea host only with the help of moisture from dew/ intermittent rain in the winter growing season. We hope now that the reviewer will agree with our reason of not considering osmotic stress. Although, role of ArF-BAR in cell wall remodeling can't be ruled out and the same has been integrated in 'discussion' section of the revised manuscript.

Comment 12: L206-221: if the authors want to stress this, they should move the data from supplemental to results. I suggest to be more critical with these results. Stats need to include the general growth defect of the mutant and recalculate the relative growth inhibition on top of that.

Response: We agree with the reviewer and have appropriately placed the figure in main results. The point of relative growth inhibition is also well taken. The growth inhibition by menadione and H_2O_2 (in %; relative to PDA only) has been shown in the revised manuscript Fig S8.

Comment 13: L225: how was transient expression of YFP achieved. This is not clear from the methods section.

Response: In our study, the expression of all the T-DNA constructs in *Ascochyta rabiei* was performed by stable transformation by *Agrobacterium* mediated T-DNA transfer and antibiotic selection. The 'transient expression' was inadvertently written in this sentence and

the word has been appropriately corrected in the revised manuscript. A published reference of *A. rabiei* transformation (Nizam et al. 2010; Curr Genet. 56:391–399.) has also been provided with the revised text.

Comment 14: L238-245: a statistic on the distribution of septa in the hyphae seems warranted to see if the spacing is random, irregular or if the compartments are simply larger than in WT.

Response: A full statistic data on the length of 1st and 2nd cell (in µm) from hyphae tip is now presented in the revised manuscript Fig 5B and 5C.

Comment 15: The YFP data would be improved by a co-staining of the actin cytoskeleton. This would show that ArF-BAR colocalizes with actin patches.

Response: To visualize actin in WT and Δ*arf-bar* mutant, we had tagged the mCherry fluorescent protein with Lifeact, a 17-amino-acid peptide, which decorates filamentous actin (F-actin) structures in eukaryotic cells and tissues (Fig 7D). To better visualize the colocalization of actin patches with EYFP in ArF-BAR::EYFP transformed *A. rabiei* cells, the hyphae were co-stained with Alexa Fluor™ 635 Phalloidin (Catalog number: A34054, ThermoFisher Scientific). However, after repeated experiments we could not observe the dye labelled signals of actin cytoskeleton after fixation as the fluorescence was not properly obtained. This may be due to the technical glitches as *A. rabiei* fixation protocol is not well standardized.

Comment 16: The colocalization data of ArF-BAR with Rab5 and not with Rab7 is interesting. The suppl material on Rab7 needs to be moved to the results next to Rab5.

Response: As per suggestion, we have appropriately moved the Rab5 and Rab7 data together in Fig 6B and 6C of the revised manuscript.

Comment 17: Overall: The paper comes with a ton of results. This is very nice. Yet, the authors also produce a ton of figures and suppl material that overwhelms and distracts from the novel findings. The authors should better indicate what is known from ArF homologs and build on that. The knowns may go to supplemental and the novel stuff should be highlighted in the figures.

Response: We agree on the overall views expressed by the reviewer and accordingly placed the data and improved manuscript text too. We hope that the new experiments carried out

during revision and implementations of suggestions from all three reviewers have significantly improved the quality of our revised manuscript.

Response to Reviewer #2

General comments by Reviewer #2:

In my opinion, the work done is of interest for the readers of PloS Genetics. However, some points need to be addressed before I recommend its publication.

Response: We thank reviewer for giving his positive comments about our manuscript and study. We have taken a note of all your queries and suggestions along with other reviewers' views to improve our revised manuscript.

Comment 1: Although the manuscript is well written, it needs improvement and rewriting of several sentences.

Response: We have made several changes as per your suggestions mentioned in the original manuscript pdf file. We extend our thanks for such detailed analysis of our manuscript and hope you will find these points appropriately corrected in the revised manuscript.

Comment 2: For a better characterization of ArF-BAR, the double point mutant should be generated and its phenotype compared with those of Mut1 and Mut2.

Response: In our study, we generated knock-out mutant (Δ*arf-bar*) to characterize the role of *ArF-BAR* in *Ascochyta rabiei* pathogen. The Δ*arf-bar* mutant showed drastically reduced virulence as compared to WT (Fig 2B-D). As the ArF-BAR protein has two important domains of F-BAR and C1, we generated mut1 and mut2 complementation constructs to check the roles of these two domains, individually. However, the lack of intact F-BAR (residues of F-BAR mutated in complementation construct mut1) and Δ*arf-bar* mutant showed comparable reduced virulence (Fig 2B-D) as compared to the mut2 complementation construct. Therefore, we believe that generation of double mutations complementation construct (mut1 and mut2 together) would not reduce *A. rabiei* virulence beyond Δ*arf-bar* mutant. We hope the reviewer will now agree with our clarification.

Comment 3: The apical localization of ArF-BAR should be tracked in more detail. Additional markers of exocytosis and the Spitzenkörper should be used with that aim.

Response: We have examined the ArF-BAR localization in more detail during the period of this revision by tagging ArF-BAR with YFP and transforming it with ArSNC1::mCherry marker (Fig 4C). The SNC1 has been used extensively in living fungal cells (Valkonen et al. 2007; J Biol Chem. 282:22775–22785) and *M. oryzae* (Giraldo et al. 2013; Nat Commun. 4:1996) as a marker for exocytosis marker. The result is now presented in Fig 4C of the revised manuscript. More number of markers for endocytosis, exocytosis and Spitzenkörper are yet to be standardized for *A. rabiei* since the N-terminal or C-terminal tagging and overexpression of chimeric proteins also changes their localization pattern.

Comment 4: How do the authors explain the difference in ArF-BAR localization in Figure 3A compared to that shown in Figure 4B? Is it only a matter of the Z-position? In addition, the authors should indicate the hyphal region plotted in the graph of panel 4B.

Response: The difference is original manuscript's Fig 3A and 4B (Revised manuscript's Fig4A and 6B) is only due to the Z-position and different regions of hyphae targeted during image acquisition. The difference between Fig 4A and Fig 6B is about magnification and region of hyphae/mycelium. The Fig 4A, at higher magnification, of the hyphae is to show vesicle formation towards the growing tip, while in Fig 6B, the same phenomenon is shown at the other phase/growth region of mycelia at lower magnification. Otherwise, the localization of mCherry and YFP tagged ArF-BAR remains similar. The Fig 6C can be also considered for better comparison of all the three figures. The hyphal region plotted in fluorescence intensity graphs has been now been marked with a magenta line in the merged images of revised Fig 6B and 6C.

Comment 5: I recommend to tone down any mention to a hypothetic direct role of ArF-BAR in endocytosis. I would say that what the authors have shown is a defect in endocytosis in the null arf-bar mutant, and that not necessarily means that ArF-BAR is directly involved in early endosome biogenesis and endocytosis.

Response: We have taken note of your point and appropriately corrected the sentences in the revised manuscript.

Comment 6: I would show the phenotype of the null crz mutant under stress conditions as a panel of Figure 7 and not as supplementary material.

Response: The data shown for Δ*arcrz1* phenotype under stress conditions in Fig S14 (original manuscript's Fig S13) was just to ascertain that *ArCRZ1* behaves similar under to the other published stresses in fungal system. Hence, we have kept the data in supplementary data so that new data about *A. rabiei* genes remain in main figures. We hope you will agree with our explanation.

Comment 6: Finally, I recommend a more detailed description of some of the procedures used. I attach a pdf file of the manuscript with all my comments and corrections. Hope it will help.

Response: The authors are very much obliged by such detailed evaluation and point to point suggestions in the pdf file. We have improved upon all your suggestions in the revised manuscript.

Authors Response to Reviewer # 3

General comments by Reviewer # 3:

Generally, I found this manuscript enjoyable to read. The study is well executed, and well written. But I have a few comments. I feel that the text has to explain experiments and conclusions in more detail. It lacks specificity when stating conclusions and when experiments are described. Improving this will help strengthening the manuscript.

Response: Authors thank the anonymous reviewer for praising our manuscript. Your words of praise have made us set up some new experiments for the revision. We have made changes in the revised manuscript as per your suggestions. Hope you will applaud these changes in the revised manuscript. Below we have written our responses to your concerns and explained about the changes that we have made in the revised manuscript.

Major Comment 1a: In paragraph from L123- 132, and Fig.1B authors show that ArF-BAR expression is triggered by oxidative stress. In Fig.1C authors show that the relative gene expression of ArF-BAR is increased at 24 hpi which is the time at which spores germinate on the host surface. Does this mean that at 24h the fungus has not penetrated the host tissue? According to Fig.S7 the formation of primary invasive hyphae happens at 48h. How is the developmental stage of the interaction between the fungus and the host over a time course?

The way I understood this was that oxidative stress happened after penetration of the host tissue. Please could authors maybe provide detailed micrographs of a time course of the invasion of the host? Also, why ArF-BAR will be induced just at the host surface?

Response 1a: Our group has been studying *Ascochyta rabiei* development on various accessions of chickpea (susceptible, tolerant and resistant). A general finding of our group and other researchers is that on chickpea foliar tissue, the conidia start germinating on most chickpea accessions at ~ 6 hpi under suitable conditions of moisture and temperature. Although a variation in germination between biological replicates and isolates is observed on chickpea host accessions, which is acceptable. Generally, the *A. rabiei* spores germinate at different rate from 6 to 18 hpi. However, most hyphae that germinated early starts penetrating host tissue ~24 hpi and about the same time an increased H_2O_2 accumulation has been observed at site of fungal penetration. Our qRT-PCR results on *ArF-BAR* transcript expression suggested that its transcripts get induced ~5 fold at 1 h post menadione treatment of fungal hyphae (Fig. 1B) while on chickpea host it gets induced ~6 fold at 24 hpi (Fig. 1C). The H_2O_2 accumulation in chickpea host at ~24 hpi near the sites of penetration and highest accumulation of *ArF-BAR* happens around the same time. This observation suggests that on chickpea tissue, the host basal defense response generated oxidative stress or H_2O_2 initiates the fungal signaling for *ArF-BAR* transcriptional-level induction. The original EST clone (*Ar72*) of *ArF-BAR* was also isolated as an menadione induced transcript from *A. rabiei* mycelium through suppression subtractive hybridization (SSH) EST library (Singh *et al.*, 2012; PLoS ONE 7(3): e33128).

In Fig S7, the WT and Δ*arf-bar* are compared for growth and penetration of host tissue at 48 hpi by observing the penetrations at different depths (Z: 0 to -23 μm). The previous observations of a slower growth of Δ*arf-bar* strain as compared to *A. rabiei* WT strain on PDA plates persuaded us to consider 48 hpi as the time point so that most of Δ*arf-bar* hyphae can have enough time to penetrate host tissue. As per our observation for WT strain, many primary invasive hyphae have penetrated host tissue ~24 hpi and started proliferating between epidermal cells while for Δ*arf-bar* the hyphae have not penetrated below 10-15 μm. This can be due to mutant (Δ*arf-bar*) incapability to grow under the adverse conditions of host-generated defenses and inability to suppress host defense.

To examine a possible reason behind it, we performed secretion of a candidate effector having classical signal sequences in WT and Δ*arf-bar* mutant. In a new experiment performed during the manuscript revision, we have shown that Δ*arf-bar* mutant has defect in fungal effector secretion (Fig 5D). The details of this experiment are mentioned in our

response to your comment #1C and main text of the revised manuscript. The results of this new experiment clearly suggest that Δ*arf-bar* mutant has impaired secretion of effectors or other secreted biomolecules. This impaired secretion may result in its inability to properly strengthen its cell wall or inability to overcome the stresses caused by host basal defense leading towards a slower growth on chickpea tissue. We have incorporated the new experiment results and made several changes in the revised manuscript as per suggestions in your comment to make it understandable for readers.

Major Comment 1b: Also, null mutants of Δ*arf-bar* were not able to penetrate more than 9.83 µm. This could suggest that the mutant has a defect in coping with oxidative stress inside the plant.

Response 1b: We agree with reviewer on this point as the Δ*arf-bar* mutant strain showed less growth under H_2O_2 and menadione treatments as compared to WT and complemented strains on the PDA plates Fig 3 (Fig S8 of original manuscript). Beside this defect, the Δ*arf-bar* mutant strain is defective in effector secretion as revealed by the newly incorporated results obtained during the revision of manuscript (explained in response to your comment #1C). This inability to secrete effectors, resulting into less suppression of host defense response, may be responsible for delayed growth and limited penetration of host tissue. The cell wall integrity of fungal penetrating hyphae can also be questioned as cell wall repair is important for hyphal protection. These reasons have been discussed in text of the revised manuscript.

Comment 1c: Can authors stain with Diaminobenzidine (DAB) to see if ROS production is increased in Δ*arf-bar* during epidermis infection? It might well be that as Δ*arf-bar* is impaired in growth and also in coping with plant defence responses, have authors tested whether Δ*arf-bar* has impaired secretion?

Response 1c: This is a nice suggestion and we tried the same in various chickpea-*A. rabiei* interaction studies with 3,3′-Diaminobenzidine (DAB) but could only observe H_2O_2 accumulation in the chickpea cells, around the site of fungal invasion, and not inside the fungal cells (Singh *et al.*, 2012; PLoS ONE 7(3): e33128). Technically it is very difficult to monitor intracellular H_2O_2-level fluctuations inside *Ascochyta rabiei* cells during chickpea epidermis growth with staining methods like DAB and 2′,7′-dichlorodihydrofluorescein diacetate (H_2DCFDA). The main difficulty with H_2DCFDA is that fluorogenic dye signals cannot be differentiated between chickpea host and fungal cells.

We have considered reviewer's suggestion and introduced a new experiment to check whether Δ*arf-bar* mutant has impaired secretion of fungal effectors. Since, effectors help in successful colonization of plant tissue through various modifications in host cells. In this exocytosis experiment, a candidate *A. rabiei* effector (Ar93; unpublished data), possessing a typical secretory signal peptide was transformed into Δ*arf-bar* mutant and WT *A. rabiei* strains. The effector was constitutively expressed in WT and Δ*arf-bar* mutant strains with a c-terminal epitope to detect chimeric effector protein in the culture filtrate. The secreted proteins from *A. rabiei* cells were concentrated from culture filtrate. The total cell lysate of fungi was used as expression control along with untransformed strains as negative controls. We were unable to detect secreted Ar93 effector in the culture filtrate of Δ*arf-bar* mutant in various biological replicates. However, in wild type the Ar93 effector was secreted (Fig 5D). These results point towards the inability of Δ*arf-bar* mutant to secrete Ar93 or similar effectors. Therefore, impaired secretion of effectors or other secreted components must be resulting in Δ*arf-bar* mutant's slow growth and inability to cope with the chickpea basal defense as compared to WT. The entire new experiment has been incorporated in revised manuscript.

Major Comment 2: The tubulation micrograph of Fig. S3B is much better than the one used on the main Fig 1D-G. Please can you provide better images for Fig 1? It will be also good to include zoomed areas such as in Fig. S3B to show the difference between the mutants and the wild type, as current images are not very clear. Also, please can this be quantified to denote the relevance? Tubular vs. Non-tubular

Response 2: In the revised manuscript, we have changed Fig 1D-G images as per your suggestion so as to provide a better observation of liposome tubulation. Also, we have provided the quantitative data on tubulation in the updated Fig 1H, when liposomes are incubated with buffer control, bacterial purified ArF-BAR protein and its mutant versions. The data clearly shows significant differences in tubulation.

Major Comment 3: Authors have not found differences in liposome tubulation when W428 and L430 residues were mutated, however what happens if the entire PKC domain is deleted? Maybe those exact residues are not purely relevant, but the study of the entire domain could provide more substantial information. What will happen to the pathogenicity, the localization?

Response 3: We regret for the mistakenly marking C1 domain as PKC domain in Fig 1A. Now in updated Fig 1A, it has been corrected to C1 domain, which is ~ 50 amino acids domain showing similarity to the C1 domain of PKC1. The mutations of W428 and L430 (in *ArF-BAR*^{mut2}) were created as per previous observation that they are important in C1 domain of hPKC1 (Heung et al. 2004; 279: 21144-21153). Besides the conserved zinc-finger forming cysteine and histidine residues in C1 domain of ArF-BAR, the amino acids W428 and L430 plays a key role in maintaining the DAG-binding groove and the surrounding hydrophobic wall. Thus, they were mutated accordingly. We have checked the localization of ArF-BAR^{mut2} and as compared to WT and it shows no significant difference in localization (Fig 3A). The complementation of Δ*arf-bar* mutant with *ArF-BAR*^{mut2} construct has defect in virulence (revealed by decreased lesions and their size in Fig. 2C and 2D) as compared to the complementation of Δ*arf-bar* mutant with WT *ArF-BAR* construct. Therefore, we believe that mutated C1 domain residues play some role in fungal virulence. The wrong marking of PKC domain in ArF-BAR, instead of C1 domain only, created doubts which we regret.

Major Comment 4: Further explanation on how the list of putative transcription factors regulating ArF-BAR should be included. It is very good that a list of candidate transcription factors was identified. However, it is necessary to explain better what was used as query to identify this list? Was CRZ1 just selected based on the relationship with stress responses or was there any other evidence? Were other TFs tested? More elaboration on this part is necessary as it is such as important finding that it is hard to understand from the manuscript how CRZ1 was identified. Elaborate materials and methods section too.

Response: We agree with your observation on these points and have tried to explain all the mentioned points in the revised manuscript text under results, discussion and methods section.

Major Comment 5: Show localization of ArF-BAR, Rab5 and Rab7 in Δ*crz1* mutant. If CRZ1 mutant operates upstream of ArF-BAR, they should be mis-localized.

Response: In our study, we have observed that *ArCRZ1* regulates *ArF-BAR* at transcriptional level and not at protein-protein interaction level. The ArCRZ1 mediated transcription regulation of *ArF-BAR* is proved by protein-DNA interaction and checking expression of *ArF-BAR* transcripts in Δ*crz1* mutant. The *ArCRZ1* knock-out mutant has reduced or no expression of *ArF-BAR* (Fig. 9B). Hence, ArF-BAR localization was not checked in Δ*crz1* mutant. Moreover, ArCRZ1 regulates other genes too in filamentous fungi so the mis-

localization of ArF-BAR, Rab5 and Rab7 (if found in suggested experiments) could not be directly assigned to the ArCRZ1 and ArF-BAR signaling. Although FM 4-64 uptake assay in WT and Δ*arf-bar* mutant shows that ArF-BAR regulates endocytosis (Fig 6A).

Major Comment 6: In the model, authors suggest that upon ROS perception, intracellular Ca^{2+} increases to activate CRZ1. Authors show that CRZ1 localizes to nuclei during infection (Fig. S12B). I feel that Fig. S12B in which the nuclear localization of CRZ1 is shown during invasive growth should be in the main figure. It is an important and fits with the model. I think it is necessary to show better imaging and include a DIC panel. Also, over a time course, is CRZ1 translocated once the fungus has penetrated the host tissue penetrates?
Response: As per your suggestion, we have shifted Fig S12B (of the original manuscript) to the main article figure (Fig. 8E). The figures have been revised and incorporated in revised manuscript (Fig 8E). After conidia germination on chickpea host, we have seen that *ArCRZ1* localizes very early into nucleus of hyphal cells, possibly due to various unknown stresses on host tissue (Fig. 8E) while during normal *in-vitro* growth of *A. rabiei* it primarily localizes in cytoplasm and moves to nucleus after calcium treatment (Fig 8D).

Minor point 1: L115- reference 27 doesn't correspond to the reference of the genome.
Response: The mistake has now been corrected in the revised manuscript. The *A. rabiei* genome sequence article referred is Verma et al., 2016 Scientific Reports. The reference is at position [25] in the revised manuscript.

Minor point 2: L171- instead of infection bioassay, it will be better to specify that it is a spray inoculation.
Response: The sentence has been corrected as per your suggestion and now it mentions 'conidial suspension spray inoculation'.

Minor point 3: L225-ArF-BAR::EYFP was transiently expressed? Could authors state how, which promoter? According to Fig. S5 is native promoter. This needs clarification.
Response: The sentence was inadvertently written wrong and we have revised and corrected it in the updated manuscript. The ArF-BAR::EYFP's stable ectopic expression was achieved by *Agrobacterium tumefaciens*-mediated transformation (ATMT), where T-DNA cassette of chimeric construct under *ArF-BAR*'s native promoter was integrated into the genome of Δ*arf-bar* mutant strain.

Minor point 4: Fig1B- was this done in mycelium treatment with menadione or spores? State it in the legend of the figure. Also, please could authors state what was the standard used for the qPCR?

Response: Thank you for pointing this mistake in the legend of Fig 1B. The menadione treatment was performed after growing conidia for 7 days. The legend has been appropriately corrected in the revised manuscript. The *A. rabiei* elongation factor alpha (*EF1*α) was used as an internal standard for normalization in qRT-PCR. The same was mentioned in original manuscript under 'RNA extraction and expression analysis' subheading of materials and methods.

Minor point 5: Fig S10B- Line scan legend very small. Please increase font.

Response: We have improved this figure by increasing the font size of axis units and symbols. The fig S10B of original manuscript has now been shifted to main results (Fig 6C) of the revised manuscript.

Minor point 6: Y2H experiments need to be explained better, at least in the legend or in materials and methods. Please state what are the controls too.

Response: The Y2H experiments were performed using split-ubiquitin based Dualhunter system and modified vectors that have been previously explained in detail in our published article (Purayannur et al., 2017; Scientific Reports 7:5026). However, to make our manuscript more understandable, we have explained Y2H experiments in more detail in revised manuscript.

Minor point 7: State what is A.U. in the figure legends.

Response: The A.U. was fluorescence intensity unit that has been updated in revised figures (Fig 6A-C).

Minor point 8: L129- "Consistent with previous observations"

Response: By mistake we have not included the reference of previous article (Singh et al., 2012; PLoS ONE 7(3): e33128) where the clone of *ArF-BAR* was originally isolated and checked for *in-planta* transcript expression after *A. rabiei* conidial suspension inoculation on a susceptible chickpea variety Pusa 362. The reference has now been included at this position in the revised manuscript.

Minor point 9: Legend panel D fig 1 is missing.

Response: In Fig. 1D the liposomes were not supplemented with any protein and only protein resuspension buffer was added. We have modified the legend of Fig 1D for clarity.

Minor point 10: Was fluorescence intensity calculated per area? e.g. Figure 4.

Response: In Fig. 6A (Fig 4B of the original manuscript) the fluorescence intensity was calculated per area. However, in Fig. 6B and 6C (Fig 4B and S10B of the original manuscript), we forgot to add the line through which the fluorescence intensity was measured in merged image. We have made changes accordingly in the revised manuscript.

Minor point 11: Explain legend and controls Fig. 5

Response: The legend of Fig 7A and 7B (Fig 5 of original manuscript) has been revised to make better explain the controls and experiment of split-ubiquitin Y2H assay.

Minor point 12: Fig. S4. Southern blot, explain better the output of the sizes.

Response: Thanks for pointing this issue. We have explained the output sizes in the Southern blot of Δ*arf-bar* and Δ*arcrz1* mutant strains in supplementary data of the revised manuscript.

Manuscript Click here to access/download;Manuscript;Manuscript.doc

Modulation of fungal virulence through CRZ1 regulated F-BAR-dependent actin remodeling and endocytosis in chickpea infecting phytopathogen *Ascochyta rabiei*

Manisha Sinha[1¶], Ankita Shree[1¶], Kunal Singh[1#], Kamal Kumar[1], Shreenivas Kumar Singh[1], Vimlesh Kumar[2], Praveen Kumar Verma[1, 3*]

[1]Plant Immunity Laboratory, National Institute of Plant Genome Research, Aruna Asaf Ali Marg, New Delhi-110067, India.

[2]Department of Biological Sciences, Indian Institute of Science Education and Research Bhopal (IISER-Bhopal), Bhauri, Bhopal-462066, Madhya Pradesh, India

[3]Plant Immunity Laboratory, School of Life Sciences, Jawaharlal Nehru University, New Delhi-110067, India

[¶]These authors contributed equally to the work

[*]Corresponding author

[#]Present address: Biotechnology Division, CSIR-Institute of Himalayan Bioresource Technology, Palampur-176 061, Himachal Pradesh, India.

Abstract

Polarized hyphal growth of filamentous pathogenic fungi is an essential ev[illegible] for host penetration and colonization. The long-range early endosomal trafficking during the hyphal growth is crucial for nutrient uptake, sensing of host-specific cues, and regulation of effector production. Bin1/Amphiphysin/Rvs167 (BAR) domain-containing proteins mediate fundamental cellular processes, including membrane remodeling and endocytosis. Here, we identified a F-BAR domain protein (ArF-BAR) in the necrotrophic fungus *Ascochyta rabiei* and demonstrate

its involvement in endosome-dependent fungal virulence on the host plant *Cicer arietinum*. We show that ArF-BAR regulates endocytosis at the hyphal tip, localizes to the early endosomes, and is involved in actin dynamics. Functional studies involving gene knockout and complementation experiments reveal that ArF-BAR is essential for virulence. The loss-of-function of ArF-BAR results in delayed formation of apical septum from the growing hyphal tip, crucial for host penetration and impaired secretion of candidate effector having signal peptide. The mRNA transcripts of ArF-BAR were induced in response to oxidative stress and infection. We also show that ArF-BAR is able to tubulate synthetic liposomes, suggesting the functional role of F-BAR domain in membrane tubule formation *in vivo*. Further, our studies identified a stress-induced transcription factor, ArCRZ1 (Calcineurin-responsive zinc finger 1) as key transcriptional regulator of ArF-BAR. We propose a model in which ArCRZ1 functions upstream of ArF-BAR to regulate *A. rabiei* virulence through a mechanism that involves endocytosis, effector secretion, and actin cytoskeleton regulation.

Author summary

BAR-domain superfamily is known to mold amorphous lipid bilayer into defined tubular shapes and required for endosome formation and trafficking. Although these proteins were studied earlier in context of their structural and biochemical properties, there is limited evidence on the direct role of F-BAR domain proteins in the pathophysiological development of other economically important fungi. Our study assumes functional significance for plant infection as we identified an F-BAR domain-containing protein that is regulated by a distinct transcriptional network regulated by CRZ1. We characterized F-BAR in a necrotrophic fungal pathogen, *Ascochyta rabiei*, which causes the *Ascochyta* blight disease in chickpea plants. Additionally, we

have also identified a calcium-regulated CRZ1 transcription factor that regulates the transcription of *ArF-BAR*. Our study will help to understand a signaling cascade that regulates the endosome formation required for fungal virulence.

Introduction

Polarized hyphal growth is a signature feature of filamentous fungi during host colonization [1]. This feature allows the fungus to sense, coordinate, and respond to an array of cues from the host [2]. Therefore, regulation of hyphal tip growth is one of the major virulence determinants in filamentous fungi. In response to infection, the plant innate immune system recognizes pathogen and fosters effective defense response. Pathogens must recognize the plant surface cues and counter host-generated defense responses for effective pathogenesis. Additionally, invasive fungi must overcome an intracellular challenge posed by the distance between the elongated invading hyphal architecture and the nucleus [3]. Mounting evidence strongly suggests that the complications associated with this increased distance are overcome by long-distance intracellular communication for rapid and precise transduction of external information [4]. Moreover, the maintenance of extremely polarized hyphal morphology is heavily dependent on endosome trafficking. It is also important to maintain the structural and functional features of a fungal cell [5].

In the case of filamentous fungi, long-distance signaling is mediated by endosomes. Besides signal sensing for motor-dependent retrograde signaling, they are involved in the recycling of cell wall components, polarisomes, and various receptors required for polarized tip growth [6]. Loss of these functions leads to impaired host invasion and virulence [7, 8]. The generation of early endosomes (EEs), which are multipurpose carriers, is a key step in the

endocytic pathway and involves the cooperative action of membrane bending and cytoskeleton reorganization. Membrane bending is the cornerstone for the generation of EEs and is regulated by proteins involved in the detection and stabilization of membrane curvature [9,10] .

In animal cells, BAR domain superfamily proteins have been shown to integrate membrane dynamics with cytoskeletal changes [11]. The F-BAR domain proteins form N-terminal α-helical coiled-coil dimers and bind to negatively charged lipid membranes via their positively charged residues. This binding generates membrane curvature and regulates intracellular vesicle trafficking [12]. Depending on the degree of curvature in dimer of BAR proteins, the BAR membrane domain superfamily proteins are broadly classified into three families: classical BAR and N-BAR, Fer/CIP4 homology BAR (F-BAR) and inverse BAR (I-BAR) proteins. The classical BAR domain (N-BAR) and F-BAR domain proteins induce positive membrane curvature through concave lipid-binding interfaces and trigger cell membrane invagination. However, I-BAR domains interact with shallow negatively curved membranes through convex lipid-binding interfaces, leading to cell membrane protrusion [13, 14].t Pioneering work in the corn smut fungus, *Ustilago maydis*, revealed the importance of endocytosis for the pathogenic development and virulence of filamentous fungi by showing impaired early pathogenicity and germination in endocytic mutants [15]. The F-BAR protein, Cdc15, is involved in cytokinetic ring and septa formation in *U. maydis* [16]. Recent studies in *Magnaporthe oryzae* revealed the importance of N-BAR domain-containing proteins in the growth and virulence of filamentous fungi [17]. Further, the I-BAR protein, Rvs167, was found to be involved in the extension of the rigid penetration peg required during *M. oryzae* invasion [18]. The F-BAR-like protein Bzz1p in yeast and Cip4 in *Drosophila* acts during the early stages of endocytosis in the formation of actin patches [19,20]. It triggers actin polymerization via the

Arp2/3 complex [21]. There has been an intense study on the structural and biochemical properties of BAR domain proteins that contribute to their mode of action [11,13]. However, there has been limited functional characterization of BAR superfamily proteins in phytopathogenic fungi.

Ascochyta rabiei (Pass.) Labr. [teleomorph *Didymella rabiei*, ArD2; ITCC No. 4638], a causal agent of *Ascochyta* blight (AB) disease in chickpea (*Cicer arietinum* L.), is one of the most devastating necrotrophic phytopathogens. *A. rabiei* infects the above-ground parts of this legume plant and greatly reduces the yield of the crop [22]. The fungal hyphae aggregate in the cortical cells of the chickpea plant and differentiate into asexual spores called pycnidia [23,24]. The genome of *A. rabiei* has been sequenced and analyzed to identify pathogenic determinants [25]. *A. rabiei* has emerged as an interesting model system for elucidating the cell biology, especially the endocytic machinery, during polar growth and pathogenesis in necrotrophic phytopathogenic fungi.

In this study, an F-BAR domain-containing protein, ArF-BAR, was identified and characterized in *A. rabiei*. A loss of function mutation of ArF-BAR cause, delayed septa formation, severely compromised fungal virulence on chickpea and secretion of Ar93, which is a candidate effector of *A. rabiei* having a classical signal peptide sequence. The results showed that the F-BAR domain of the ArF-BAR protein binds to and deforms synthetic liposomes and generates membrane tubules. *ArF-BAR* transcripts were induced in response to oxidative stress and infection, and localized to endocytic vesicles within the fungal hyphae. It was also found that *ArF-BAR* expression was regulated by a stress inducible transcription factor, ArCRZ1. Our data suggested that ArF-BAR-dependent actin cytoskeleton dynamics combined with endocytosis and effector secretion is crucial for *A. rabiei* virulence.

Results

ArF-BAR expression is induced in response to oxidative stress and infection

Transcriptome analysis of *A. rabiei* during oxidative stress has provided a greater understanding of the survival strategies used by necrotrophic fungi against host-generated oxidative stress [26]. The study by Singh et al., [26] revealed that 70 unigenes were upregulated under oxidative stress conditions. Of these 70 unigenes, a clone resembling "ST47_g8005" of the sequenced *A. rabiei* genome [25], showed early upregulation against oxidative stress. The deduced amino acid sequence of this gene revealed a protein with four distinct domains: An N-terminal F-BAR domain (7–253 amino acids), a unique C1 domain (407–459 amino acids) showing similarity to the C1 domain of Protein Kinase C1 (PKC1), and two consecutive C-terminal SH3 domains (572–651 and 705–760 amino acids) (Fig 1A). Henceforth, this protein has been named as ArF-BAR. Phylogenetic analysis of selected pathogenic fungi and other eukaryotes revealed that ArF-BAR shared sequence identity with proteins of many closely related fungal pathogens (S1 Fig). ArF-BAR was found to share approximately 33% and 41% sequence identity with *Saccharomyces cerevisae* BZZ1p and *Drosophila melanogaster* Cdc42-Interacting Protein 4 (CIP4) proteins, respectively (S2 Fig).

To validate the results of the transcript profiling, quantitative real-time PCR (qRT-PCR) was performed using primers specific to the F-BAR domain encoding region. The qRT-PCR experiments revealed a higher expression level of the *ArF-BAR* transcript in *A. rabiei* mycelia after a 1 h treatment of menadione, which is an oxidative stress generator (Fig 1B). To directly assess *ArF-BAR* transcript induction during pathogen proliferation on a susceptible chickpea variety (PUSA-362), the time course of *ArF-BAR* transcript expression was measured using qRT-PCR after inoculating plants with *A. rabiei* conidial suspension. Consistent with the

previous observations [26], a significant increase in the *ArF-BAR* transcript level was found following infection. The maximum transcript level was at 24 hours post-inoculation (hpi; Fig 1C), which is within the critical time period for spores to germinate on the host surface and early germinated conidial hyphae starts penetrating chickpea tissue [24,27].

ArF-BAR is a membrane tubulating protein

To understand whether ArF-BAR has a functional F-BAR domain, synthetic liposomes containing Rhodamine B-conjugated PE were incubated with purified His-tagged recombinant ArF-BAR protein (S3A Fig). The spherical liposomes transformed into an intense network of narrow tubules within 30 min of incubation with ArF-BAR and ArF-BAR^{mut2} (Fig 1D and 1E). This indicated that ArF-BAR was capable of robust liposome tubulation activity (S3B Fig). Additionally, the sequence alignment of ArF-BAR with that of other characterized F-BAR domain-containing proteins, revealed the presence of positively charged conserved residues (S2 Fig). In previous studies, the positively charged residues of FBP17 and Syndapin, have been shown to interact with negatively charged phospholipids to induce membrane curvature [28, 29]. Since ArF-BAR has conserved positively charged amino acids at positions 57, 58, 131 and 132 (S2 Fig), the role of these residues in membrane deformation were evaluated. This evaluation was performed by replacing the residues with glutamate (K57E, K58E, R131E and K132E) via site-directed mutagenesis, and the mutant version was referred to as ArF-BAR^{mut1} (S4B Fig). As expected, it was found that substituting the conserved lysine/arginine with glutamate abolished the tubulation activity of the F-BAR domain (Fig 1F). Protein kinase C1 (PKC1) proteins are known to interact with diacylglycerol (DAG), which has a role in membrane interactions. To abolish the involvement of the unique C1 domain of ArF-BAR in membrane deformation, residues at positions W428 and L430 were replaced with glycine [30], and the mutant form was

referred to as ArF-BAR^{mut2} (S4C Fig). Further, this mutated protein was used to assess the *in vitro* liposome tubulation activity, and a similar activity to that of the ArF-BAR protein was found (Fig 1G). Thus, positively charged amino acids of F-BAR domain are involved in ArF-BAR mediated membrane tubulation (Fig 1H). To test whether the tubulation activity of ArF-BAR correlated with its lipid binding ability, a liposome co-sedimentation assay was used (Fig 1I). Compared to the native ArF-BAR protein, ArF-BAR^{mut1} showed reduced lipid sedimentation efficiency. In contrast, the sedimentation was unaffected with ArF-BAR^{mut2}. This indicated the importance of the direct interaction of the BAR domain with the lipids in liposome tubulation (Fig 1H and 1I). Together, these results suggest that F-BAR domain of ArF-BAR, plays a major role in interacting with the lipid membrane for binding and deformation.

ArF-BAR is required for *A. rabiei* virulence on chickpea

To elucidate the biological importance of *ArF-BAR* in fungal virulence, an *A. rabiei* knockout mutant (Δ*arf-bar*) was generated that lacked the entire protein coding region of *Arf-BAR* gene. The *ArF-BAR* gene was targeted for deletion using a homologous recombination approach. The results confirmed that the open reading frame (ORF) of *ArF-BAR* had been successfully replaced with a single copy of the hygromycin resistance gene (*Hph*; S5A and S5B Fig). Simultaneously, Δ*arf-bar* mutant strain was complemented with a T-DNA cassette that expressed *ArF-BAR* under its own promoter (Δ*arf-bar/ArF-BAR*; S4 and S5 Fig).

To check the effect of *ArF-BAR* deletion on vegetative growth of *A. rabiei*; wild-type (WT), Δ*arf-bar*, and knockout complemented (Δ*arf-bar/ArF-BAR*) strains were grown on potato dextrose agar (PDA) plates. The knockout mutants showed reduced radial growth compared to *A. rabiei* wild-type (WT) and this radial growth was restored in the Δ*arf-bar/ArF-BAR* (Fig 2A).

To determine the *Ascochyta* blight (AB) disease development and virulence of the fungal strains, the conidial suspensions of each strain were spray inoculated on susceptible chickpea variety plants individually. The typical AB disease symptoms, described previously [23,24], were observed on chickpea plants challenged with the *A. rabiei* wild-type (WT) and Δ*arf-bar/ArF-BAR*, but not on plants challenged with Δ*arf-bar* (Fig 2B). The degree of pathogenicity was quantified according to lesion number and lesion size, which were compared among the *A. rabiei* wild-type (WT) and mutants at 144 hpi. The lesion number per plant in Δ*arf-bar* was significantly lower than in the *A. rabiei* wild-type (WT) (Fig 2[illegible]The mean lesion size was also much lower in Δ*arf-bar* than in the *A. rabiei* wild-type (WT) (, Fig 2D). However, for the Δ*arf-bar/ArF-BAR* complementation mutant, the disease symptoms were comparable to those of the *A. rabiei* wild-type WT. Overall, the disruption of the *ArF-BAR* gene significantly impaired the pathogenicity of *A. rabiei*.

To ascertain the role of F-BAR and unique C1 domains in fungal virulence, the two domains were independently inactivated by site-directed mutagenesis as described earlier. The Δ*arf-bar* knockouts were complemented with residues mutated in F-BAR domain (Δ*arf-bar/ArF-BAR*$^{\text{mut1}}$) and C1 domain (Δ*arf-bar/ArF-BAR*$^{\text{mut2}}$; S4B, S5B and S5C Fig). The in virulence of Δ*arf-bar/ArF-BAR*$^{\text{mut1}}$ strain was very less as compared to the WT. Meanwhile, in the case of Δ*arf-bar/ArF-BAR*$^{\text{mut2}}$, the number of lesions per plant was significantly lower than in the WT, but there was no significant difference in the size of the lesions (Fig 2C and 2D). The severity of the disease symptoms increased in the plants after 10 days post infection (dpi), but no significant differences were observed for plants challenged with Δ*arf-bar* strain (S6 Fig). Together, these results confirm that *ArF-BAR* is an important virulence determinant and that the positively charged F-BAR domain residues are required for full-virulence of *A. rabiei*.

It was subsequently hypothesized that the reduced virulence in Δ*arf-bar* could have resulted from at least two factors: a) knockout mutants may have had a compromised ability to penetrate host tissue, or b) the reduced virulence of the mutant was a consequence of a reduction in fungal viability or inability to counter the host basal defense. To address these possibilities, hyphal penetration was examined and compared by inoculating chickpea leaves with the WT and Δ*arf-bar* mutant strains. The leaves of the chickpea plants challenged with respective strains were subjected to wheat germ agglutinin-Alexa Fluor conjugate (WGA-AF488) staining. At 48 hpi, the infected leaves were stained with WGA-AF488 to enable visualization of the fungus [31]. The infected leaves were optically sectioned using confocal microscopy starting from the surface of the leaves. It was observed that hyphae of the *A. rabiei* wild-type (WT) strain efficiently penetrated into the chickpea leaves up to depths of 20.25 µm ± 3.92 (mean ± SD; n = 3) (S7A and S7C Fig). In stark contrast, the hyphae of the Δ*arf-bar* mutant were unable to penetrate beyond 9.83 µm ± 2.54 (mean ± SD; n = 3, S7B and S7C Fig). Thus, the depth and efficiency of hyphal penetration by Δ*arf-bar* were significantly impaired.

Further, to investigate the direct involvement of *ArF-BAR* in fungal viability under oxidative stress conditions, radial growth assays were performed for WT, Δ*arf-bar*, Δ*arf-bar/ArF-BAR*, Δ*arf-bar/ArF-BAR*^{mut1} and Δ*arf-bar/ArF-BAR*^{mut2} strains. These strains were inoculated on either potato dextrose agar (PDA) or PDA supplemented with menadione (250 µM and 500 µM) or 2 mM H_2O_2. The diameter of radial growth was analyzed at 10 dpi. The mycelial growth of Δ*arf-bar* was reduced compared to that of the WT. The mycelial growth was restored in the complemented Δ*arf-bar/ArF-BAR* and Δ*arf-bar/ArF-BAR*^{mut2} mutants (Fig 3A). However, the mycelial growth of complemented Δ*arf-bar/ArF-BAR*^{mut1} was unable to match that of the WT. Exposure to oxidative stress led to further growth inhibition in Δ*arf-bar* (Fig 3). The

involvement of *ArF-BAR* in oxidative stress tolerance was confirmed by the complementation, Δ*arf-bar/ArF-BAR*, which showed comparable resistance to that of the WT (Fig 3). Further, exposure to oxidative stress led to greater growth inhibition in Δ*arf-bar/ArF-BAR*^{mut1} than in the wild-type. However, the growth inhibition of Δ*arf-bar/ArF-BAR*^{mut2} was similar to that of wild-type. The negative effect on growth of fungal strains till 10 dpi was more pronounced for H_2O_2 as compared to menadione (Fig 3; S8 Fig). These results strongly support the hypothesis that *ArF-BAR* is required for full virulence of *A. rabiei* wild-type (WT) on susceptible chickpea host and are required for coping with the host basal defense, mainly oxidative stress

Absence of *ArF-BAR* perturbs septa formation

Having a potential role in fungal virulence, the next aim was to determine the subcellular localization of ArF-BAR inside *A. rabiei* wild-type (WT) hyphal cells. In this regard, expression of a chimeric protein, ArF-BAR::EYFP, which was achieved by *Agrobacterium tumefaciens*-mediated transformation (ATMT) of Δ*arf-bar* strain [27]. The bioimaging of the enhanced yellow fluorescent protein tagged ArF-BAR showed punctate distribution throughout the cytoplasm of the fungal hyphae. The chimeric protein was mostly concentrated at the growing hyphal tip and at the septa (Fig 4A and 4B). Fungal hyphae transformed with chimeric ArF-BAR^{mut1}::EYFP exhibited disrupted localization of these punctate structures and the fluorescence was completely diffused throughout the cytoplasm. However, the distribution of the fluorescent puncta was unaffected in hyphae of strains stably expressing ArF-BAR^{mut2}::EYFP (Fig 4A). In this strain, the punctate structures were distributed throughout the cytoplasm and were prominently concentrated at the growing hyphal tip (Fig 4A). Further, to validate the spatiotemporal distribution pattern of the ArF-BAR protein during infection, fungal spores expressing ArF-BAR::EYFP were allowed to infect susceptible chickpea stem peel. Microscopic

observation of these stem peel infected with fungal hyphae revealed a distribution pattern that was similar to that of the ArF-BAR::EYFP expressing hyphae growing on glass slides; the chimeric protein was predominantly distributed at the hyphal tip and septa (S9 Fig). The localization of ArF-BAR::EYFP towards the membrane of growing hyphae prompted us to check its localization with a Spitzenkörper marker ArSNC1,which is a vesicle-bound v-SNARE protein and known for its role in polarized exocytosis in conjugation with other proteins [32-34]. The *A. rabiei* wild-type (WT) ArSNC1 was identified based on its high homology with the *M. oryzae* SNC1 (MGG_12614.6) encoded protein. However, The localization pattern of ArF-BAR::EYFP and ArSNC1::mCherry confirms the apical localization of ArF-BAR (Fig 4C). The delayed proliferation Δ*arf-bar* strain compared to that of the WT, on susceptible plant leaf surface and less penetration of host tissue (Fig 5A; S7 Fig) points towards mutant strain's defect in normal development or suppression of host defense. The *in vitro* germination and growth of WT and Δ*arf-bar* conidia, up to 12 h on hydrophobic surface showed marginal difference (S10 Fig). However, the first cell from the growing hyphal tip in Δ*arf-bar* was observed to be elongated that may be due to delayed septation as ArF-BAR localizes to the septa. The arrangement of septa within the hyphae of both the WT and the Δ*arf-bar* strain was examined in a number of growing fungal tips. Microscopic analysis of the Δ*arf-bar* mutant using calcofluor white, which precisely stains cell wall components, showed that the hyphae lacked regularly spaced septa (Fig 5B). Interestingly, the distance of the first septum from the growing hyphal tip (polarized end) was significantly greater in the *Δarf-bar* mutant (58.5µm ± 4.65; mean ± SEM) than in the WT (23.5µm ± 3.23; mean ± SEM; Fig 5C).It showed that Δ*arf-bar* mutant, as compared to *A. rabiei* wild-type (WT), has more length of first cell and second cell from the

hyphal tip (Fig 5C). Overall, these results reveal that the ArF-BAR gene is necessary for appropriate hyphal architecture, which is in turn, important for host penetration and virulence

Δ*arf-bar* impairs secretion of a candidate effector secretion

The substantial reduction in virulence of Δ*arf-bar* on chickpea has led us to hypothesize that the deletion mutant may have impaired in the effector secretion ability of *A. rabiei* wild-type (WT). To further confirm the possible role of ArF-BAR in the effector secretion, a candidate secretory effector Ar93 (Accession number: GW996416) [26] possessing a classical signal peptide for secretion was ectopically expressed in WT and Δ*arf-bar* background. The secretion of Ar93 protein fused with EYFP::FLAG in media was examined using proteins from culture filtrate and cell lysates of WT and Δ*arf-bar*. The western blots analysis of culture filtrate and cell lysate of axenically grown stable fungal transformants (WT and Δ*arf-bar*) showed exclusive effector secretion in WT. However, the Ar93 was observed in cell lysates of both the WT and Δ*arf-bar* (Fig 5D). Thus, we conclude that Δ*arf-bar* mutant is impaired in secretion of Ar93 or similar effectors possessing classical secretory signal peptide.

Δ*arf-bar* mutant has impaired endocytosis

Since F-BAR domain proteins are known to dimerize and form a canonical banana-shaped fold [35], This evidence further indicates the evolutionarily conserved nature of the BAR proteins (S11 Fig). To gain insight into the role played by ArF-BAR in endocytosis, an amphiphilic styryl type endocytic tracer dye, N-(3-triethylammoniumpropyl)-4-(p-diethyl-aminophenyl-hexatrienyl)pyridiniumdi bromide (FM4-64), was used [36]. The FM 4-64 staining of WT and Δ*arf-bar* and its microscopic analysis revealed visible internal staining of the hyphae. The results suggested rapid internalization of dye in WT hyphae, compared to *Δarf-bar* strain where no

such internalization observed even after 10–15 min of FM4-64 incubation (Fig6A). To validate these findings, the mean fluorescence intensity area was determined in biological replicates (details in sub-section ‘quantification and statistical analysis’ of Materials and Methods). The result suggested that the endocytic activity from plasma membrane is impaired in Δ*arf-bar* (Fig 6B). The result provides evidence for the involvement of *ArF-BAR* in the endocytosis.

The central components of the endocytic pathway are the EEs, where the small GTPase Rab5 plays a major regulatory role in biogenesis [37]. *ArRab5*, an orthologue of *Rab5*, was identified in the *A. rabiei* genome using *Rab5* of *M. oryzae* and *U. maydis* as query at NCBI. The relationship between ArF-BAR-associated endocytosis and ArRab5-associated early endosomes was determined using a double-labeling experiment. *ArF-BAR* was tagged with *mCherry* and *ArRab5* with *EGFP*. This was followed by sequential transformation into the WT. The coalescence of fluorescence obtained showed perfect positive correlation between the two fusion proteins (Fig 6B).

Early endosomes mature to late endosomes followed by the replacement of Rab5 by Rab7 [38]. Thus, we aimed to determine whether the punctate distribution of ArF-BAR was associated with all endocytic vesicles or specifically to the EEs. In this context, similar to *Rab5* orthologue, an orthologue of *Rab7* was identified in *A. rabiei* and was tagged with EYFP (*ArRab7:EYFP*). A similar double-labeling experiment was performed that showed separate intensity and localization of ArF-BAR::mCherry and ArRab7::EYFP chimeric proteins (Fig 6C). In summary, these results uncover the role of ArF-BAR as it associates with early endocytic vesicle protein complexes and mediate endocytosis.

ArF-BAR modulates the actin cytoskeleton

The presence of SH3 (SRC homology 3) domains in F-BAR proteins is well documented for their relationship with the actin cytoskeleton via interactions with the Arp2/3 complex activator Wiskott-Aldrich syndrome protein (WASp) [39]. Since, ArF-BAR in *A. rabiei*, contains two consecutive SH3 domain at its C-terminu, we initially hypothesized for the probable existence of interactions of ArF-BAR with ArActin.g Y2H system, it was shown that ArF-BAR does not interact directly with actin (Fig 7A), rather it physically interacts with WASp through its SH3 domains (Amino acids 572-760; Fig 7B; S12A and S12B Fig). Further, to check the role of ArF-BAR in actin polymerization, a well-established *in vitro* actin polymerization assay was performed. The kinetics of actin polymerization was monitored by increase in the fluorescence of pyrene-labeled actin. The effect of purified ArF-BAR protein on actin nucleation (actin, Arp2/3 and WASp) was tested using a minimal set of components for all reactions. Interestingly, the addition of purified recombinant protein led to an increase in the actin polymerization rate (Fig 7C). By increasing the concentration of purified protein, a significant gradual activation in the rate of actin polymerization was observed (Fig 7C). These results strongly suggest that ArF-BAR plays an active role in WASp-dependent actin polymerization.

Appropriate organization of actin is required for vesicular dynamics, organelle movement and cytokinesis. Actin microfilaments or F-actin are organized into higher order structures comprising of actin patches, cables and rings that serve as the track for long distance transport [40]. To elucidate the relative importance of ArF-BAR in actin organization, the actin dynamics was compared in the WT and Δ*arf-bar* mutants. Here, we took advantage of Lifeact, an actin binding peptide fused with a fluorescence protein. Lifeact has been successfully employed for *in vivo* visualization of actin filaments and dynamics in a variety of organisms including fungi and

plants [41]. In this study, a *Lifeact:mCherry* fusion construct was generated and transformed into both the WT and Δ*arf-bar* mutant to visualize cytoplasmic actin dynamics in fungal hyphae. Confocal microscopy revealed discrete actin patches and cables in the WT hyphae, while actin patches were rarely visible in the Δ*arf-bar* mutant (Fig 7D and 7E). Moreover, the actin cables were dramatically disorganized in the Δ*arf-bar* mutant. Although ArF-BAR does not directly interact with actin, it regulates actin polymerization and actin cytoskeleton through its association with WASp in growing fungal hyphae.

ArCRZ1 is a potential transcriptional regulator of *ArF-BAR*

Thus far, the findings of the present study have revealed the importance of *ArF-BAR* in the regulation of endocytic pathways, which is crucial for the pathogenesis of *A. rabiei*. However, the transcriptional regulatory machinery associated with the endocytic pathway in filamentous pathogenic fungi is largely unknown. Hence, the transcriptional mechanism associated with the regulation of *ArF-BAR* expression in response to infection was subsequently analyzed. The *in-silico* binding motifs for fungal (TFs) were identified in the upstream regulatory sequences of *ArF-BAR*. The YEASTRACT online tool [42] revealed seven different putative TF binding sites (S1 Table) in 1 kb promoter region of *ArF-BAR* gene. Among these, three binding sites were found for calcineurin-responsive zinc finger transcription factor 1 (CRZ1), positioned at -106, -136, and -254 upstream to the ArF-BAR translational start site ATG (Fig 8A) .

CRZ1 is an evolutionarily conserved TF from yeast to mammals. CRZ1 was chosen for analysis because it regulates the expression of various genes involved in stress tolerance [43] and is widely known to translocate inside the nucleus with an increase in cytosolic Ca^{2+} ion concentration [44]. *Ascochyta rabiei* *gene* (*ArCRZ1*; ST47_g3738) encodes a protein that possesses a serine-rich region (SRR), two consecutive calcineurin docking domains (CDD),

characterized by PxIxIT motif (PRILPQ and PEINID) and a single C_2H_2 zinc finger motif (S13A Fig). To determine the role of ArCRZ1 in the transcriptional regulation of *ArF-BAR*, the binding of ArCRZ1 to the regulatory sequences of *ArF-BAR* was confirmed. This confirmation was performed via an electrophoretic mobility shift assay (EMSA) using recombinant His-tagged ArCRZ1 proteins. Shifting was observed for the DNA fragment possessing the calcineurin-dependent response element (CDRE) in the presence of purified His-ArCRZ1. However, mutations in this CDRE resulted in the abolishment of binding (Fig 8B and 8C).

Subsequently, the sub-cellular localization of ArCRZ1 under Ca^{2+} and oxidative stress conditions was determined. The *A. rabiei* wild-type (WT) was transformed with a translational fusion of *ArCRZ1* with *EYFP* towards C-terminus. After, 0.2 M $CaCl_2$ treatment, confocal microscopy revealed nuclear localization of the ArCRZ1::EYFP signal (Fig 8D). Interestingly, in absence of any treatment, the ArCRZ1::EYFP fusion protein was uniformly distributed within the cytoplasm during normal growth on glass slides (Fig 8D). Since, the nuclear translocation of CRZ1 is mediated by a phosphatase (calcineurin) [45], a chemical genetics approach was used to confirm the relationship between calcineurin and ArCRZ1. A potent calcineurin inhibitor, FK506 (Tacrolimus) was used to silence the enzymatic activity of calcineurin [43]. FK506 potentially inhibited nuclear translocation of ArCRZ1::EYFP, which suggests that calcineurin plays a role in the nuclear translocation of ArCRZ1 under stress conditions (Fig 8D).To corroborate and extend these findings during infection, susceptible plants were challenged with fungal conidia expressing ArCRZ1::EYFP and nuclear localization chimeric protein was observed (Fig 8E). Overall, chemical genetic results confirm the evolutionarily conserved regulation of ArCRZ1 by calcineurin under stress conditions.

To uncover the transcriptional regulation of the *ArF-BAR* gene mediated by ArCRZ1, a targeted deletion of the *ArCRZ1* gene was generated (Δ*arcrz1*), followed by complementation of mutant with *ArCRZ1* controlled by native promoter (Δ*arcrz1/ArCRZ1*; S13C,-S13D Fig). Interestingly, no significant difference in radial growth diameter was observed between Δ*arcrz1* mutant and *A. rabiei* wild-type (WT) (Fig 7A). The expression pattern of *ArF-BAR* in the WT and Δ*arcrz1* was analyzed using semi-quantitative RT-PCR. The results clearly showed significant reduction in *ArF-BAR* transcription in the *Δarcrz1* mutant (Fig 9B). Imposing oxidative stress to both the WT and the Δ*arcrz1* mutant via menadione treatment revealed an upregulation of the *ArF-BAR* transcript in the wild-type. However, this upregulation was found to be completely abolished in the Δ*arcrz1* mutant (Fig 9B). Overall, these results substantiate that under oxidative stress condition, the transcriptional regulation of *ArF-BAR* gene is mediated by the activated ArCRZ1.

Loss-of-function of ArCRZ1 compromises virulence similar to *Δarf-bar*

As *ArF-BAR* gene was transcriptionally regulated by ArCRZ1, it was hypothesized that the pathogenicity phenotypes of Δ*arcrz1* should be similar to those of the Δ*arf-bar* mutants. Consistent with this hypothesis, it was observed that *Δarcrz1* mutants displayed compromised virulence during chickpea infection bioassay. Results showed that the number and size of lesions were lower in the Δ*arcrz1* mutant than in the *A. rabiei* wild-type (WT). This virulence defect was rescued in Δ*arcrz1/ArCRZ1* complementation strain (Fig 9C–9E). The radial growth patterns of Δ*arcrz1* mutants grown on PDA supplemented with Ca^{2+}, menadione and sodium dodecyl sulfate (SDS) were also monitored. Under these tested stress conditions, the growth inhibition was significantly greater for Δ*arcrz1* than for *A. rabiei* wild-type (WT). However, the growth phenotype was restored in the Δ*arcrz1/ArCRZ1* strain, which suggests that *ArCRZ1* plays

a crucial role in calcium ion signaling, oxidative stress response and maintaining cell-wall integrity during infection (S14 Fig). Taken together, infection assay and stress treatments demonstrate that ArCRZ1 is a key regulator of the ArF-BAR, which further regulates various cellular processes like endocytosis, effector secretion and promotes fungal survival under host generated stress condition. ArCRZ1 induces the expression of *ArF-BAR* by directly binding to its *cis*-regulatory region and is critical for *ArF-BAR* dependent virulence.

Discussion

Dynamic membrane remodeling is essential for maintaining the integrity and identity of cells and cellular compartments [40]. Biological macromolecules, such as BAR superfamily proteins, can sense or induce membrane curvature. They are well-known modulators of transient membrane deformation [39]. Emerging evidence strongly suggests that EEs are crucial for long-distance intracellular communication. Thus, EEs and their roles have broad implications for a wealth of cellular processes such as growth, development, and virulence in filamentous fungi [4,46,13]. The results of the present study further corroborate this conclusion and provide new information regarding signaling and transcriptional control of F-BAR proteins in phytopathogenic fungi. To date, our understanding of the role played by F-BAR proteins in membrane curvature generation and efficient long-range endosome trafficking in fungi during plant-pathogen interactions remains very limited.

To best of our knowledge, this study provides the evidence that an F-BAR domain protein act as a key mandate for virulence of a phytopathogenic fungi. Based on the evidences provided in the present study, we propose that ArF-BAR performs events that result in endocytosis, effector secretion, septa formation and actin dynamics during the *A. rabiei* hyphal growth (Fig 10).

In a first set of evidences, our results indicate the role of membrane remodeling in septa formation and hyphal tip growth. Previous studies, largely conducted in animal models, have highlighted that the F-BAR domain is a membrane-deforming module and is involved in endocytosis [47,29]. The endocytic event is crucial for the uptake of signal cues and nutrition from the host, and aids apical recycling of membrane receptors and proteins. This process thus helps to maintain the overall polarity of the hyphae that is required for fungal growth and virulence [6,43]. The generation of EEs and their trafficking involves microtubule dynamics, actin cytoskeleton rearrangements and most importantly, extensive membrane remodeling [48]. Actin dynamics and microtubule organization have been extensively studied. However, the detailed mechanism underlying the functional regulation of fungal EE biogenesis and EE trafficking at the hyphal tip during plant pathogenesis remains poorly understood.

The present study identifies the role for the ArF-BAR protein as an essential component of endocytosis. Unveiling other networks associated with this system, ArF-BAR in coordination with Arp2/3-WASp, was found to mediate actin cytoskeleton assembly at the hyphal tip. The Arp2/3-WASp assembly is a prerequisite for host penetration [33, 49]. The distribution of the F-actin network, which serves as the molecular track for endosome trafficking [8,39], was disorganized in the Δ*arf-bar* mutant, affecting endocytic transport.

The loss of function mutant of the ArF-BAR function leads to the substantial attenuation of virulence compared to WT. This loss in virulence is similar to that observed in the rice blast fungus *M. oryzae* and *U. maydis* where endocytosis is crucial for the recognition of host partners during the early stages of pathogenic development [7]. However, the slow growth of the mutant (Fig 3 and S8 Fig) may be argued for a possible cause of the reduced virulence by itself. We observed that the symptoms in plant bioassay (Fig 2 and S6 Fig) are severe in case of WT. The

Δ*arf-bar* mutant showed only small or almost no lesions on plants. These observations clearly indicated that such severity not mere due to slow growth of hyphae. Therefore, we hypothesized that the knock-out mutant may have impaired in ability to secrete effectors of *A. rabiei*. Fungal pathogens secrete an array effector protein to counter host immunity and promote infection process [50]. Δ*arf-bar* mutant strain was found defective in secretion of a secretary signal sequence containing effector, Ar93 (Fig 5D). This experiment clearly shows that cumulative effect of both the slow growth and effector secretion are the primary cause of severe virulence defects in Δ*arf-bar* mutant. Additionally, some of the fungal effectors can change the structural and functional conformations as well as the localization of host target proteins that are key factors in host ROS production [51]. Pathogen survival under host-generated ROS is important for fungal growth and virulence. We also observed high transcript accumulation of under oxidative stress conditions (Fig 1B). However, it will be interesting to decipher the entire mechanism of pathogen survival correlating all the components together.

As discussed above, during host-pathogen interactions, the key to successful pathogenesis is to overcome the rigid defense responses of the host. Collectively, the first challenge the pathogen encounters is an oxidative burst at the site of infection, which initiates various signaling cascades in the pathogen that aid its survival. Calcium, an essential secondary messenger, mediates one such signaling cascade [52]. In response to stimuli, the cytosolic Ca^{2+} concentration increases [53] and modulates various Ca^{2+}-binding proteins such as calmodulin. The Ca^{2+} and calmodulin complex activates calcineurin. In many eukaryotes, calcineurin is known to regulate the activity of CRZ1, which is usually localized in the cytosol in phosphorylated form. Upon activation, CRZ1 gets relocated to the nucleus. In pathogenic fungi, many of the CRZ1-dependent targets,

such as those involved in the maintenance of cell wall integrity, thermo-tolerance, cation homeostasis, azole tolerance and hyphal growth have been identified [54]. Here, our study assumes functional significance for plant infection as we identified an F-BAR protein that is regulated by transcriptional network. We have identified the calcium-regulated CRZ1 transcription factor that regulates the transcription of F-BAR. These results, improves our understanding of the novel regulatory mechanism of endocytosis in filamentous fungi, where *ArF-BAR* functions downstream of ArCRZ1.

The development of septa is an important event during hyphal differentiation that is required for the formation of sexual structures and asexual spores [53]. Septation is comparable to cytokinesis that additionally includes cell separation. A cascade of events is involved in septum biogenesis, which includes assembly of the contractile actomyosin ring (CAR), plasma membrane ingression and cell wall constriction [55]. In fission yeast, Cdc15 and Imp2, and in budding yeast, Hof1 are the major F-BAR domain-containing proteins implicated in the formation of the CAR, and the primary and secondary septa during cytokinesis [56,57]. Additionally, Cdc15 of *U. maydis* also implicated for similar phenotype [16]. Therefore, the localization of ArF-BAR along the septum ring indicates that endocytosis is one of the pathways responsible for regulating the development of the septa. Septation is initiated at the definitive size of the hyphae [58]. With the initial recognition and establishment of disease in the host, the pathogen needs to proliferate at an enormously increased rate. Therefore, maintaining proper hyphal architecture and polarity is fundamental for pathogenesis that demands rapid coordinated internalization and recycling events [59]. The fungal mutant Δ*arf-bar*, deficient in ArF-BAR protein, revealed delay in septa formation and displayed decreased virulence in the absence of proper hyphal structure. In summary, we propose that the ArF-BAR protein of *A. rabiei* has the potential to interactively

affect the hyphal growth and virulence of filamentous fungi. This evolving model provides mechanistic insight into the role of a membrane scaffolding protein in the process of endosome trafficking in fungal pathogenesis. In turn, this provides many additional potential targets for the development of effective and durable strategies to control AB fungal disease. Therefore, the observations of the present study in context to the intracellular trafficking, during the early stages of plant-pathogen interactions, have broad relevance for shaping disease-control strategies. These findings may also be helpful for the disease control of animal-infecting fungal pathogens such as *A. fumigatus* and *C. albicans*. Thus, further studies will be directed to characterize the interacting partners influenced by ArF-BAR during endosome formation. Further, the understanding of its regulatory mechanism would help in scrutinizing the molecular and cellular basis of disease development, which would subsequently help develop disease-control strategies for filamentous fungi.

Materials and Methods

Fungal strains and growth conditions

A virulent isolate of *Ascochyta rabiei* (ArD2; ITCC No. 4638) was procured from IARI, New Delhi. The single spore culture of this mating type 2 isolate was generated and maintained as wild type fungi for research work. The WT and its derivative fungal strains (S2 Table) were maintained on potato dextrose agar (PDA; Difco Laboratories, pH 5.2-5.5) at 22ºC for 10-15 days to assess the growth pattern and colony characteristics [26]. The fungus was routinely subcultured on the PDA plates supplemented with chickpea extract to maintain virulence. To determine the vegetative growth pattern of fungal mycelia in response to oxidative stress, PDA plates supplemented with menadione (250 µM and 500 µM; Sigma-Aldrich, USA) and H_2O_2 (2 mM; Sigma-Aldrich, USA) were used. The conidial suspensions of 10 µl ($1x10^3$ conidia/mL)

were inoculated at the centre of PDA plates for growth assay. After 10 days of incubation under optimum growth conditions, the diameters of fungal colonies were measured using ImageJ software. Three independent biological experiments were performed with three technical replicates each time.

RNA extraction and expression analysis

Total RNA was extracted from 5-6 days old fungal mycelia grown in potato dextrose broth (PDB; Difco Laboratories, USA) or from plant tissues inoculated with *A. rabiei* wild-type (WT) using TRIzol reagent (Invitrogen, USA). The isolated total RNA was subjected to DNase1 (Promega, USA) treatment and subsequently used for first-strand cDNA synthesis using SuperScript IV reverse transcriptase (ThermoFisher Scientific). Targeted gene expression was determined by qRT-PCR with ABI7900 (Applied Biosystems, USA), using SYBR Green PCR master mix (Applied Biosystems, USA). Relative expression of fungal genes was calculated after normalization with elongation factor α (*ArEFα*; ST47_g4052) using this $2^{-\Delta\Delta ct}$ method [60]. The data were analysed from three biological replicates each having three technical replicates.

Site-directed mutagenesis

The mutations at required sites of *ArF-BAR* were achieved by PCR amplification of pET28a(+):*ArF-BAR* clone with pre-designed primers containing mutations of interest. Mutagenesis was performed using the QuikChange II Site-directed mutagenesis kit (Agilent, USA). Web-based Quik Change Primer Design tool (www.agilent.com/genomics/qcpd) was used to design primers. The presence of mutations in clones was confirmed by Sanger sequencing.

Targeted gene knockout and complementation in *A. rabiei*

Homologous gene replacement with *hph* cassette strategy was used to generate knockout (KO) constructs for *A. rabiei* genes. Genomic DNA was isolated from 5-day grown PDB culture of *A. rabiei* using GenElute Plant Genomic DNA miniprep kit (Sigma-Aldrich, USA). The 5′ flanking genomic sequences of *ArF-BAR* were amplified from *A. rabiei* genomic DNA using primer pairs of ArF-BARKOif5F and ArF-BARKOif5R while the 3′ flanking sequences were amplified using Ar72KO3 and Ar72KO4 (S3 Table). These amplified 5′ and 3′ flanking sequences were cloned sequentially into pGKO2 vector at *Kpn*I/*Pst*I and *Bam*HI/*Eco*RI sites, respectively. The cloned *ArF-BAR* gene replacement cassette of ~3.4 kb, including 5′ and 3′ flanking sequence along with *hph*, was amplified using primer pair ArF-BARKOif5F and Ar72KO4, and transformed into *A. rabiei* protoplasts. The *A. rabiei* protoplast transfection was performed as described earlier [61] , with some minor modifications. The putative transformants were selected on a PDA plate supplemented with 50 µg/µL hygromycin. To generate the complementation constructs of *ArF-BAR* and its mutated (mut1 and mut2) versions, about 4.4 kb DNA fragment having the *ArF-BAR* native promoter, ORF region, and TrpC terminator was amplified and cloned into pBIF2 vector (Bacterial selection- kanamycin; Fungal selection- G418) at *Eco*RI and *Hind*III sites. These three constructs of *ArF-BAR* were independently transformed into *Δarf-bar* mutant strain by *Agrobacterium tumefaciens*-mediated transformation (ATMT) [27]. Similar, to generation of ArF-BAR knockout and complementation mutant, *A. rabiei ArCRZ1* gene knockout mutant and complementation strains were also developed. The knockout primers used for the same enlisted in S3 Table.

Gene knockout confirmation by PCR and Southern blot

The single spore culture of putative KOs selected on hygromycin was initially screened by genomic PCR. A primer binding at position 5′ to the homologous recombination region and

TrpC promoter was used for gene upstream integrity check while a primer binding at *Hph* gene and position 3′ to the homologous recombination region was used for gene downstream integrity check (S3 Table). The putative transformants for complementation strains were also screened by genomic PCR. The PCR positive *Δarf-bar*, and *Δarcrz1* mutants (KOs) and complementation strains were further verified by Southern blot. The genomic DNA of WT, *Δarf-bar* and *Δarcrz1* mutants was digested with *Eco*RI enzyme while genomic DNA was digested with *Eco*RI and *Hind*III for the complementation strains (*Δarf-bar/ArF-BAR*, *Δarf-bar/ArF-BAR*^{mut1}, *Δarf-bar/ArF-BAR*^{mut2}, and *Δarcrz1/ArCRZ1*). The digested DNA was separated along with λ DNA/*Hind*III marker (ThermoFisher Scientific) and blotted to a membrane followed by hybridization with radioactive probe prepared using the random primers labeling NEBlot kit (New England Biolabs, USA). The band detection was carried out using Typhoon phosphor imager (GE Healthcare, USA).

Plant infection assay

Two-week-old susceptible chickpea (Pusa 362) plants grown in plant growth chambers under controlled conditions (D/N temperature: 24ºC/18ºC; Relative Humidity: 80%; light intensity 250 μE/m^2/s; D/N light duration: 14/10 h) were used for infection assays. Conidial suspensions of *A. rabiei* strains were collected separately from a 20-days-old PDA plate grown culture. Two-weeks-old plants were spray inoculated with conidial suspensions diluted to 2x10^{6}conidia/mL. Plants were again kept under optimum conditions. Disease lesions were examined 5-7 days after spray inoculation.

***In vitro* Protein purification**

Respective cDNAs were cloned in pET28a (+) and transformed into *E. coli* BL21-CodonPlus (DE3)-RIPL cells. Protein expression was induced with 0.5 mM Isopropyl β-D-thiogalactoside

(IPTG) for 6 h at 23ºC. Bacterial pellet was lysed in buffer [500 mMNaCl, 50 mM $NaPO_4$ (pH 8.0), 10 mM Imidazole, 1mM β-Mercaptoethanol, 1 mg/mL lysozyme and 1mM Phenylmethylsulfonyl fluoride (PMSF)] by incubating 30 min on ice, followed by sonication (10 cycle, 70% amplitute). The cell lysates were precipitated followed by 0.45 µm filtration. The cleared lysate was incubated with Ni-NTA resin for 30 min at 4ºC. The His-tagged fusion proteins were purified using a Ni-NTA column (Bio-Rad, USA). Proteins were eluted in elution buffer [500 mMNaCl, 50 mM $NaPO_4$ (pH 8.0), 10% glycerol and 200 mM imidazole]. The quality and quantity of eluted protein were checked by SDS/PAGE and Bradford assay, respectively. The proteins were dialyzed in respective compatible buffers followed by concentration.

Liposome preparation and Tubulation assay

Liposomes were prepared as described previously [63] using 70% Phosphatidylethanolamine (POPE), 20% Phosphatidylcholine (POPC), and 10% Rhodamine B-conjugated PE (Echelon Biosciences, USA). In an amber vials, all the lipids were initially dissolved in chloroform: methanol (65:35;v/v) mixture and vials were kept under liquid nitrogen for 10 min before being immediately subjected to vacuum desiccation/lyophilisation for 2 h at 60 mTorr. The lipids were hydrated with buffer [25mM Tris-HCl (pH 6.8) and 100mM NaCl] and subjected to three freeze-thaw cycles of 5 min each at 68 ºC and liquid nitrogen. Extrusion was performed at 68 ºC on a pre-heated mini extruder (Avanti Polar Lipids, USA). The prepared liposomes were diluted as desired and immediately proceeded for tubulation assay. Before use, the purified proteins were subjected to 100,000 g centrifugation for 20 min at 4 ºC to remove the aggregates. To examine tubule formation, the mixed liposome and protein samples were analysed in Lumox 24-well plate (Millipore, USA) by live-cell imaging on Axio Examiner.Z1 (Zeiss microscope).

Liposome co-sedimentation assay

The purified fusion proteins were pre-centrifuged, before the assay, at 100,000 g for 15 min to remove protein aggregates. Protein from the supernatant was mixed with freshly prepared synthetic liposomes with gentle tapping. Ultracentrifugation was performed at 100,000 g for 15 min at 4ºC and the supernatant and the pellet were carefully separated. The supernatant was mixed with 1:1 loading buffer while the pellet was re-suspended in 2x loading buffer. Samples were analysed on Coomassie stained SDS-PAGE.

Yeast two-hybrid assays

The interactions of various combinations between ArF-BAR, ArActin, ArWASP, and ArF-BAR domains in yeast cytoplasm were examined using the split-ubiquitin based DUALhunter system (Dualsystems Biotech). The ORFs were cloned at *Not*I and *Asc*I restriction sites or by LR clonase II into pGDHB1 and pGPR3-N vectors. The cloned plasmids were co-transformed along with necessary controls into NMY51 strain using the EZ- Yeast transformation kit (MP Biomedicals, USA) and plated on SD- L/- W plates. The plating of yeast clones on required synthetic media to check protein-protein interactions in yeast was done as described previously [22]. All the interactions were verified by three independent experiments.

Effector secretion assay

For total fungal protein extraction, fungal mycelia were grown in PDB at 22 ˚C, 120 rpm for 7 days and harvested by filtering through three layers of sterile Mira cloth (EMD Millipore Corp, Germany). Tissues were snap freezed in liquid Nitrogen and stored for future use. The mycelial tissue were grounded to fine powder and resuspended in Tris-glycine buffer pH 8.3 (3g Trizma, Sigma-Aldrich, USA and 14.4 g Glycine, Sigma-Aldrich, USA in1Lit MQ). Resuspension were then centrifuged at 16 x 1000g for 40 min at 4˚C. The supernatant or cell lysate were collected

and the total protein was separated on SDS PAGE followed by immune blot with anti-Flag Ab (Cohesion biosciences, Singapore). For the extraction of secreted proteins, 7 days old grown mycelia in PDB was treated with 250 μM menadione (Sigma-Aldrich, USA), to mimic host induced oxidative stress condition. Axenic culture filtrate (CF) was collected and filtered through three layered Mira cloth (EMD Millipore Corp, Germany) to separate fungal mycelia. Further, CF was sequentially filtered with 0.45 and 0.22 μm Durapore PVDF membrane filters (Sigma-Aldrich, USA). Filtered CF was then concentrated using 3 kDa Amicon Ultra-15 Centrifugal Filter Units (Merck, USA). Secreted proteins in concentrate were then separated on SDS-PAGE followed by immunoblot with anti-Flag Ab.

Actin polymerization assay

The actin polymerization modulation activity of the proteins was checked using Actin Polymerization Biochem Kit (Cytoskeleton, USA), using the manufacturer's instruction. Freshly solubilized components- 13 nM Arp2/3 protein complex and 15 nM WASp-VCA domain-GST purified (Cytoskeleton, USA) along with freshly purified ArF-BAR protein in concentrations of 200, 400 and 600 nM were used. The reaction was carried out in an opaque 96-well plate and kept in the dark. The actin polymerization rate was recorded by monitoring the pyrene fluorescence signals using CLARIO star plate reader (BMG Labtech, Germany) with the following settings; slow kinetics, 60 sec interval, λ_{ex} = 360±15 nm and λ_{em} = 420±20 nm.

Electrophoretic mobility shift assay (EMSA)

The His-tagged ArCRZ1 (*His-ArCRZ1*) expression construct was generated and recombinant protein was purified. The wild type and mutated CDRE DNA fragments were assembled by annealing oligonucleotide pairs in a thermal cycler by heating at 95° C for 5 min followed by cooling at RT for 15 min. End labelling of DNA fragments was performed by [γ^{32}P] dATP

(Board of Radiation & Isotope Technologies, BRIT, India), and Polynucleotide Kinase (Thermo Fisher Scientific, USA). Additionally, for competition assays, two complementary oligonucleotides were annealed at equimolar concentration. Purified His-ArCRZ1 protein was incubated with 10 ng of labelled DNA fragment in the presence of 1 mg of poly-deoxy-inosinic-deoxy-cytidylic acid [poly (dI-dC)] and 1X binding buffer (15mM HEPES (pH 7.6), 0.2 mM $MgCl_2$, 35 mM KCl, 1 mM DTT and 1% glycerol) in a reaction volume of 30 µL for 25 min at room temperature. DNA loading dye was used to terminate the reaction. The competitive assays were performed using 50, 100 and 200 times of specific fragments in excess. To identify the relative binding, the complexes were resolved on 6% native PAGE, dried, and autoradiographed on X-ray films.

Microscopic methods

Sample preparation for microscopic analysis

The conidia from the respective fungal strains were harvested in 1 mL sterile distilled water from 15-day-old fungal mycelia grown on PDA plates. The conidial suspension was filtered through Mira cloth. Ten microlitres (1×10^6 conidia/mL) of suspension was kept on a sterile glass coverslip and allowed to grow under the optimal conditions for 24 h in dark. To investigate the localization of chimeric proteins in fungal hyphae, growing on chickpea stem peel, the conidia were allowed to grow for 36 h under optimum conditions of infection. The grown hyphae samples were then used for confocal laser scanning microscopy.

Confocal laser scanning microscopy

For microscopic studies, TCS SP5 and TCS SP8 confocal laser scanning microscopes (Leica Microsystems, Germany) were used. For subcellular localization, conidia were harvested from transgenic fungal strains expressing fluorescent-tagged proteins. The Z-stacked images with 1

µm step size were acquired using a high-resolution CCD camera. For calcofluor-white (CW), the hyphae grown on the glass slide were incubated for 10 min in the CW solution (Sigma-Aldrich, USA). After incubation, the stained hyphae were rinsed with PBS (pH 7.4) followed by rinsing with sterile distilled water, before image acquisition. For FM4-64 uptake, 1 mL of harvested conidial suspension was centrifuged at 2,500 g, washed twice with sterile distilled water and then allowed to germinate on glass slides. The aqueous solution (10 µM) of FM4-64 dye (Invitrogen, USA) was added directly to the fungal mycelia. After 10 min incubation, FM4-64 dye was rinsed from slides thoroughly before imaging fungal hyphae. Images were captured with a TCS SP8.

For host penetration assay, infected chickpea leaves (WT and Δ*arf-bar*) were placed in 100 % ethanol for 48 h to undergo bleaching for the complete removal of chlorophyll. Subsequently, leaves were incubated for 4 h in 10 % KOH at RT followed by washing 4-5 times in Phosphate buffer saline (PBS, pH 7.4). The processed leaves were then stained with chitin specific dye WGA-AF 488 (Invitrogen, USA). The leaf samples were rinsed in PBS (pH 7.5) before microscopic visualization. Confocal images were captured with aa TCS SP5 confocal microscope.

For sub-cellular localization of ArCRZ1::EYFP under oxidative stress conditions, fungal conidia expressing chimeric protein were isolated and allowed to grow for 12 h. The hyphae were exposed to $CaCl_2$ (200 mM, Sigma-Aldrich, USA) for 1 min prior to microscopy. To assess the involvement of calcineurin in nuclear localization of ArCRZ1::EYFP, the hyphae were exposed to 5 µg/µL FK506 (Sigma-Aldrich, USA), 5 min prior to the addition of $CaCl_{2.}$ The confocal images were acquired with a TCS SP5.

For subcellular localization of ArCRZ1::EYFP during infection, susceptible plants were challenged with conidia expressing ArCRZ1::EYFP. The images were acquired after 48 h using TCS SP5.

Quantification and Statistical analysis

Quantification analysis of relative fluorescent intensity, lesion size, radial diameter, the distance of septa from the hyphal tip were analysed by ImageJ/Fiji software. To calculate the significance of means/differences between two groups, Student's t-test and oneway ANOVA followed by Tukey test between multiple groups were performed using GraphPad Prism 6. Significance was accepted at $p < 0.05$, as noted in the text of legends. Replicates are indicated in the legends.

Bioinformatic analysis

All the gene and protein sequences were acquired from NCBI server (http://www.ncbi.nlm.nih.gov). Multiple sequence alignment was performed by Praline search (http://www.ibi.vu.nl/programs/pralinewww/). Phylogenetic analysis was performed with MEGA7.0.21 software. Distinctive domain organization of the protein was determined by SMART search (http://smart.embl-heidelberg.de/). The putative TFs binding sites were identified by YEASTRACT-DISCOVERER Database (http;//yeastract.com). The theoretical pI and molecular weight of the chimeric proteins were determined by Expasy compute pI/Mw tool (http://web.expasy.org/cgi-bin/compute_pi/pi_tool). Protein IDs of protein sequences used in this study are: ArF-BAR: KZM20872.1, ArRab5: KZM19760.1, ArRab7: KZM26450.1, ArActin: KZM21342.1, ArWASP: KZM19192.1, ArCRZ1: KZM25117.1 and ArSNC1: KZM18806.1.

Acknowledgements

We gratefully acknowledge a research grant from Department of Biotechnology, Government of India (File No: BT/PR10605/PBD/16/791/2008 and BT/AGR/CG-Phase II/01/2014) and a core grant from National Institute of Plant Genome Research, New Delhi, India for funding this work. MS, AS, and KS acknowledge Council of Scientific and Industrial Research and University Grants Commission, Government of India for their fellowships.

Author contributions

PKV and VK designed the project; MS, AS, KS and SKS carried out experiments; PKV, KK and KS supervised the work; all authors analysed and discussed the data; MS, AS, KK and VK wrote the manuscript; all the authors reviewed and approved the manuscript; MS and AS contributed equally to this work.

References

1. Takeshita N, Evangelinos M, Zhou L, Serizawa T, Somera-Fajardo RA, Lu L, et al. Pulses of Ca^{2+} coordinate actin assembly and exocytosis for stepwise cell extension. Proc Natl Acad Sci U S A. 2017;114: 5701–5706. doi:10.1073/pnas.1700204114

2. Riquelme M, Aguirre J, Bartnicki-García S, Braus GH, Feldbrügge M, Fleig U, et al. Fungal Morphogenesis, from the Polarized Growth of Hyphae to Complex Reproduction and Infection Structures. Microbiol Mol Biol Rev. 2018. doi:10.1128/mmbr.00068-17

3. Etxebeste O, Espeso EA. Neurons show the path: Tip-to-nucleus communication in filamentous fungal development and pathogenesisa. FEMS Microbiology Reviews. 2016. doi:10.1093/femsre/fuw021

4. Bielska E, Higuchi Y, Schuster M, Steinberg N, Kilaru S, Talbot NJ, et al. Long-distance

endosome trafficking drives fungal effector production during plant infection. Nat Commun. 2014. doi:10.1038/ncomms6097

5. Higuchi Y, Arioka M, Kitamoto K. Endocytic recycling at the tip region in the filamentous fungus *Aspergillus oryzae*. Commun Integr Biol. 2009. doi:10.4161/cib.2.4.8385

6. Higuchi Y, Shoji JY, Arioka M, Kitamoto K. Endocytosis is crucial for cell polarity and apical membrane recycling in the filamentous fungus *Aspergillus oryzae*. Eukaryot Cell. 2009;8: 37–46. doi:10.1128/EC.00207-08

7. Takeshita N. Coordinated process of polarized growth in filamentous fungi. Biosci Biotechnol Biochem. 2016;80: 1693–1699. doi:10.1080/09168451.2016.1179092

8. Takeshita N, Higashitsuji Y, Konzack S, Fischer R. Apical sterol-rich membranes are essential for localizing cell end markers that determine growth directionality in the filamentous fungus *Aspergillus nidulans*. Mol Biol Cell. 2008. doi:10.1091/mbc.E07-06-0523

9. Rao Y, Rückert C, Saenger W, Haucke V. The early steps of endocytosis: From cargo selection to membrane deformation. European Journal of Cell Biology. 2012. doi:10.1016/j.ejcb.2011.02.004

10. Frost A, Perera R, Roux A, Spasov K, Destaing O, Egelman EH, et al. Structural Basis of Membrane Invagination by F-BAR Domains. Cell. 2008. doi:10.1016/j.cell.2007.12.041

11. Habermann B. The BAR-domain family of proteins: a case of bending and binding? The membrane bending and GTPase-binding functions of proteins from the BAR-domain family. EMBO Rep. 2004. doi:10.1038/sj.embor.7400105

12. Krauss M, Haucke V. A novel twist in membrane dephormation. Developmental Cell. 2014. doi:10.1016/j.devcel.2014.09.016

13. Zimmerberg J, Kozlov MM. How proteins produce cellular membrane curvature. Nature Reviews Molecular Cell Biology. 2006. doi:10.1038/nrm1784

14. Saarikangas J, Zhao H, Pykäläinen A, Laurinmäki P, Mattila PK, Kinnunen PKJ, et al. Molecular Mechanisms of Membrane Deformation by I-BAR Domain Proteins. Curr Biol. 2009. doi:10.1016/j.cub.2008.12.029

15. Fuchs U, Hause G, Schuchardt I, Steinberg G. Endocytosis is essential for pathogenic development in the corn smut fungus *Ustilago maydis*. Plant Cell. 2006. doi:10.1105/tpc.105.039388

16. Böhmer C, Ripp C, Bölker M. The germinal centre kinase Don3 triggers the dynamic rearrangement of higher-order septin structures during cytokinesis in *Ustilago maydis*. Mol Microbiol. 2009. doi:10.1111/j.1365-2958.2009.06948.x

17. Lin L, Chen X, Shabbir A, Chen S, Chen X, Wang Z, et al. A putative N-BAR-domain protein is crucially required for the development of hyphae tip appressorium-like structure and its plant infection in *Magnaporthe oryzae*. Phytopathol Res. 2019;1: 1–15. doi:10.1186/s42483-019-0038-2

18. Dagdas YF, Yoshino K, Dagdas G, Ryder LS, Bielska E, Steinberg G, et al. Septin-mediated plant cell invasion by the rice blast fungus, *Magnaporthe oryzae*. Science (80-). 2012;336: 1590–1595. doi:10.1126/science.1222934

19. Soulard A, Lechler T, Spiridonov V, Shevchenko A, Shevchenko A, Li R, et al. Saccharomyces cerevisiae Bzz1p Is Implicated with Type I Myosins in Actin Patch

Polarization and Is Able To Recruit Actin-Polymerizing Machinery In Vitro. Mol Cell Biol. 2002. doi:10.1128/mcb.22.22.7889-7906.2002

20. Fricke R, Gohl C, Dharmalingam E, Grevelhörster A, Zahedi B, Harden N, et al. Drosophila Cip4/Toca-1 Integrates Membrane Trafficking and Actin Dynamics through WASP and SCAR/WAVE. Curr Biol. 2009. doi:10.1016/j.cub.2009.07.058

21. Arasada R, Pollard TD. Distinct roles for F-BAR proteins Cdc15p and Bzz1p in actin polymerization at sites of endocytosis in fission yeast. Curr Biol. 2011. doi:10.1016/j.cub.2011.07.046

22. Kumar K, Purayannur S, Kaladhar VC, Parida SK, Verma PK. mQTL-seq and classical mapping implicates the role of an AT-HOOK MOTIF CONTAINING NUCLEAR LOCALIZED (AHL) family gene in *Ascochyta* blight resistance of chickpea. Plant Cell Environ. 2018;41: 2128–2140. doi:10.1111/pce.13177

23. Pandey BK, Singh US, Chaube HS. Mode of Infection of *Ascochyta* Blight of Chickpea Caused by *Ascochyta rabiei*. J Phytopathol. 1987. doi:10.1111/j.1439-0434.1987.tb04387.x

24. Ilarslan H, Dolar FS. Histological and ultrastructural changes in leaves and stems of resistant and susceptible chickpea cultivars to *Ascochyta rabiei*. J Phytopathol. 2002. doi:10.1046/j.1439-0434.2002.00763.x

25. Verma S, Gazara RK, Nizam S, Parween S, Chattopadhyay D, Verma PK. Draft genome sequencing and secretome analysis of fungal phytopathogen *Ascochyta rabiei* provides insight into the necrotrophic effector repertoire. Sci Rep. 2016. doi:10.1038/srep24638

26. Singh K, Nizam S, Sinha M, Verma PK. Comparative transcriptome analysis of the

necrotrophic fungus *Ascochyta rabiei* during oxidative stress: Insight for fungal survival in the host plant. PLoS One. 2012. doi:10.1371/journal.pone.0033128

27. Nizam S, Singh K, Verma PK. Expression of the fluorescent proteins DsRed and EGFP to visualize early events of colonization of the chickpea blight fungus *Ascochyta rabiei*. Curr Genet. 2010;56: 391–399. doi:10.1007/s00294-010-0305-3

28. Tsujita K, Suetsugu S, Sasaki N, Furutani M, Oikawa T, Takenawa T. Coordination between the actin cytoskeleton and membrane deformation by a novel membrane tubulation domain of PCH proteins is involved in endocytosis. J Cell Biol. 2006. doi:10.1083/jcb.200508091

29. Kumar V, Fricke R, Bhar D, Reddy-Alla S, Krishnan KS, Bogdan S, et al. Syndapin promotes formation of a postsynaptic membrane system in *Drosophila*. Mol Biol Cell. 2009. doi:10.1091/mbc.E08-10-1072

30. Heung LJ, Luberto C, Plowden A, Hannun YA, Del Poeta M. The sphingolipid pathway regulates Pkc1 through the formation of diacylglycerol in *Cryptococcus neoformans*. J Biol Chem. 2004. doi:10.1074/jbc.M312995200

31. Lanver D, Mendoza-Mendoza A, Brachmann A, Kahmann R. Sho1 and Msb2-related proteins regulate appressorium development in the smut fungus *Ustilago maydis*. Plant Cell. 2010. doi:10.1105/tpc.109.073734

32. Valkonen M, Kalkman ER, Saloheimo M, Penttilä M, Read ND, Duncan RR. Spatially segregated SNARE protein interactions in living fungal cells. J Biol Chem. 2007. doi:10.1074/jbc.M700916200

33. Li YB, Xu R, Liu C, Shen N, Han LB, Tang D. *Magnaporthe oryzae* fimbrin organizes

actin networks in the hyphal tip during polar growth and pathogenesis. PLoS Pathog. 2020;16: 1–27. doi:10.1371/journal.ppat.1008437

34. Giraldo MC, Dagdas YF, Gupta YK, Mentlak TA, Yi M, Martinez-Rocha AL, et al. Two distinct secretion systems facilitate tissue invasion by the rice blast fungus *Magnaporthe oryzae*. Nat Commun. 2013. doi:10.1038/ncomms2996

35. Qualmann B, Koch D, Kessels MM. Let's go bananas: Revisiting the endocytic BAR code. EMBO Journal. 2011. doi:10.1038/emboj.2011.266

36. Fischer-Parton S, Parton RM, Hickey PC, Dijksterhuis J, Atkinson HA, Read ND. Confocal microscopy of FM4-64 as a tool for analysing endocytosis and vesicle trafficking in living fungal hyphae. J Microsc. 2000;198: 246–259. doi:10.1046/j.1365-2818.2000.00708.x

37. Zeigerer A, Gilleron J, Bogorad RL, Marsico G, Nonaka H, Seifert S, et al. Rab5 is necessary for the biogenesis of the endolysosomal system in vivo. Nature. 2012. doi:10.1038/nature11133

38. Steinberg G. Endocytosis and early endosome motility in filamentous fungi. Curr Opin Microbiol. 2014;20: 10–18. doi:10.1016/j.mib.2014.04.001

39. Almeida-Souza L, Frank RAW, García-Nafría J, Colussi A, Gunawardana N, Johnson CM, et al. A Flat BAR Protein Promotes Actin Polymerization at the Base of Clathrin-Coated Pits. Cell. 2018. doi:10.1016/j.cell.2018.05.020

40. Berepiki A, Lichius A, Read ND. Actin organization and dynamics in filamentous fungi. Nat Rev Microbiol. 2011;9: 876–887. doi:10.1038/nrmicro2666

41. Riedl J, Crevenna AH, Kessenbrock K, Yu JH, Neukirchen D, Bista M, et al. Lifeact: A

versatile marker to visualize F-actin. Nat Methods. 2008. doi:10.1038/nmeth.1220

42. Monteiro PT, Oliveira J, Pais P, Antunes M, Palma M, Cavalheiro M, et al. YEASTRACT+: A portal for cross-species comparative genomics of transcription regulation in yeasts. Nucleic Acids Res. 2020. doi:10.1093/nar/gkz859

43. Park HS, Lee SC, Cardenas ME, Heitman J. Calcium-Calmodulin-Calcineurin Signaling: A Globally Conserved Virulence Cascade in Eukaryotic Microbial Pathogens. Cell Host Microbe. 2019;26: 453–462. doi:10.1016/j.chom.2019.08.004

44. Hernández-Ortiz P, Espeso EA. Spatiotemporal dynamics of the calcineurin target CrzA. Cell Signal. 2017. doi:10.1016/j.cellsig.2016.11.005

45. Yoshimoto H, Saltsman K, Gasch AP, Li HX, Ogawa N, Botstein D, et al. . Genome-wide analysis of gene expression regulated by the calcineurin/Crz1p signalingpathway in *Saccharomyces cerevisiae*. J Biol Chem. 2002.doi:https://doi.org/10.1074/jbc.M202718200

46. Li X, Gao C, Li L, Liu M, Yin Z, Zhang H, et al. MoEnd3 regulates appressorium formation and virulence through mediating endocytosis in rice blast fungus *Magnaporthe oryzae*. PLoS Pathog. 2017. doi:10.1371/journal.ppat.1006449

47. Leibfried A, Fricke R, Morgan MJ, Bogdan S, Bellaiche Y. Drosophila Cip4 and WASp Define a Branch of the Cdc42-Par6-aPKC Pathway Regulating E-Cadherin Endocytosis. Curr Biol. 2008. doi:10.1016/j.cub.2008.09.063

48. Grove SN, Bracker CE. Protoplasmic organization of hyphal tips among fungi: vesicles and Spitzenkörper. J Bacteriol. 1970. doi:10.1128/jb.104.2.989-1009.1970

49. Peñalva MÁ. Endocytosis in filamentous fungi: Cinderella gets her reward. Curr Opin

Microbiol. 2010;13: 684–692. doi:10.1016/j.mib.2010.09.005

50. Lo Presti L, Lanver D, Schweizer G, Tanaka S, Liang L, Tollot M, et al. Fungal effectors and plant susceptibility. Annu Rev Plant Biol. 2015. doi:10.1146/annurev-arplant-043014-114623

51. Singh Y, Nair AM, Verma PK. Surviving the odds: from perception to survival of fungal phytopathogens under host-generated oxidative burst. Plant Communications. 2021. doi:10.1016/j.xplc.2021.100142

52. de Castro PA, Colabardini AC, Manfiolli AO, Chiaratto J, Silva LP, Mattos EC, et al. *Aspergillus fumigatus* calcium-responsive transcription factors regulate cell wall architecture promoting stress tolerance, virulence and caspofungin resistance. PLoS Genetics. 2019. doi:10.1371/journal.pgen.1008551

53. Harris SD. Septum formation in *Aspergillus nidulans*. Curr Opin Microbiol. 2001;4: 736–739. doi:10.1016/S1369-5274(01)00276-4

54. Frost A, Unger VM, De Camilli P. The BAR Domain Superfamily: Membrane-Molding Macromolecules. Cell. 2009. doi:10.1016/j.cell.2009.04.010

55. Delgado-Álvarez DL, Bartnicki-García S, Seiler S, Mouriño-Pérez RR. Septum development in *Neurospora crassa*: The septal actomyosin tangle. PLoS One. 2014;9: 29–30. doi:10.1371/journal.pone.0096744

56. Guertin DA, Trautmann S, McCollum D. (CUL-ID:2436029) Cytokinesis in Eukaryotes. Microbiol Mol Biol Rev. 2002. doi:10.1128/MMBR.66.2.155-178.2002

57. Wu JQ, Kuhn JR, Kovar DR, Pollard TD. Spatial and temporal pathway for assembly and constriction of the contractile ring in fission yeast cytokinesis. Dev Cell. 2003.

doi:10.1016/S1534-5807(03)00324-1

58. Martín-García R, Arribas V, Coll PM, Pinar M, Viana RA, Rincón SA, et al. Paxillin-Mediated Recruitment of Calcineurin to the Contractile Ring Is Required for the Correct Progression of Cytokinesis in Fission Yeast. Cell Rep. 2018. doi:10.1016/j.celrep.2018.09.062

59. Lamb CJ, Lawton MA, Dron M, Dixon RA. Signals and transduction mechanisms for activation of plant defenses against microbial attack. Cell. 1989. doi:10.1016/0092-8674(89)90894-5

60. Livak KJ, Schmittgen TD. Analysis of relative gene expression data using real-time quantitative PCR and the $2^{-\Delta\Delta CT}$ method. Methods. 2001. doi:10.1006/meth.2001.1262

61. Talbot NJ, Ebbole DJ, Hamer JE. Identification and characterization of MPG1, a gene involved in pathogenicity from the rice blast fungus *Magnaporthe grisea*. Plant Cell. 1993. doi:10.1105/tpc.5.11.1575

MAIN FIGURE LEGENDS

Fig 1. ArF-BAR is a bonafide stress induced membrane tubulating protein.

Schematic representation of domain organization of ArF-BAR protein. It has a F-BAR domain (amino acids 7–253), a C1 domain of Protein Kinase C1, PKC1 (amino acids 407–456) and two consecutive SH3 domains (SH3a amino acids 572–651 and SH3b amino acids 705–760). (B) Relative fold change in the *ArF-BAR* transcript level of 5 days axenically grown potato dextrose broth mycelium culture of *Ascochyta rabiei* post 250 µM menadione treatment. The treated mycelia samples were collected every 0.5 h and the expression was analysed with qRT-PCR. (C) Relative fold change in the transcript level of *ArF-BAR* post *A. rabiei* conidial suspension inoculation. The conidia inoculated chickpea aerial tissue was collected up to 72 hpi and expression was analysed by qRT-PCR. (D-G) Membrane deforming activity of ArF-BAR. The bar represents 20 µm. (D) The liposomes remain spherical in presence of protein resuspension buffer, acting as control. (E, G) Tubulation of synthetic liposomes uponaddition of the recombinant ArF-BAR and ArF-BAR^{mut2} proteins. (F) Liposome tubulation activity negligible in ArF-BAR^{mut1}. (H) The bar chart shows the percentage of the tubule formed by liposomes in presence of native and mutated recombinant proteins. Out of 150 liposomes, the percentage of the tubule formation was counted in each set of experiment. (I) Liposome co-sedimentation assay, where the ArF-BAR and ArF-BAR^{mut2} recombinant protein bound with lipid membranes (liposome) were found in pellet, while membrane binding activity is lost in ArF-BAR^{mut1} protein. Statistical analysis was performed using Student's t-test one tailed compared with its control. Significant differences are indicated as $*p < 0.03$; $**p < 0.003$; $***p < 0.0005$; $****p < 0.0001$; ns = non-significant. Data is the mean of three independent biological replicates with error bars ± representing standard deviation. Red dots represent the average value of all technical replicate in

each biological replicate used for the quantitative analysis.

Fig 2. ArF-BAR is required for full virulence of *A. rabiei* in chickpea.

(A) To examine radial vegetative growth on PDA plates, *A. rabiei* (WT), Δ*arf-bar* and Δ*arf-bar/ArF-BAR* mutant strains were grown for 7 days at 22ºC. (B) Representative image of ascochyta blight (AB) disease symptoms on susceptible chickpea plants at 6 dpi, obtained after inoculation with conidial suspension of *A. rabiei* (WT) and mutant strains. Decreased AB symptoms in Δ*arf-bar and* Δ*arf-bar/ArF-BAR*^{mut1} shows reduced virulence. (C) Statistical values of the number of lesions per plant after conidial inoculation of chickpea with *A. rabiei* (WT), mutant and mutant complemented strains. The graph represents the mean and standard deviation of three independent biological replicates, counting at least 20 plants in each replicate. (The bars in graph correspond to- 14.99 ± 0.84 in WT; 2.66 ± 0.19 in Δ*arf-bar*; 12.86 ± 0.69 in Δ*arf-bar/ArF-BAR*; 3.02 ± 0.74 in Δ*arf-bar/ArF-BAR*^{mut1}and 8.833 ± 0.04 in Δ*arf-bar/ArF-BAR*^{mut2}). (D) Statistical values of the lesions' diameter was calculated (The bar in graphs corresponds to; WT = 3.67 mm ± 0.46; Δ*arf-bar* = 1.07 mm ± 0.14; Δ*arf-bar/ArF-BAR* = 3.84 ± 0.54; Δ*arf-bar/ArF-BAR*^{mut1} = 1.33 ± 0.06 and Δ*arf-bar/ArF-BAR*^{mut2} = 2.97 ± 0.15). Statistical analysis was performed using ordinary one-way ANOVA compared with its control (****$p < 0.0001$; ***$p < 0.001$; *$p < 0.05$). Red dot represent the average value of all technical replicate in each biological replicate used for the quantitative analysis.

Fig 3. ArF-BAR is required for vegetative growth and oxidative stress tolerance.

(A) Colony morphology and growth assay of *A. rabiei* (WT), Δ*arf-bar* mutant and mutant complemented strains, observed 10 days after incubation at 22ºC. For oxidative stress, the PDA

was supplemented with 250 µM and 500 µM menadione and 2mM H_2O_2. Strains of Δ*arf-bar* and Δ*arf-bar/ArF-BAR*^{mut1} exhibited more sensitivity towards oxidative stress condition as compared to WT *A. rabiei*. (B) The bar of graph represents mean and SD values of fungal strains radial diameter in the control PDA plates and supplemented PDA. All the PDA growth assays were performed in triplicate. The results were quantified using two-way ANOVA, Tukey's multiple comparisons. ****$p < 0.0001$, ***$p < 0.001$, **$p < 0.01$, *$p < 0.05$, ns = non-significant. Red dots represent the value of each biological replicate used for the quantitative analysis.

Fig 4. ArF-BAR primarily decorates the growing hyphal tip.

(A) Ectopically expressed ArF-BAR::EYFP and ArF-BAR^{mut2}::EYFP chimeric proteins are distributed at the hyphal tip. Hyphae expressing ArF-BAR^{mut1}::EYFP, was diffused throughout the fungal hyphae. The representative images were captured at 12 h post germination of fungal conidia on microscopic coverslip. Scale bar = 5 µm. (B) ArF-BAR::EYFP signal is uniformly distributed at fungal septum, scale bar = 5 µm. Star represents the hyphal septa. (C) The growing hyphal tip localization of ArF-BAR::EYFP was examined along with *A. rabiei* Spitzenkörper marker, ArSNC1::mCherry. Arrowhead represents the localization of ArSNC1. The v-SNARE SNC1 along with other proteins mediate docking and fusion of vesicles with the plasma membrane target site. Micrographs shows representative image of three biological replicates with at least 10 images in each replicate.

Fig 5. Δ*arf-bar* perturbs septa formation, host penetration and effector secretion.

(A) 3D structure stacking z-slices together shows the penetration ability of *A. rabiei* (WT) and Δ*arf-bar* on the chickpea host leaves. The Δ*arf-bar* mutant could penetrate on an average *Z* value

of 10 µm as compared to WT value of 20 µm. (B) Calcofluor white stained *A. rabiei* (WT) and Δ*arf-bar* hyphae after 12 h post germination. Increase in the distance of first septa from the polarised end was found in Δ*arf-bar*. Black line marks the position of first septa from growing hyphal tip in both WT and Δ*arf-bar*. Scale bar = 10 µm. (C) Bars in graph represent the mean values with SEM, of first cell and second cell length (in µm) from the growing tip end. Significance of the difference in length of cells was calculated using one-tailed paired t-test, (****p < 0.001). Red dots represent the value of each biological replicate used for the quantitative analysis.(D) Secretion of an ectopically expressed candidate secretory effector possessing a classical signal peptide, Ar93::EYFP::Flag (43 kDa) in WT and Δ*arf-bar* mutant. The Δ*arf-bar* strain of *A. rabiei* is impaired in effector secretion in contrast to WT strain. The absence of signal in the western blot in Δ*arf-bar* mutant represents absence of secreted Ar93 effector in culture filtrate, although the chimeric Ar93 is present in the cell lysate of both strains. The minus (–) sign represents Ar93 non-transformed and plus (+) represents Ar93:EYFP:FLAG transformed *A. rabiei* strains. Ponceau S stained blots represent as loading control.

Fig 6. ArF-BAR facilitate endocytosis.

(A) Confocal images of *A. rabiei* (WT) and Δ*arf-bar* hyphae after 10 min incubation with FM 4-64 to acquire internalization capacity. Scale bar = 5 µm. Right panel represents the difference in fluorescence intensity per unit area of FM 4-64 in *A. rabiei* (WT) and Δ*arf-bar* hyphae after internalization of the dye. The mean fluorescence intensity was statistically examined using one-tailed paired t-test, ***p < 0.0002. Red dots represent the value of each biological replicate used for the quantitative analysis. (B) Confocal images showing co-localization of ArF-BAR::mCherry with EGFP::ArRab5 (n = 22), scale bar = 10 µm. Right panel shows the positive

correlation between the fluorescence intensity of ArF-BAR (Red) with ArRAB5 (Green) across the magenta line in merged panel. (C) ArF-BAR does not localizes with the late endosome marker ArRab7::EYFP. The representative image is the maximum intensity projection of all *z*-stack images with 1 µm step size. Images were acquired after 12 h post-germination of fungal conidia expressing ArF-BAR::mCherry and ArRab7::EYFP. Scale bar = 5 µm, (n= 5).

Fig 7. ArF-BAR modulates the actin cytoskeleton.

(A) Split-ubiquitin based yeast two-hybrid assay to check protein-protein interaction in yeast cytoplasm. The lack of growth for yeast colonies on interaction selective QDO (SD/-L/-W/-A/-H) media as compared to vector selective DDO (SD/-L/-W) suggest no interaction between ArF-BAR and ArActin. The LargeT and Δp53 are interaction positive controls while pAI-Alg5 and pDL2-Alg5 are the controls to check proper bait expression and autoactivation, respectively. (B) ArF-BAR exhibits positive interaction with ArWASp through its SH3 domains [$SH3_{(ArF\text{-}BAR)}$; 508-760 amino acids]. The representative images were photographed 48 h after spotting 10 µl yeast cells suspension. The results were evaluated with three independent biological replicates. (C) Kinetics of WASp and Arp2/3 mediated actin polymerization in absence and presence of ArF-BAR. The kinetics was measured with the change in fluorescence of pyrene-actin. For all *in vitro* actin polymerization assays; 4 µM pyrene labelled actin, 13 nM Arp2/3 and 15 nM WASp were used. All the experiments were performed in three replicates. (D) Fluorescence image of Lifeact::mCherry expressed in fungal hyphae WT and Δ*arf-bar*, where discrete actin patches and cables are visible in WT however actin patches are sparsely visible in Δ*arf-bar*. Scale bar = 5 µm (n = 10). (E) Statistical analysis of the number of actin patches, visualized by Lifeact::mCherry

fluorescence, in WT and Δ*arf-bar* strain hyphae. n = 10. Red dots represents the total number of actin patches present in each biological replicate used for quantitative analysis.

Fig 8. ArCRZ1 binds *ArF-BAR* promoter and localizes to nucleus under stress conditions. (A) Schematic representation of ArCRZ1 TF binding sites at the 5′ regulatory region of *ArF-BAR* gene, identified by YEASTRACT online tool in 727 bp sequence. (B) Schematic representation of 58 bp WT and mutated Calcium Dependent Response Element (CDRE) probes used for electrophoretic mobility shift assay (EMSA) of recombinant ArCRZ1. Mutated nucleotides are depicted in red colour. (C) The EMSA blot of recombinant ArCRZ1 with WT or mutated CDRE dsDNA probes. His-purified ArCRZ1 recombinant protein in 500, 600 and 200 ng quantities was used in lane 2, 3 and 4, respectively. The same protein in 800, 200 and 100 ng amount was used in lane 5, 6 and 7, respectively. The lane 1 and 8 contains only the dsDNA probe with protein. The plus (+) and minus sign (-) representation for labelled probes show the presence and absence of dsDNA probes. The lane 9 has WT CDRE, and lane 10, 11 and 12 have mutated CDRE. This EMSA experiment was performed in triplicate. (D) Confocal images showing the cytosolic distribution of ArCRZ1::EYFP in the absence of stress condition and nuclear localization of ArCRZ1::EYFP in hyphae under stress condition (0.2 M $CaCl_2$). Overnight grown fungal hyphae was exposed to 0.2 M $CaCl_2$ two minutes prior to the microscopy. DAPI fluorescence was simultaneously recorded. Right panel in the confocal images shows the cytosolic distribution of ArCRZ1::EYFP under stress condition (200 mM $CaCl_2$) in the presence of FK506 (5 µg/µl) calcineurin inhibition. Fungal hyphae were exposed to FK506 for 5 min, prior to microscopy. Scale bar = 5 µm. (E) Confocal images showing the nuclear localization of ArCRZ1, during *in planta* fungal growth. Confocal images were acquired

48 hpi of AB susceptible chickpea stem peel, infected with conidia of fungal strain expressing ArCRZ1::EYFP. Scale bar = 5 µm (n= 30).

Fig 9. *ArCRZ1* regulates *ArF-BAR* transcription and regulates fungal virulence.

(A) Vegetative growth phenotype of 7 days old *A. rabiei* (WT), Δ*arcrz1* and Δ*arcrz1/ArCRZ1* strains grown on PDA plate. (B) Expression analysis of *ArF-BAR* gene in Δ*arcrz1* through semi-quantitative PCR. Lane 1 and 2 shows *ArF-BAR* expression in mycelia of *A. rabiei* (WT) and Δ*arcrz1* while lane 3 and 4 shows *ArF-BAR* expression in *A. rabiei* (WT) and Δ*arcrz1* after 0.5 h treatment with 250 µM menadione to the axenic culture of mycelia in PDB. (C) Representative image of disease symptoms obtained on *Ascochyta* blight susceptible chickpea 6 days post inoculation (dpi) of *A. rabiei* (WT), Δ*arcrz1* and Δ*arcrz1/ArCRZ1* strains. (D) Statistical analysis of the AB disease symptoms on chickpea plants inoculated with three different *A. rabiei* strains. The height of bar in graph shows the mean value of lesions per plant (WT = 15.31 ± 1.44, Δ*arcrz1* = 2.32 ± 0.80 and Δ*arcrz1/ArCRZ1* = 14.82 ± 0.73). (E) The bar height in graph represents the mean value of lesion size, in diameter (WT = 4.04 mm ± 0.41, Δ*arcrz1* = 2.03 mm ± 0.33 and Δ*arcrz1/ArCRZ1* = 3.98 mm ± 0.56). The mean and standard deviation (±) were calculated from three biological replicates, counting at least 20 plants for each replicate. These results were quantified using one-way ANOVA, compared with the control (****$p < 0.0001$). Each red dot represents the average value of all technical replicates in biological replicate used for the quantitative analysis.

Fig 10. ArF-BAR regulated functions in *A. rabiei* cells and its regulation by ArCRZ1.

On encounter with the pathogen, plant generates various stresses around the site of pathogen recognition; a common stress is oxidative stress. The perception of these stresses by the pathogen, results in the upregulation of cytosolic Ca^{2+} within the pathogen. Here, in *A. rabiei*, increased calcium level, sensed by calmodulin mediates the activation of calcineurin. Activated calcineurin dephosphorylates cytoplasmic inactive ArCRZ1 (phosphorylated form of ArCRZ1 remains inactive). Dephosphorylated of ArCRZ1 results in its translocation to the nucleus where it regulates the expression of *ArF-BAR* and other genes to cope with stress. Further, during polarized growth this ArF-BAR gets localized to hyphal tip where it leads to generation and stabilization of membrane curvature which is crucial for endocytosis and helps in the secretion of effector proteins required for fungal virulence. Additionally, ArF-BAR protein mediates actin organization and helps in septa formation.

MUPPLEMENTARY FIGURE LEGENDS

S1 Fig. Maximum likelihood phylogeny of F-BAR sequences.

Sequences used: *Ascochyta rabiei*, *Parastagonospora nodorum*, *Alternaria alternate*, *Bipolaris zeicola*, *Bipolaris victoriae*, *Bipolaris oryzae*, *Bipolaris sorokiniana*, *Bipolaris maydis*, *Sclerotinia sclerotiorum*, *Botrytis cinerea*, *Marssonina brunnea*, *Histoplasma capsulatum*, *Blastomyces dermatitidis*, *Blastomyces gilchristii*, *Emmonsia crescens*, *Paracoccidioides brasiliensis*, *Uncinocarpus reesii*, *Coccidioides immitis*, *Aspergillus nidulans*, *Aspergillus rambellii*, *Aspergillus ochraceoroseus*, *Aspergillus kawachii*, *Aspergillus lacticoffeatus*, *Aspergillus oryzae*, *Aspergillus flavus*, *Aspergillus parasiticus*, *Aspergillus nomius*, *Aspergillus terreus*, *Aspergillus fumigatus*, *Aspergillus lentulus*, *Aspergillu sclavatus*, *Aspergillus fischeri*, *Trichoderma reesei*, *Cordyceps confragosa*, *Fusarium oxysporum*, *Fusarium verticillioides*,

Fusarium fujikuroi, *Fusarium graminearum*, *Fusarium pseudograminearum*, *Fusarium langsethiae*, *Neonectria ditissima*, *Metarhizium anisopliae*, *Metarhizium acridum*, *Pyricularia grisea*, *Pyricularia oryzae*, *Verticillium dahlia*, *Neurospora crassa*, *Drosophila melanogaster*, *Schizosaccharomyces pombe*, *Phytophthora sojae*, *Phytophthora graminis*, *Phytophthora striiformis*, *Ustilago maydis*, *Rhizopus delemar*, *Cryptococcus*, *Phytophthora infestans*, *Yarrowialipolytica*, *Kluyveromyces lactis*, *Naumovozymacastellii*, *Saccharomyces cerevisiae*, *Candida glabrata*, *Eremotheciumgossypii*, *Clavisporalusitaniae*, *Meyerozymaguilliermondii*, *Candida parapsilosis*, *Candida tropicalis*, *Candida dubliniensis*, *Candida albicans*, Ganodermalucidum, and *Homo sapiens*. The multiple sequence alignment of protein was performed by PROMALS3D software and the phylogeny was constructed using a software MEGA7.0.21. The bootstrap values, derived from 1000 iterations, validated the obtained phylogeny.

S2 Fig. Conserved nature of ArF-BAR protein.

Multiple sequence alignment showing the conservation of ArF-BAR protein with BZZ1p of *S. cerevisiae*, Cdc42-interacting protein 4 (CIP4) of *Drosophila* and Syndapin proteins of *Drosophila*. Colour code for sequence conservation varies from blue (least conserved) to red (highly conserved). The alignment of the protein is determined by Praline software using default parameters. The black box marks the presence of positively charged residues of F-BAR domain. Asterisk (*) represents the residues in C1 domain of Protien Kinase C1 (PKC1), required for interaction with DAG.

S3 Fig. Expression and purification of ArF-BAR protein in *E. coli.*

(A) His purification of bacterially expressed ArF-BAR protein. Analysis of the purification of recombinant ArF-BAR as shown by SDS-PAGE. (B) Intense tubular network in synthetic liposomes is formed after 30 min incubation with purified recombinant ArF-BAR protein. Inset showing the enlarged view of dense tubular network originating from a liposome. (C- D) His purification of ArF-BAR^{mut1}, ArF-BAR^{mut2}. The protein was visualised by Comomassie Brilliant Blue staining. (UI, crude extract of un-induced samples after centrifugation; I, crude extract of induced samples after centrifugation; FT, flow-through fraction of the Nickel chelating resin column; W5- 5th wash fraction of the Nickel chelating resin column; E1, E2 and E3 eluate fractions of the Nickel chelating resin column showing the purified ArF-BAR protein). Protein standards are shown (M), and their masses are indicated in kDa.

S4 Fig. The schematic representation of constructs used to generate Δ*arf-bar* mutant complemented strains.

(A, B, C) Constructs used to generate different Δ*arf-bar* mutant complemented strains (Δ*arf-bar/ArF-BAR,* Δ*arf-bar/ArF-BAR*^{mut1} and Δ*arf-bar/ArF-BAR*^{mut2}) under the control of native promoter of *ArF-BAR* gene.

S5 Fig. Southern hybridization confirmation of the successful replacement of *ArF-BAR* gene with hygromycin cassette and complementation.

(A) The schematic representation of *A. rabiei* knockout mutant generation by the homologous recombination approach to obtain targeted *ArF-BAR* gene deletion mutants (Δ*arf-bar*). The bar

represents the genomic region used to generate probe for Southern confirmation. (B) The representative Southern blot confirming successful *ArF-BAR* gene deletion (Δ*arf-bar*), with a single integration of *hph* at replacement site. Along with the confirmation of *ArF-BAR* complementation in Δ*arf-bar*, followed successful generation of Δ*arf-bar/ArF-BAR*^{mut1}and Δ*arf-bar/ArF-BAR*^{mut2} complementation.

S6 Fig. Disease symptoms on AB susceptible chickpea plants after 10 days post inoculation. The susceptible plants inoculated with conidia of *A. rabiei* (WT), Δ*arf-bar/ArF-BAR* and Δ*arf-bar/ArF-BAR*^{mut2}, showed severe disease symptoms with the increasing duration after inoculation. Δ*arf-bar and* Δ*arf-bar/ArF-BAR*^{mut1} challenged plants showed no or very mild symptoms after 10 dpi.

S7 Fig. Evaluation of chickpea host cells penetration by *A. rabiei* (WT) and Δ*arf-bar* strains growing hyphae.

(A, B) Confocal images showing the depth of penetration 48 hpi by *A. rabiei* (WT) and Δ*arf-bar* strains, respectively, in AB susceptible chickpea leaves. Fungal hyphae were stained with WGA-488 for visualization, prior to microscopy. The *Z*-stacked images were acquired till 23 µm depth, starting from the surface of the leaves. The image is the representation of maximum projections of all the Z-stacks. Scale bar = 5 µm. (C) The bar graph, representing mean and SD, shows the difference in ability to penetrate within the host by *A. rabiei* (WT) and Δ*arf-bar*. The results were quantified using Student's t-test one tailed compared to its control (*$p < 0.0079$).

S8 Fig. Growth inhibition in colony morphology of Δ*arf-bar* mutant and ArF-BAR complementation strains in the presence of various oxidative stresses.

The *A. rabiei* (WT), Δ*arf-bar* mutant and mutant complemented strains, observed 10 days after incubation at 22ºC. For oxidative stress, the PDA was supplemented with 250 µM and 500 µM menadione and 2mM H_2O_2. Strains of Δ*arf-bar* and Δ*arf-bar/ArF-BAR*^{mut1} exhibited more growth inhibition towards oxidative stress condition as compared to WT.

S9 Fig. Distribution of ArF-BAR::EYFP during *in planta* infection.

Confocal micrographs showing the punctate distribution of ArF-BAR::EYFP during host infection. The representative image is the maximum intensity projection of all the Z-stack images with 0.5 µm step size, acquired after 48 hpi of susceptible chickpea with fungal conidia expressing chimeric ArF-BAR::EYFP. Scale bar = 5 µm (n= 12).

S10 Fig. Germination of fungal conidia of *A. rabiei* and Δ*arf-bar* on hydrophobic surface.

Difference in growth pattern on hydrophobic surfaces was slightly observed and microscopic photographs (20x) were taken after 12 h of conidial spread.

S11 Fig. Homodimerization of F-BAR indicating conserved nature.

Split-Ubiquitin based Y2H system was used to determine the homodimerization between ArF-BAR proteins. Plates were photographed after 48 h of yeast growth. Strong positive interaction between two ArF-BAR proteins was reflected with the growth on QDO (SD/-L/-W/-A/-H) media and X-gal overlay assay to check the activation *LacZ* gene.

S12 Fig. F-BAR domain of ArF-BAR is not the direct member to interact.

(A) The yeast two-hybrid result showing the positive interaction of ArF-BAR protein with ArWASp. (B) F-BAR domain (1-325 amino acids) of ArF-BAR protein [F-BAR($_{\text{ArF-BAR}}$)] failed to interact with ArWASp in Y2H system. Plates were photographed 48 h after yeast spotting. The interaction was confirmed bythree independent replicates.

S13 Fig. Characterization of ArCRZ1 and confirmation of gene knockout and complementation.

(A) Schematic representation of domain organisation of ArCRZ1 protein. (B) The schematic map showing the homologous recombination based knockout approach used for targeted *ArCRZ1* gene deletion mutant (Δ*crz1*) strain generation. (C) Schematic representation of Δ*arcrz1/ArCRZ1* complementation construct under the native promoter of *ArCRZ1*. The genomic region used to generate probe for confirmation by Southern hybridization is being highlighted. (D) The Southern blot result confirmed successful *ArCRZ1* gene deletion (Δ*arcrz1*), with single integration of *hph* at replacement site along with the complementation confirmation of *ArCRZ1* in Δ*arcrz1* mutant.

S14 Fig. Growth phenotypes of *A. rabiei* (WT), Δ*arcrz1* and Δ*arcrz1/ArCRZ1* under various stress conditions.

(A) Colony morphology and growth assay of *A. rabiei* (WT), Δ*arcrz1* mutant and Δ*arcrz1/ArCRZ1* complementation strains observed under various stress conditions after 10 days incubation at 22ºC. PDA is supplemented with 250 µM menadione and 2mM H_2O_2, 0.07 M, 0.2 M, 0.4 M $CaCl_2$ and 0.01% SDS. The Δ*arcrz1* mutant exhibited extreme sensitivity towards

various stress conditions as compared to *A. rabiei* (WT). The mutant strain was highly sensitive towards $CaCl_2$ and fails to grow at higher concentration of 0.4 M. (B) The bar graph represents mean and SD of radial diameter for the fungal strains in the presence of various stresses. All the growth assays were performed in triplicates. The results were quantified using two-way ANOVA, Tukey's multiple comparisons. The statically significant differences are shown with ****$p < 0.0001$, ***$p < 0.001$, **$p < 0.01$, *$p < 0.05$, ns = non-significant. Red dots represent the value of each biological replicate/ sample used for the quantitative analysis.

SUPPLEMENTARY TABLE LEGENDS

S1 Table: YEASTRACT result for the putative transcription factors bindings on the upstream regulatory sequences of ArF-BAR.

S2 Table: Ascochyta rabiei strains used in this study.

S3 Table: List of oligonucleatides used in this study.

Figure 1

Click here to access/download;Figure;Fig1.tif

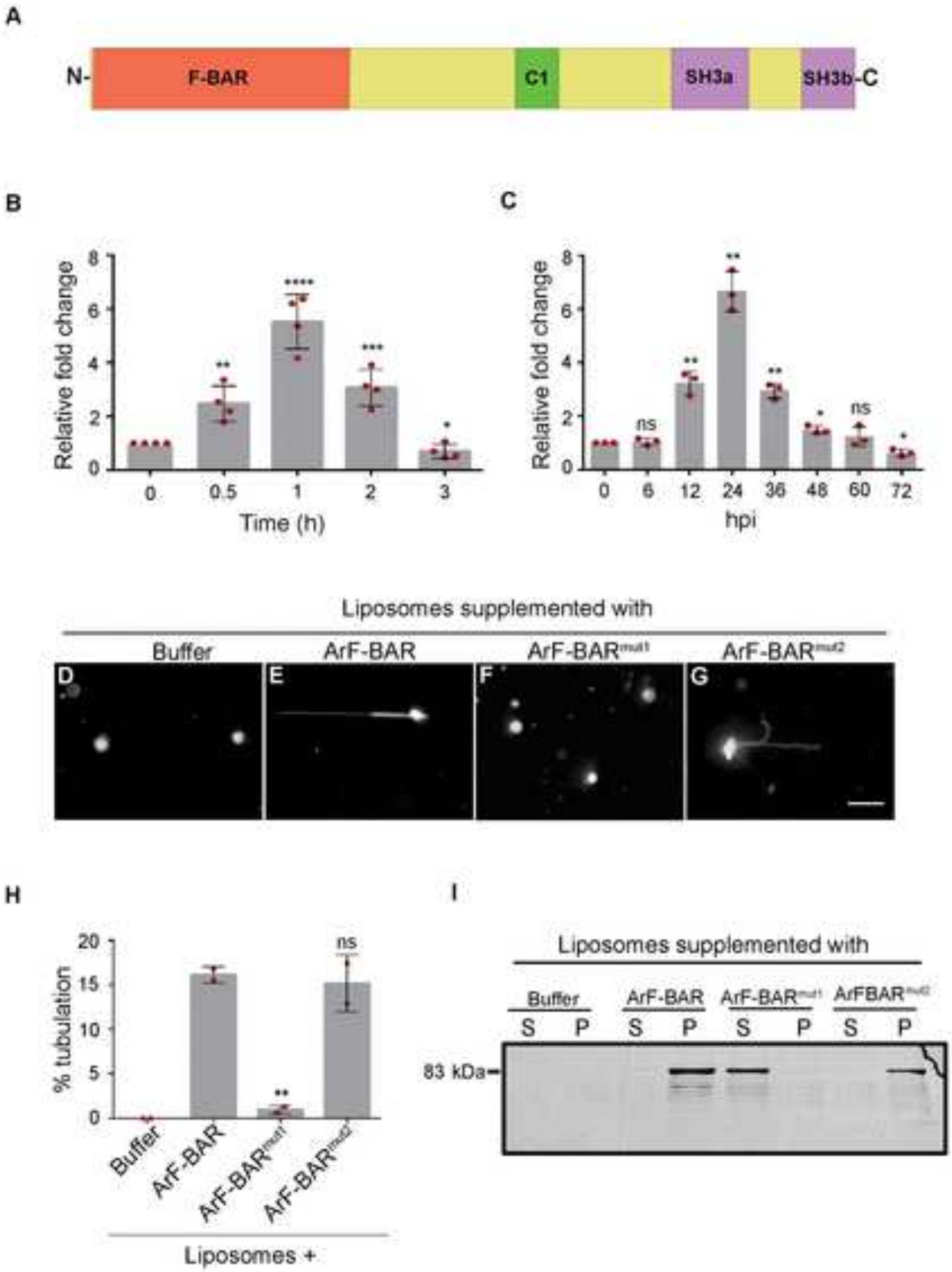

Figure 2

Click here to access/download;Figure;Fig2.tif

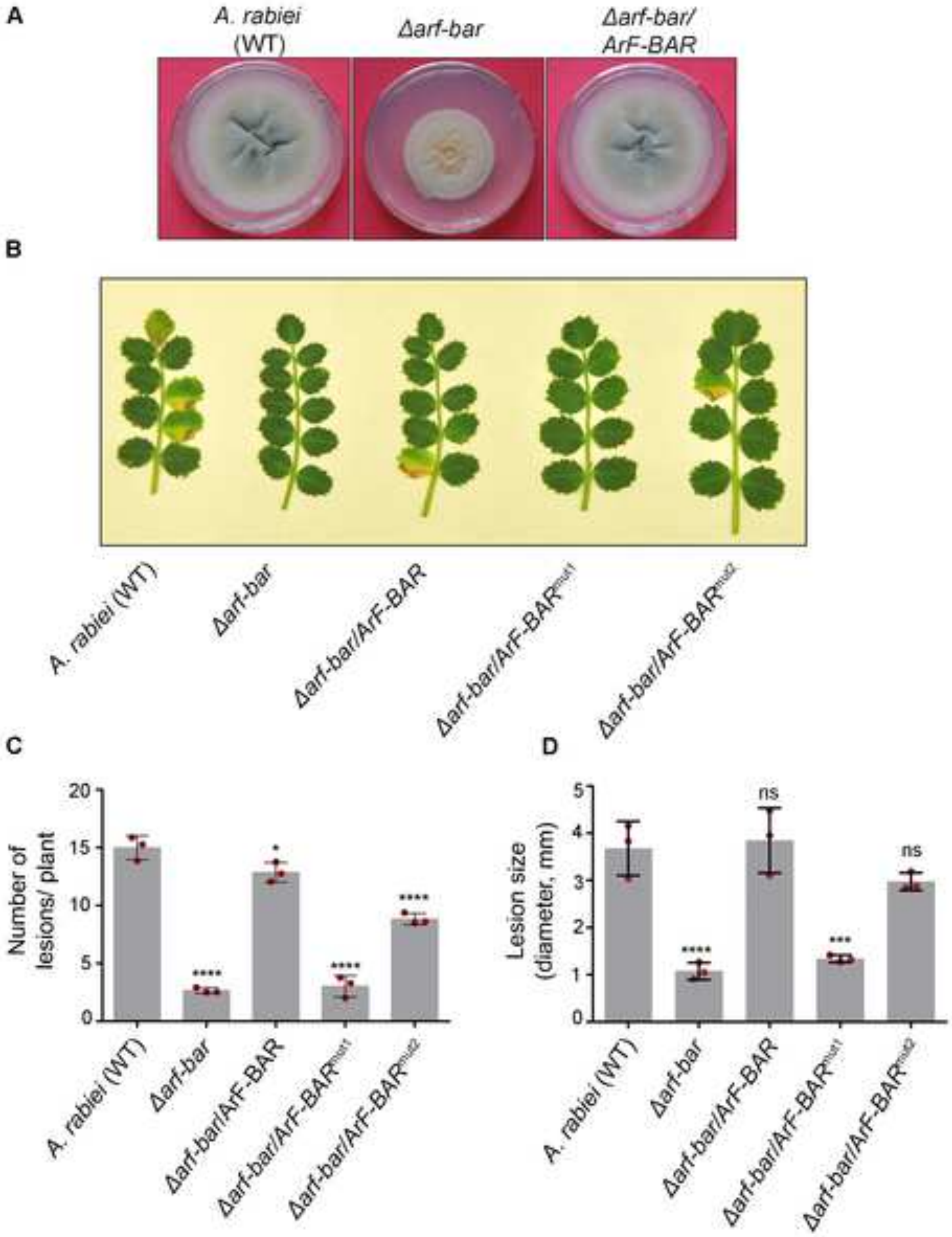

Figure 3

Click here to access/download;Figure;Fig3..tif

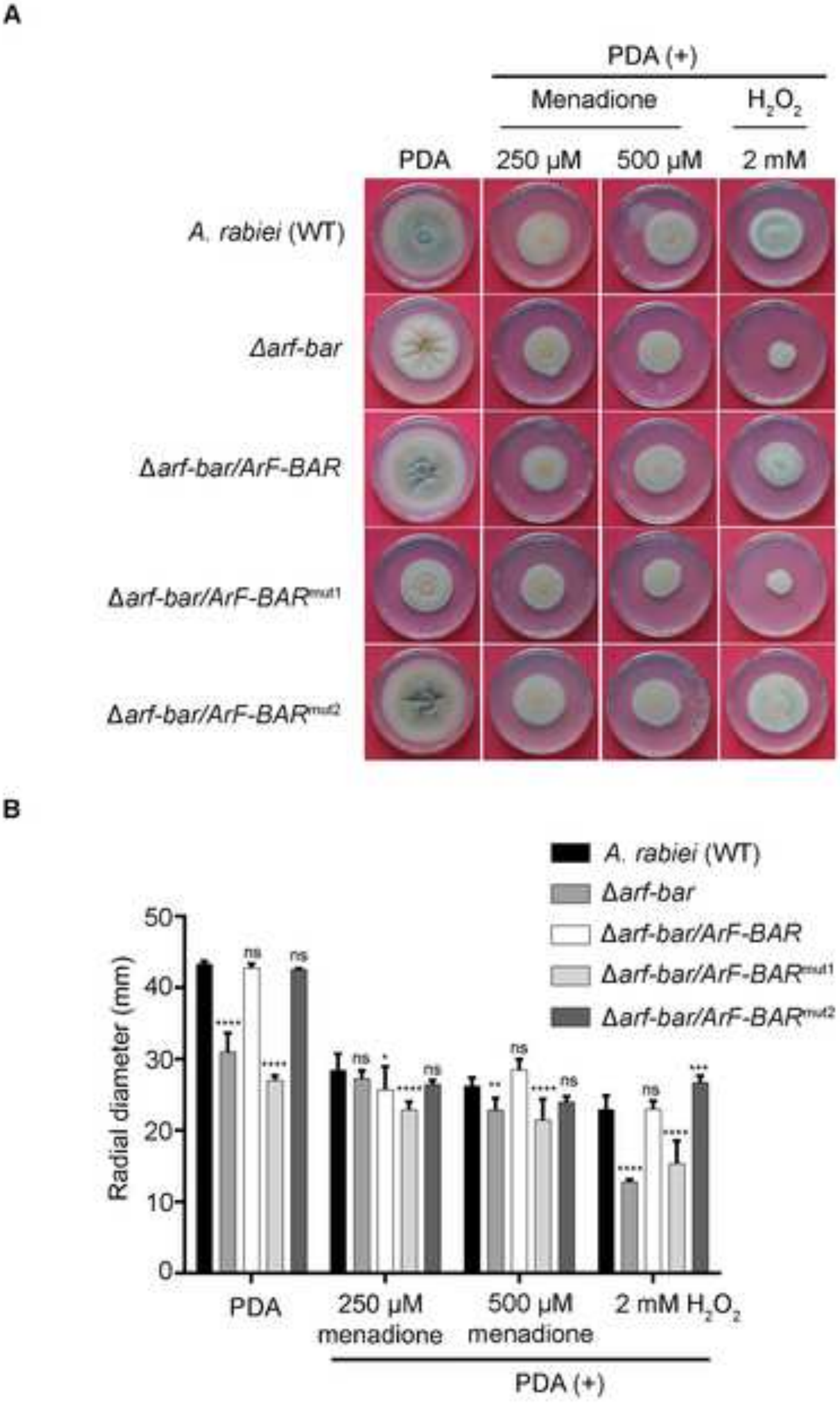

Figure 4

Click here to access/download;Figure;Fig4 new.tif

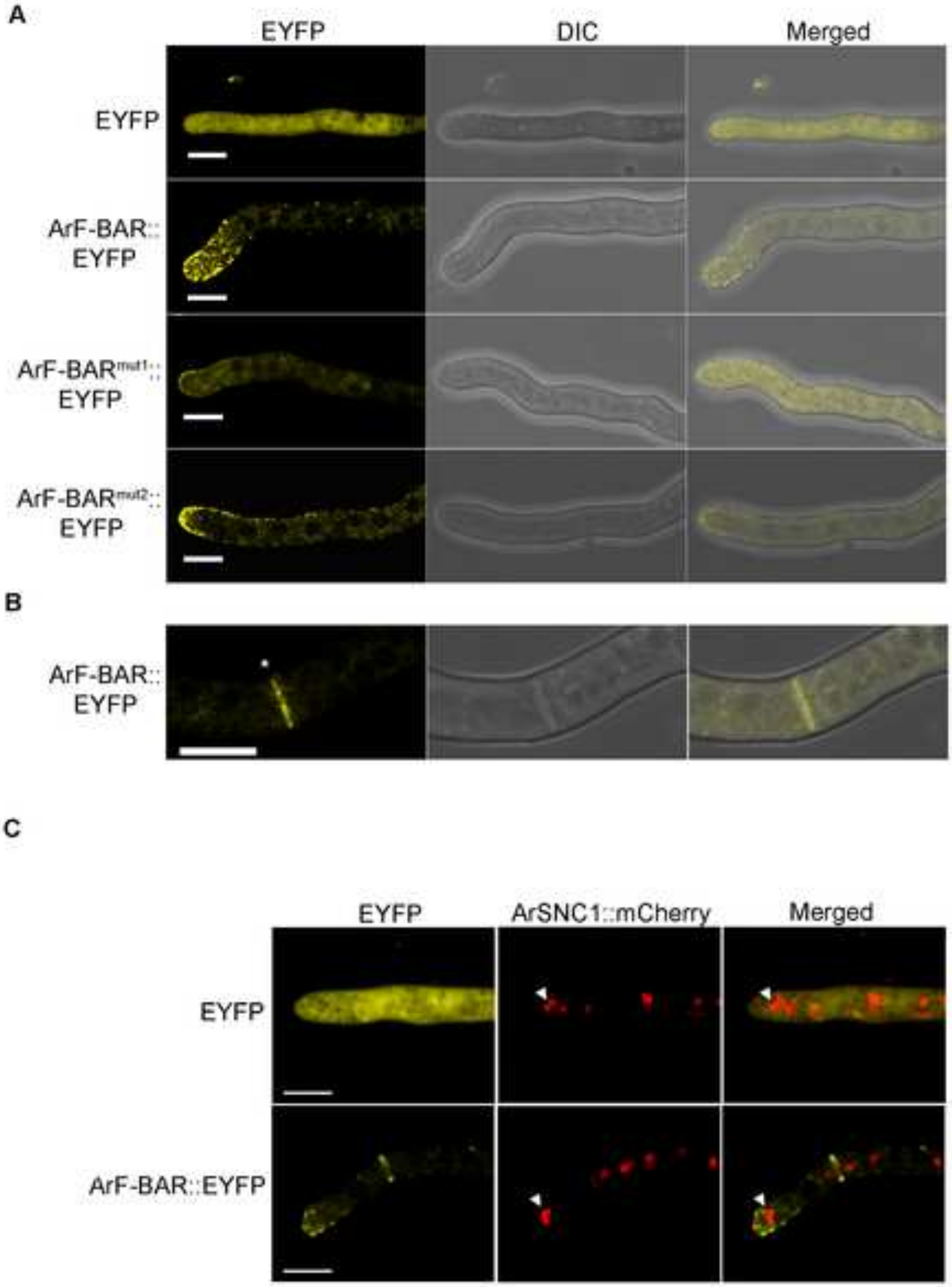

Figure 5

Click here to access/download;Figure;fig 5 new.tif

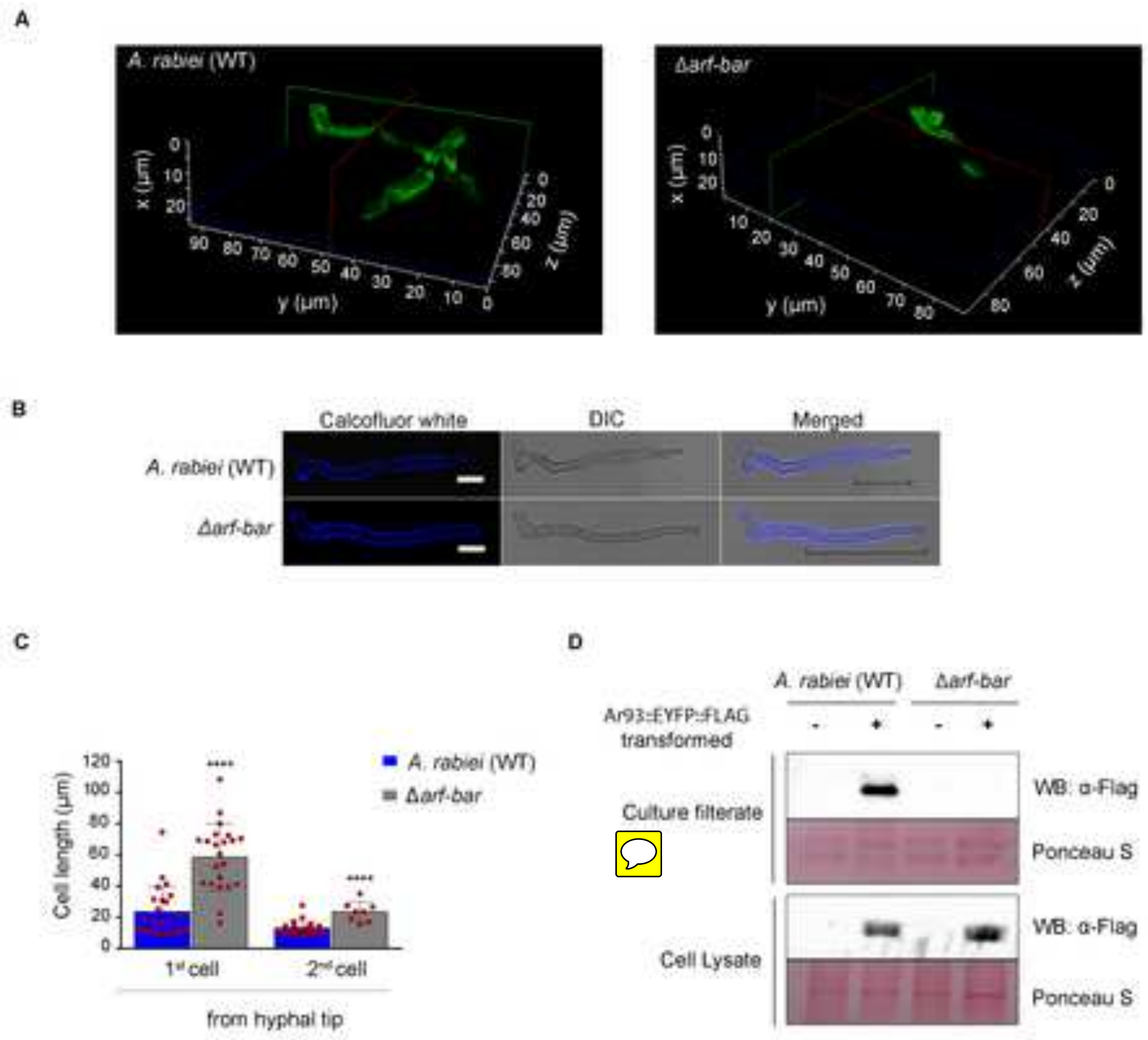

Figure 6

Click here to access/download;Figure;Fig6.tif

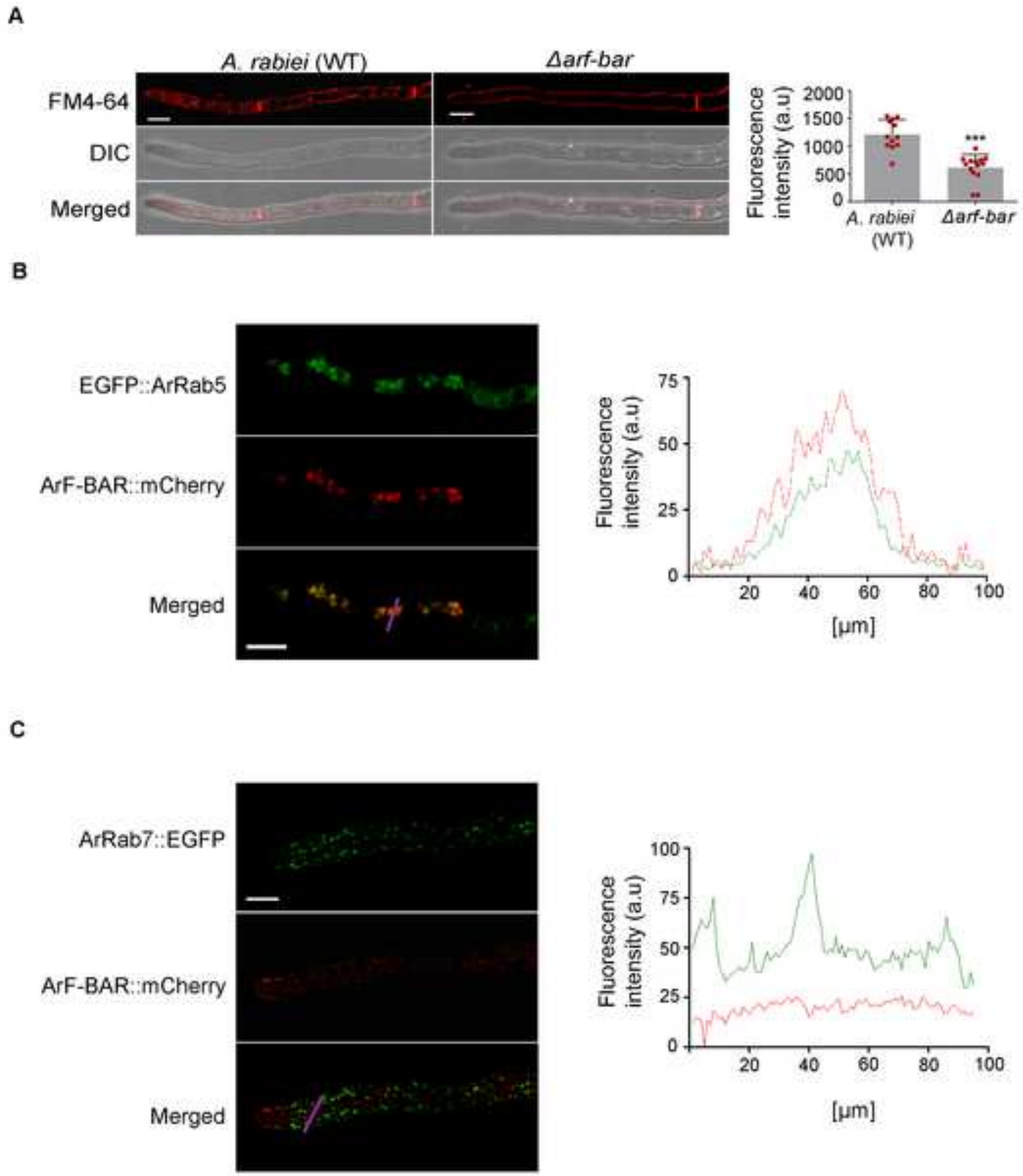

Figure 7

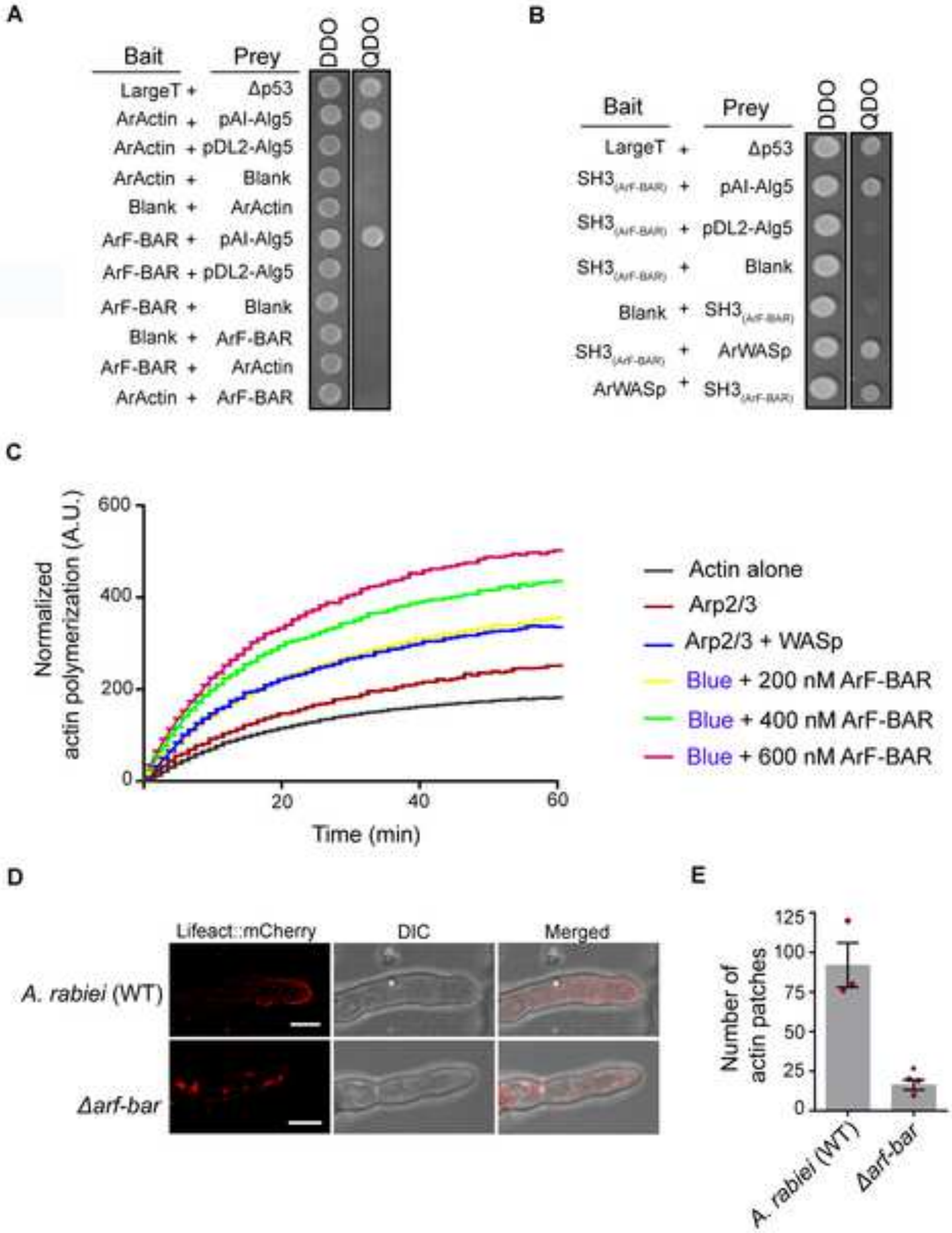
A
Bait
Prey
DDO
QDO
LargeT + Δp53
ArActin + pAl-Alg5
ArActin + pDL2-Alg5
ArActin + Blank
Blank + ArActin
ArF-BAR + pAl-Alg5
ArF-BAR + pDL2-Alg5
ArF-BAR + Blank
Blank + ArF-BAR
ArF-BAR + ArActin
ArActin + ArF-BAR
B
Bait
Prey
DDO
QDO
LargeT + Δp53
SH3(ArF-BAR) + pAl-Alg5
SH3(ArF-BAR) + pDL2-Alg5
SH3(ArF-BAR) + Blank
Blank + SH3(ArF-BAR)
SH3(ArF-BAR) + ArWASp
ArWASp + SH3(ArF-BAR)
C
Normalized actin polymerization (A.U.)
600
400
200
0
20
40
60
Time (min)
Actin alone
Arp2/3
Arp2/3 + WASp
Blue + 200 nM ArF-BAR
Blue + 400 nM ArF-BAR
Blue + 600 nM ArF-BAR
D
Lifeact::mCherry
DIC
Merged
A. rabiei (WT)
Δarf-bar
E
Number of actin patches
125
100
75
50
25
0
A. rabiei (WT)
Δarf-bar

Figure 8

Click here to access/download;Figure;Fig8-new.tiff

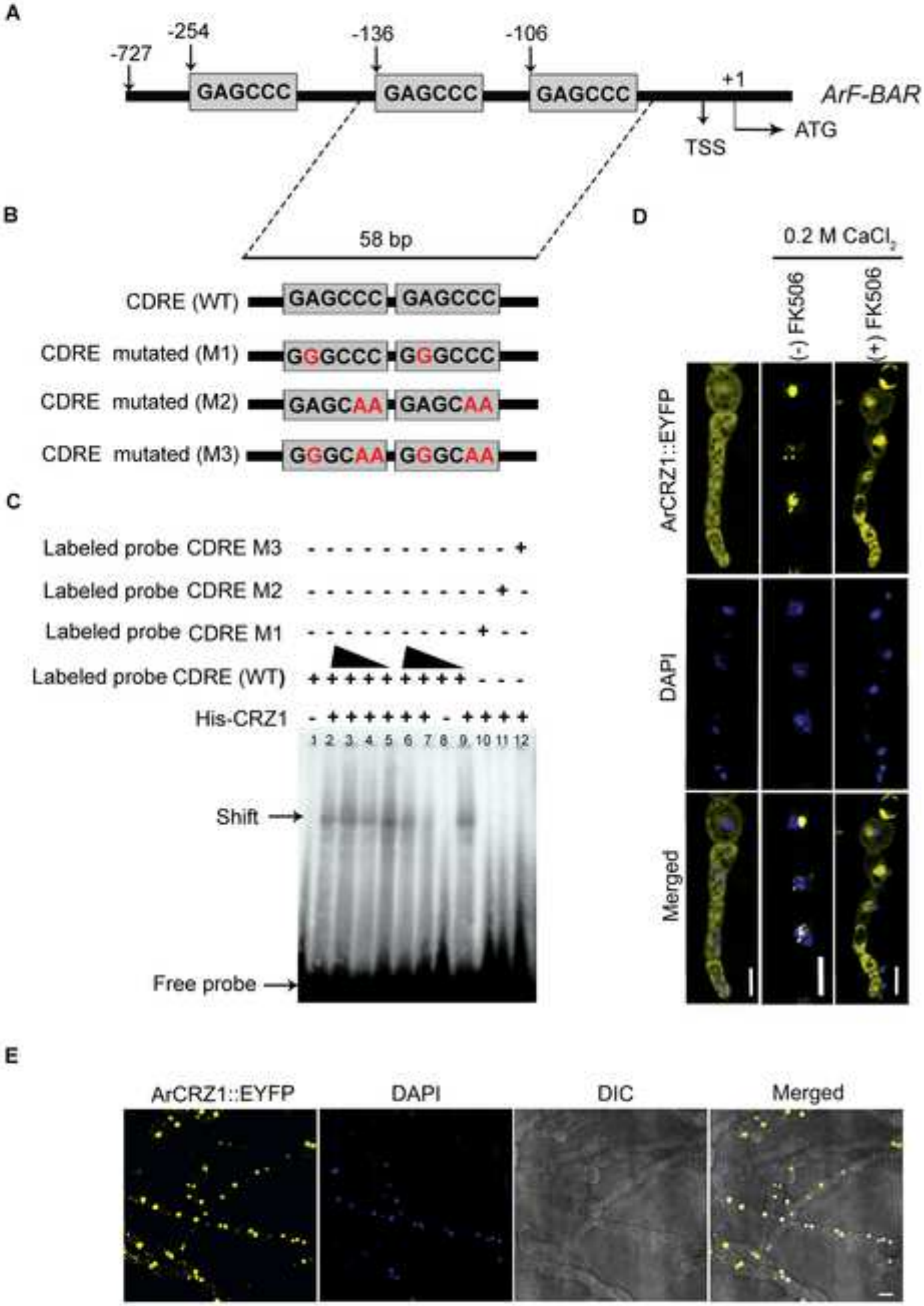

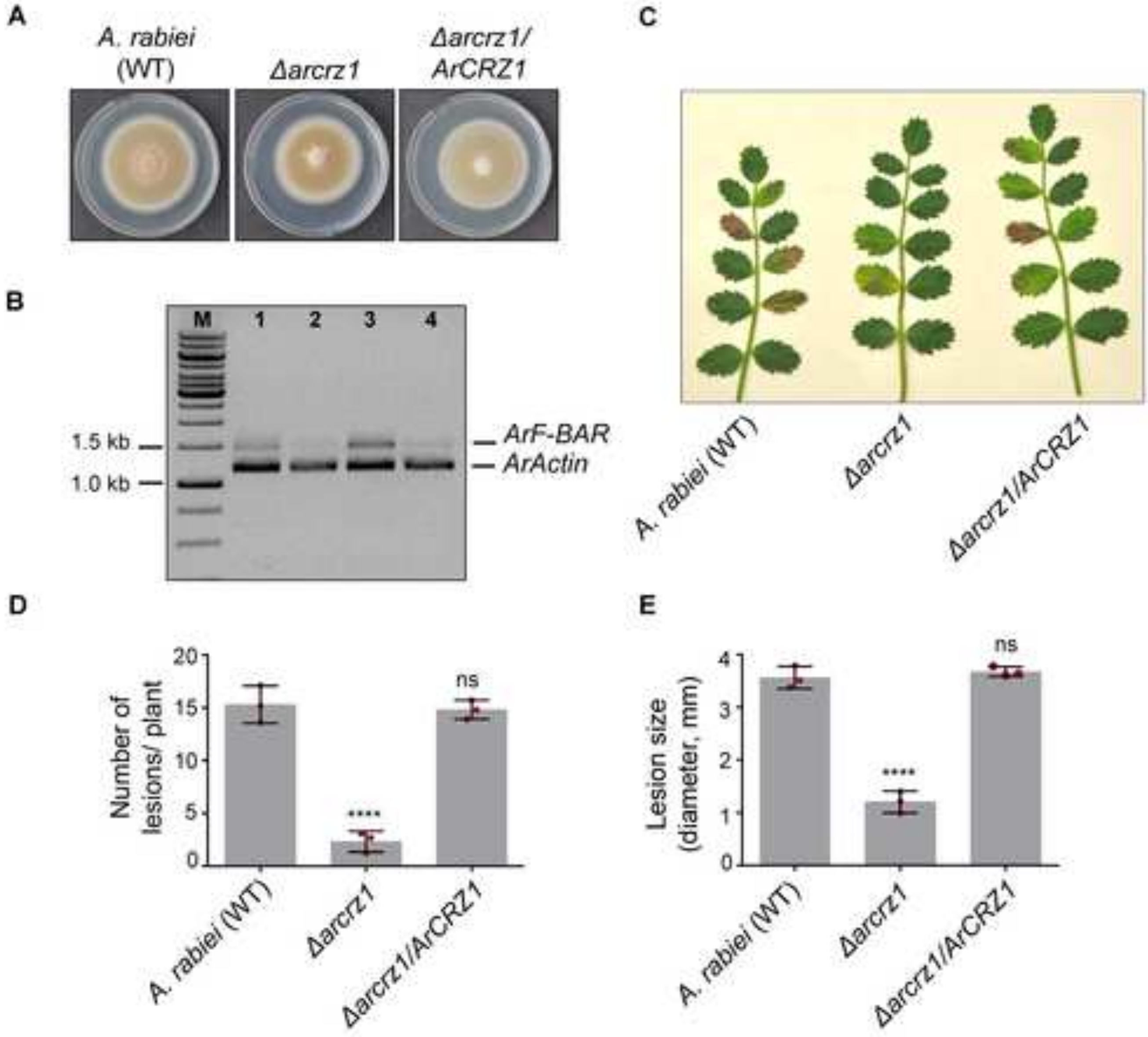
A
A. rabiei (WT)
Δarcrz1
Δarcrz1/ ArCRZ1
B
M 1 2 3 4
1.5 kb
1.0 kb
ArF-BAR
ArActin
C
A. rabiei (WT)
Δarcrz1
Δarcrz1/ArCRZ1
D
Number of lesions/ plant
0
5
10
15
20
****
ns
A. rabiei (WT)
Δarcrz1
Δarcrz1/ArCRZ1
E
Lesion size (diameter, mm)
0
1
2
3
4
****
ns
A. rabiei (WT)
Δarcrz1
Δarcrz1/ArCRZ1

Click here to access/download;Figure;Fig10.tif

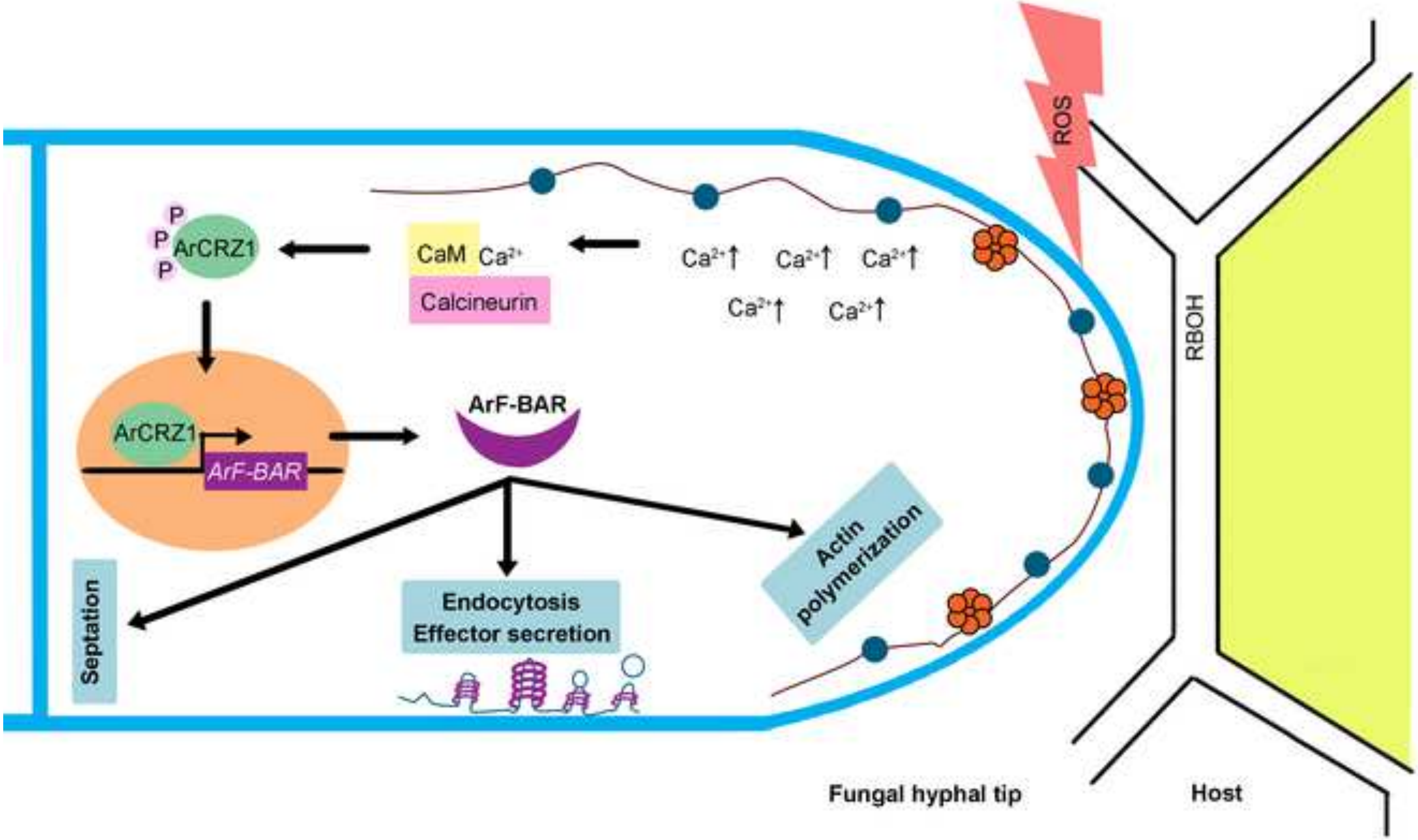

Click here to access/download
Supporting Information
All Supplementary figures.pdf

Supplementary tables

Click here to access/download
Supporting Information
Supple Tables_plos genetics.pdf

Modulation of fungal virulence through CRZ1 regulated F-BAR-dependent actin remodeling and endocytosis in chickpea infecting phytopathogen *Ascochyta rabiei*

Manisha Sinha[1¶], Ankita Shree[1¶], Kunal Singh[1#], Kamal Kumar[1], Shreenivas Kumar Singh[1], Vimlesh Kumar[2], Praveen Kumar Verma[1,3*]

[1]Plant Immunity Laboratory, National Institute of Plant Genome Research, Aruna Asaf Ali Marg, New Delhi-110067, India.

[2]Department of Biological Sciences, Indian Institute of Science Education and Research Bhopal (IISER-Bhopal), Bhauri, Bhopal-462066, Madhya Pradesh, India

[3]Plant Immunity Laboratory, School of Life Sciences, Jawaharlal Nehru University, New Delhi-110067, India

[¶]These authors contributed equally to the work

[*]Corresponding author

[#]Present address: Biotechnology Division, CSIR-Institute of Himalayan Bioresource Technology, Palampur-176 061, Himachal Pradesh, India.

Abstract

Polarized hyphal growth of filamentous pathogenic fungi is an essential event for host penetration and colonization. The long-range early endosomal trafficking during the hyphal growth is crucial for nutrient uptake, sensing of host-specific cues, and regulation of effector production. Bin1/Amphiphysin/Rvs167 (BAR) domain-containing proteins mediate fundamental cellular processes, including membrane remodeling and endocytosis. Here, we identified a F-BAR domain protein (ArF-BAR) in the necrotrophic fungus *Ascochyta rabiei* and demonstrate

its involvement in endosome-dependent fungal virulence on the host plant *Cicer arietinum*. We show that ArF-BAR regulates endocytosis at the hyphal tip, localizes to the early endosomes, and is involved in actin dynamics. Functional studies involving gene knockout and complementation experiments reveal that ArF-BAR is essential for virulence. The loss-of-function of ArF-BAR results in delayed formation of ~~first septa~~ apical septum from the growing hyphal tip, crucial for host penetration and ~~proliferation~~impaired secretion of a candidate effector having signal peptide. The mRNA transcripts of ArF-BAR were ~~was~~ induced in response to oxidative stress and infection~~and localized to endocytic vesicles wihin the fungal hyphae~~. We also show that ArF-BAR is able to tubulate synthetic liposomes, suggesting the functional role of F-BAR domain in membrane tubule formation *in vivo*. Further, our studies identified a stress-induced transcription factor, ArCRZ1 (Calcineurin-responsive zinc finger 1) as key transcriptional regulator ~~for transcriptional reprogramming~~of ArF-BAR. We propose a model in which ArCRZ1 functions upstream of ArF-BAR to regulate *A. rabiei* virulence through a mechanism that involves endocytosis, ~~exocytosis~~effector secretion, and actin cytoskeleton regulation.

Author summary

BAR-domain superfamily is known to mold amorphous lipid bilayer into defined tubular shapes and ~~critical~~ required for endosome formation and trafficking. Although these proteins were studied earlier in context of their structural and biochemical properties, there is limited evidence on the direct role of F-BAR domain proteins in the pathophysiological development of other economically important fungi. Our study assumes functional significance for plant infection as we identified an F-BAR domain-containing protein that is regulated by a distinct transcriptional

network regulated by CRZ1. We characterized F-BAR in a necrotrophic fungal pathogen, *Ascochyta rabiei*, which that causes the *Ascochyta* blight disease in chickpea plants. Additionally, we have also identified a calcium-regulated CRZ1 transcription factor that regulates the transcription of *ArF-BAR*. Our study will help to understand the complex interplay a signaling cascade that regulates underlying the endosome formation required for fungal virulence.

Formatted: Font: Italic

Introduction

Polarized hyphal growth is a signature feature of filamentous fungi during host colonization [1] . This feature allows the fungus to sense, coordinate, and respond to an array of cues from the host [2] . Therefore, regulation of hyphal tip growth is one of the major virulence determinants in filamentous fungi. In response to infection, the plant innate immune system recognizes pathogen and fosters effective defense response. Pathogens must recognize the plant surface cues and counter host-generated defense responses for effective pathogenesis. Additionally, invasive fungi must overcome an intracellular challenge posed by the distance between the elongated invading hyphal architecture and the nucleus [3]. Mounting evidence strongly suggests that the complications associated with this increased distance are overcome by long-distance intracellular communication for rapid and precise transduction of external information [4]. Moreover, the maintenance of extremely polarized hyphal morphology is heavily dependent on endosome trafficking. It is also important to maintain the structural and functional features of a fungal cell [5].

In the case of filamentous fungi, long-distance signaling is mediated by endosomes. Besides signal sensing for motor-dependent retrograde signaling, they are involved in the

recycling of cell wall components, polarisomes, and various receptors required for polarized tip growth [6]. Loss of these functions leads to impaired host invasion and virulence [7, 8]. The generation of early endosomes (EEs), which are multipurpose carriers, is a key step in the endocytic pathway and involves the cooperative action of membrane bending and cytoskeleton reorganization. Membrane bending is the cornerstone for the generation of EEs and is regulated by proteins involved in the detection and stabilization of membrane curvature [9,10] .

In animal cells, BAR domain superfamily proteins have been shown to integrate membrane dynamics with cytoskeletal changes [11]. The F-BAR domain proteins form possessN-terminal α-helical coiled-coil dimers and bind to negatively charged lipid membranes via their positively charged residues. This binding generates membrane curvature and regulates intracellular vesicle trafficking [12]. Depending on the degree of curvature of thein dimer of BAR proteins, the BAR membrane domain superfamily proteins are broadly classified into three families: classical BAR and N-BAR, Fer/CIP4 homology BAR (F-BAR) and inverse BAR (I-BAR) proteins. The classical BAR domain (N-BAR) and F-BAR domain proteins induce positive membrane curvature through concave lipid-binding interfaces and trigger cell membrane invagination. However, I-BAR domains interact with shallow negatively curved membranes through convex lipid-binding interfaces, leading to cell membrane protrusion [13, 14]. Thet pioneering Pioneering work in the corn smut fungus, *Ustilago maydis*, revealed the importance of endocytosis for the pathogenic development and virulence of the filamentous fungi by showing impaired early pathogenicity and germination in endocytic mutants [15]. The F-BAR protein, Cdc15, is involved in cytokinetic ring and septa formation in *U. maydis* [16] . Recent studies in *Magnaporthe oryzae* revealed the importance of N-BAR domain-containing proteins in the growth and virulence of filamentous fungi [17]. Further, the I-BAR protein, Rvs167, was

found to be involved in the extension of the rigid penetration peg required during *M. oryzae* invasion [18]. The ~~F-BAR protein like~~F-BAR-like protein Bzz1p in yeast and Cip4 in *Drosophila* acts during the early stages of endocytosis in the formation of actin patches [19,20]. It triggers actin polymerization via the Arp2/3 complex [21]. There has been an intense study on the structural and biochemical properties of BAR domain proteins that contribute to their mode of action [11,13]. However, there has been limited functional characterization of BAR superfamily proteins in phytopathogenic fungi.

Ascochyta rabiei (Pass.) Labr. [teleomorph *Didymella rabiei*, ArD2; ITCC No. 4638], a causal agent of *Ascochyta* blight (AB) disease in chickpea (*Cicer arietinum* L.), is one of the most devastating necrotrophic phytopathogens. *A. rabiei* infects the above-ground parts of this legume plant and greatly reduces the yield of the crop [22]. The fungal hyphae aggregate in the cortical cells of the chickpea plant and differentiate into asexual spores called pycnidia [23,24]. The genome of *A. rabiei* has been sequenced and analyzed to identify pathogenic determinants [25]. *A. rabiei* has emerged as an interesting model system for elucidating the cell biology, especially the endocytic machinery, during polar growth and pathogenesis in necrotrophic phytopathogenic fungi.

In this study, an F-BAR domain-containing protein, ArF-BAR, was identified and characterized in *A. rabiei*. A loss of function mutation of ArF-BAR caused ~~a~~, delayed septa formation, severely compromised fungal ~~and delayed septa formation~~ virulence on chickpea and secretion of Ar93, which is a candidate effector of *A. rabiei* having a classical signal peptide sequence. The results showed that the F-BAR domain of the ArF-BAR protein binds to and deforms synthetic liposomes and generates membrane tubules. *ArF-BAR* transcripts were induced in response to oxidative stress and infection, and localized to endocytic vesicles within

Formatted: Font: Italic

Formatted: Font: Italic

the fungal hyphae. It was also found that *ArF-BAR* expression was regulated by a stress inducible transcription factor, ArCRZ1. Our The data suggested that ArF-BAR-dependent actin cytoskeleton dynamics combined with endocytosis and exocytosis effector secretion is was crucial for *A. rabiei* virulence.

Results

ArF-BAR expression is induced in response to oxidative stress and infection

Transcriptome analysis of *A. rabiei* during oxidative stress has provided a greater understanding of the survival strategies used by necrotrophic fungi against host-generated oxidative stress [26]. The study by Singh et al., [26] revealed that 70 unigenes were upregulated under oxidative stress conditions. Of these 70 unigenes, a clone resembling "ST47_g8005" of the sequenced *A. rabiei* genome [25], showed early upregulation against oxidative stress. The deduced amino acid sequence of this gene revealed a protein with four distinct domains: An N-terminal F-BAR domain (7–253 amino acids), a unique C1 domain (407–459 amino acids) showing similarity to the C1 domain of Protein Kinase C1 (PKC1), and two consecutive C-terminal SH3 domains (572–651 and 705–760 amino acids) (Fig 1A). Henceforth, this protein has been named as ArF-BAR. Phylogenetic analysis of selected pathogenic fungi and other eukaryotes revealed that ArF-BAR shared sequence identity with proteins of many closely related fungal pathogens (S1 Fig). ArF-BAR was found to share approximately 33% and 41% sequence identity with *Saccharomyces cerevisae* BZZ1p and *Drosophila melanogaster* Cdc42-Interacting Protein 4 (CIP4) proteins, respectively (S2 Fig).

To validate the results of the transcript profiling, quantitative real-time PCR (qRT-PCR) was performed using primers specific to the F-BAR domain encoding region. The qRT-PCR experiments revealed a higher expression level of the *ArF-BAR* transcript in *A. rabiei* mycelia

after a 1 h treatment of menadione, which is an oxidative stress generator (Fig 1B). To directly assess *ArF-BAR* transcript induction during pathogen in proliferation on a susceptible chickpea variety (PUSA-362), the time course of *ArF-BAR* transcript expression was measured following plant inoculation using qRT-PCR after inoculating plants with *A. rabiei* conidial suspensionusing qRT-PCR. Consistent with the previous observations [26], a significant increase in the *ArF-BAR* transcript level was found following infection. The maximum transcript level was at 24 hours post-inoculation (hpi; Fig 1C), which is within the critical time period for spores to germinate on the host surface and early germinated conidial hyphae starts penetrating chickpea tissue [24,27].

ArF-BAR is a membrane tubulating protein

To understand whether ArF-BAR has a functional F-BAR domain, synthetic liposomes containing Rhodamine B-conjugated PE were incubated with purified His-tagged recombinant ArF-BAR protein (S3A Fig). The spherical liposomes transformed into an intense network of narrow tubules within 30 min of incubation with ArF-BAR and ArF-BAR^{mut2} (Fig 1D and 1E). This indicated that ArF-BAR was capable of robust liposome tubulation activity (S3B Fig). Additionally, the sequence alignment of the ArF-BAR with that of other characterized F-BAR domain-containing proteins, revealed the presence of positively charged conserved residues (S2 Fig). Earlier, In previous studies, the positively charged present in the F-BAR domains of CIP4 residues of FBP17 and Syndapin, have been shown to interact with negatively charged phospholipids to induce membrane curvature [28, 29]. Since ArF-BAR has conserved positively charged amino acids at positions 57, 58, 131 and 132 (S2 Fig), the role of these residues in membrane deformation were evaluated. This evaluation was performed by replacing the residues with glutamate (K57E, K58E, R131E and K132E) via site-directed mutagenesis, and the mutant version was referred to as ArF-BAR^{mut1} (S3C Fig) (S4B Fig). Predictably, As expected, it was

found that substituting the conserved lysine/arginine with glutamate abolished the tubulation activity of the F-BAR domain (Fig 1F). Protein kinase C1 (PKC1) proteins are ~~diversely~~ known to interact with diacylglycerol (DAG), which has a role in membrane interactions. To abolish the involvement of the unique ~~protein kinase~~ C1 domain of ArF-BAR in membrane deformation, ~~the~~ residues at positions W428 and L430 were replaced with ~~G428 and G430, respectively~~ glycine [30], and the mutant form was referred to as ArF-BAR^{mut2} (~~S3D~~ S4C Fig). Further, this mutated protein was used to assess the *in vitro* liposome tubulation activity, and a similar activity to that of the ~~native protein~~ ArF-BAR protein was found (Fig 1G). Thus, positively charged amino acids of F-BAR domain are involved in ArF-BAR mediated membrane tubulation (Fig 1H). To test whether the tubulation activity of ArF-BAR correlated with its lipid binding ability, a liposome co-sedimentation assay was used ~~(Fig 1H)~~ (Fig 1I). Compared to the native ArF-BAR protein, ArF-BAR^{mut1} showed reduced lipid sedimentation efficiency. In contrast, the sedimentation was unaffected with ArF-BAR^{mut2}. This indicated the importance of the direct interaction of the BAR domain with the lipids in liposome tubulation (Fig 1H and 1I). Together, these results suggest that F-BAR domain of ArF-BAR, plays a major role in interacting with the lipid membrane for binding and deformation.

ArF-BAR is required for *A. rabiei* virulence on chickpea

To elucidate the biological importance of *ArF-BAR* in ~~*A. rabiei* pathogenesis~~ fungal virulence, an *A. rabiei* knockout mutant (Δ*arf-bar*) was generated that lacked the entire protein coding region of *Arf-BAR* gene ~~was generated (Δ*arf-bar*)~~. The *ArF-BAR* gene was targeted for deletion using a homologous recombination approach. The results confirmed that the open reading frame (ORF) of *ArF-BAR* had been successfully replaced with a single copy of the hygromycin

Formatted: Font: Italic

Formatted: Font: Italic

Formatted: Font: Italic

resistance gene (*Hph*; ~~S4A~~S5A and ~~S4B~~S5B Fig). Simultaneously, Δ*arf-bar* mutant strain was complemented with a T-DNA cassette that expressed *ArF-BAR* under its own promoter (Δ*arf-bar/ArF-BAR*; S4~~B~~ and S5~~A~~ Fig).

To check the effect of *ArF-BAR* deletion on vegetative growth of *A. rabiei*; wild-type (WT), Δ*arf-bar*, and knockout complemented (Δ*arf-bar/ArF-BAR*) strains were grown on potato dextrose agar (PDA) plates. ~~Notably, the~~ T~~t~~he knockout mutants showed reduced radial growth compared to ~~wild-type *A. rabiei*~~ *A. rabiei* wild-type (WT) ~~(WT)~~ and this radial growth was restored in the Δ*arf-bar/ArF-BAR* ~~complementation mutant~~ (Fig 2A). To determine the *Ascochyta* blight (AB) disease development and virulence of the fungal strains, ~~an *in planta* infection bioassay was performed~~ the conidial suspensions of each strain were spray inoculated on susceptible chickpea variety plants individually. The ~~T~~typical AB disease symptoms, described previously [23,24], were observed on chickpea plants challenged with the *A. rabiei* wild-type (WT) and~~-~~ Δ*arf-bar/ArF-BAR*, but not on plants challenged with Δ*arf-bar* (Fig 2B). The degree of pathogenicity was ~~measured~~ quantified according to lesion number and lesion size, which were compared among the ~~*A. rabiei* WT~~ *A. rabiei* wild-type (WT) and mutants at 144 hpi. The lesion number per plant in Δ*arf-bar* was significantly lower than in the *A. rabiei* wild-type (WT) ~~-~~(~~WT,~~ Fig 2C). The mean lesion size was also much lower in Δ*arf-bar* than in the *A. rabiei* wild-type (WT) (~~WT~~, Fig 2~~d~~D). However, for the Δ*arf-bar/ArF-BAR* complementation mutant, the disease symptoms were comparable to those of the *A. rabiei* wild-type ~~(~~WT~~)~~. Overall, the disruption of the *ArF-BAR* gene significantly impaired the pathogenicity of *A. rabiei*.

To ascertain the role of ~~the evolutionary conservation of the F-BAR and unique PKC domains in contributing fungal pathogenicity,~~ F-BAR and unique C1 domains in fungal virulence, the two domains were independently inactivated by site-directed mutagenesis as

Formatted: Font: Italic

described earlier. The Δ*arf-bar* knockouts were complemented with residues mutated in F-BAR domain (Δ*arf-bar/ArF-BAR*^{mut1}) and ~~PKC~~ C1 domain~~s~~ (Δ*arf-bar/ArF-BAR*^{mut2}; S4B, S5B and S5C Fig). The ~~degree of pathogenicity~~ in virulence of Δ*arf-bar/ArF-BAR*^{mut1} strain was ~~lower than that of~~ very less as compared to the WT. Meanwhile, in the case of Δ*arf-bar/ArF-BAR*^{mut2}, the number of lesions per plant was significantly lower than in the WT, but there was no significant difference in the size of the lesions (Fig ~~2B,~~ 2C and 2D). The severity of the disease symptoms increased in the plants after 10 days post infection (dpi), but no significant differences were observed for plants challenged with Δ*arf-bar* strain (S6 Fig). Together, these results confirm that *ArF-BAR* is an important ~~pathogenicity~~ virulence determinant and that the positively charged F-BAR domain ~~is indispensable for fungal pathogenesis~~ residues are required for full-virulence of *A. rabiei.*

It was subsequently hypothesized that the ~~loss in pathogenicity of~~ reduced virulence in Δ*arf-bar* could have resulted from at least two factors: a) knockout mutants may have had a compromised ability to penetrate host tissue, or b) the reduced virulence ~~phenotype~~ of the mutant was a consequence of a reduction in fungal viability or inability to counter the host basal defense. To address these possibilities, ~~the depth of~~ hyphal penetration was examined and compared by inoculating ~~in~~ chickpea leaves ~~infected~~ with the WT and Δ*arf-bar* mutant strains ~~were examined and compared~~. The leaves of the chickpea plants challenged with respective strains were subjected to wheat germ agglutinin- Alexa Fluor™ conjugate (WGA-AF488) staining. At 48 hpi, the infected leaves were stained with WGA-AF488 to enable visualization of the fungus [31]. The infected leaves were optically sectioned using confocal microscopy starting from the surface of the leaves. It was observed that hyphae of the *A. rabiei* wild-type (WT) ~~wild type~~ strain efficiently penetrated into the chickpea leaves up to depths of

Formatted: Not Highlight

20.25 µm ± 3.92 (mean ± SD; n = 3) (S7A and S7C Fig). In stark contrast, the hyphae of the Δ*arf-bar* mutant were unable to penetrate beyond 9.83 µm ± 2.54 (mean ± SD; n = 3, S7~~A~~B and S7C Fig). Thus, the depth and efficiency of hyphal penetration by Δ*arf-bar* were significantly impaired ~~(S7B and S7C Fig)~~.

Further, to investigate the direct involvement of *ArF-BAR* in fungal viability under oxidative stress conditions, radial growth assays were performed for WT, Δ*arf-bar*, Δ*arf-bar/ArF-BAR*, Δ*arf-bar/ArF-BAR*^{mut1} and Δ*arf-bar/ArF-BAR*^{mut2} strains. These strains were inoculated on either potato dextrose agar (PDA) or PDA supplemented with menadione (250 µM and 500 µM) or 2 mM H_2O_2 ~~(2 mM)~~. The diameter of radial growth was analyzed at 10 dpi. The mycelial growth of Δ*arf-bar* was reduced compared to that of the WT. The mycelial growth was restored in the complemented Δ*arf-bar/ArF-BAR* and Δ*arf-bar/ArF-BAR*^{mut2} mutants (Fig 3A). However, the mycelial growth of complemented Δ*arf-bar/ArF-BAR*^{mut1} was unable to match that of the WT. Exposure to oxidative stress led to ~~a greater~~ further growth inhibition ~~rate~~ in Δ*arf-bar* ~~than in the WT (S8A and S8B Fig)~~(Fig 3). The involvement of *ArF-BAR* in oxidative stress tolerance was confirmed by the complementation ~~mutant~~, Δ*arf-bar/ArF-BAR*, which showed ~~similar~~ comparable resistance to that of the WT ~~(S8A and S8B)~~ (Fig 3). Further, exposure to oxidative stress led to greater growth inhibition in Δ*arf-bar/ArF-BAR*^{mut1} than in the wild-type. However, the growth inhibition of Δ*arf-bar/ArF-BAR*^{mut2} was similar to that of ~~WT~~ wild-type. The negative effect on growth of fungal strains till 10 dpi was more pronounced for H_2O_2 as compared to menadione (Fig 3; S8 Fig). ~~Consistent with the previous findings, t~~These results strongly support the hypothesis that *ArF-BAR* is ~~a positive regulator of pathogenicity in *A. rabiei*and~~ required for full virulence ~~the viability~~of *A. rabiei* wild-type (WT) on susceptible

Formatted: Font: Italic

chickpea host and are required for coping with the host basal defense, mainly oxidative ~~the fungus under~~ stress ~~conditions.~~

Absence of *ArF-BAR* ~~delays~~perturbs septa formation

Having a potential role in fungal virulence, ~~T~~the next aim was to determine the subcellular localization of ArF-BAR inside *A. rabiei* wild-type (WT) hyphal cells. In this regard, ~~an enhanced yellow fluroscent protein (EYFP) was used to create the fusion ,~~ expression of a chimeric protein, ArF-BAR::EYFP, which ~~transiently expressed in~~ was achieved by *Agrobacterium tumefaciens*-mediated transformation (ATMT) of Δ*arf-bar* strain [27]. The bioimaging of the ~~fluroresecently~~ enhanced yellow fluorescent protein tagged ArF-BAR showed punctate distribution throughout the cytoplasm of the fungal hyphae. The chimeric protein was mostly concentrated at the growing hyphal tip and at the septa (Fig ~~3~~4A and ~~3~~4B). Fungal hyphae transformed with chimeric ArF-BAR^{mut1}::EYFP exhibited disrupted localization of these punctate structures and the fluorescence was completely diffused throughout the cytoplasm. However, the distribution of the fluorescent puncta was unaffected in hyphae of strains stably ~~transiently~~ expressing ArF-BAR^{mut2}::EYFP (Fig 4A). In this strain, t~~T~~he punctate structures were distributed throughout the cytoplasm and were prominently concentrated at the growing hyphal tip (Fig 4~~3~~A). Further, to validate the spatiotemporal distribution pattern of the ArF-BAR protein during infection, fungal spores expressing ArF-BAR::EYFP were allowed to infect susceptible chickpea ~~leaves~~ stem peel. Microscopic observation of these ~~hyphae~~ stem peel infected ~~chickpea leaves~~ with fungal hyphae revealed a distribution pattern that was similar to that of the ArF-BAR::EYFP expressing hyphae growing on glass slides; the chimeric protein was predominantly distributed at the hyphal tip and septa (S9 Fig). The localization of ArF-BAR::EYFP towards the membrane of growing hyphae prompted us to check its localization with a Spitzenkörper marker

ArSNC1,which is a vesicle-bound v-SNARE protein and known for its role in polarized exocytosis in conjugation with other proteins [32,33,34]. The *A. rabiei* wild-type (WT) ArSNC1 was identified based on its high homology with the *M. oryzae* SNC1 (MGG_12614.6) encoded protein. However, The localization pattern of ArF-BAR::EYFP and ArSNC1::mCherry confirms the apical localization of ArF-BAR (Fig 4C). The delayed proliferation Δ*arf-bar* strain compared to that of the WT, on susceptible plant leaf surface and less penetration of host tissue (Fig 5A; S7 Fig) points towards mutant strain's defect in normal development or suppression of host defense. The *in vitro* germination and growth of WT and Δ*arf-bar* conidia, up to 12 h on hydrophobic surface, showed marginal difference (S10 Fig). However, the first cell from the growing hyphal tip in Δ*arf-bar* was observed to be elongated that may be due to delayed septation as ArF-BAR localizes to the septa. The arrangement of septa within the fungal hyphae of both the WT and the Δ*arf-bar* strain was examined in a number of growing fungal tips. Microscopic analysis of the Δ*arf-bar* mutant using calcofluor white, which precisely stains cell wall components, showed that the filaments hyphae lacked regularly spaced septa (Fig 5B). Interestingly, the distance of the first septum from the growing hyphal tip (polarized end) was significantly greater in the *Δarf-bar* mutant (58.5µm ± 4.65; mean ± SEM) than in the WT (23.5µm ± 3.23; mean ± SEM; Fig 3C and3D Fig 5C). It showed that Δ*arf-bar* mutant, as compared to *A. rabiei* wild-type (WT), has more length of first cell and second cell from the hyphal tip (Fig 5C). Overall, these results reveal that the ArF-BAr gene is necessary for appropriate fungal hyphal architecture, which is in turn, important for host penetration and virulence

Δ*arf-bar* impairs secretion of a candidate effector secretion

The substantial reduction in virulence of Δ*arf-bar* on chickpea has led us to hypothesize that the deletion mutant may have impaired in the effector secretion ability of *A. rabiei* wild-type (WT).

Formatted: Not Highlight

Formatted: Not Highlight

Formatted: Font: Not Italic

To further confirm the possible role of ArF-BAR in the effector secretion, a candidate secretory effector Ar93 (Accession number: GW996416) [26]~~, unpublished data)~~ possessing a classical signal peptide for secretion was ectopically expressed in WT and Δ*arf-bar* background. The secretion of Ar93 protein fused with EYFP::FLAG in media was examined using proteins from culture filtrate and cell lysates of WT and Δ*arf-bar*. The western blots analysis of culture filtrate and cell lysate of axenically grown stable fungal transformants (WT and Δ*arf-bar*) showed exclusive effector secretion in WT. However, the Ar93 was observed in cell lysates of both the WT and Δ*arf-bar* (Fig 5D). Thus, we conclude that Δ*arf-bar* mutant is impaired in secretion of Ar93 or similar effectors possessing classical secretory signal peptide.

~~ArF-BAR mediates early endosome biogenesis and endocytosis~~

Δ*arf-bar* mutant has impaired endocytosis

Since F-BAR domain proteins are known to ~~form~~ dimerize and form a canonical banana-shaped fold ~~and to dimerize~~ [35], ~~the dimerization of ArF-BAR was confirmed using a yeast two-hybrid (Y2H) system. The growth of yeast cells on selective media, and the expression of β-gal reporter gene suggested the diamerization of ArF-BAR protein.~~ This evidence further indicates~~d~~ the evolutionarily conserved nature of the BAR proteins (S1~~0~~1~~A~~ Fig). To ~~further~~ gain insight into the role played by ArF-BAR in endocytosis, an amphiphilic styryl type endocytic tracer dye, N-(3-triethylammoniumpropyl)-4-(p-diethyl-aminophenyl-hexatrienyl)pyridiniumdi bromide (FM4-64), was used ~~[36]~~[36]. The FM 4-64 staining of ~~Both~~ WT and Δ*arf-bar* ~~were stained,~~ and ~~the~~ its microscopic analysis revealed visible internal staining of the hyphae. The results suggested ~~that in WT hyphae, the dye was~~ rapid~~ly~~ internaliz~~ed~~ation of dye in WT hyphae, ~~In contrast,~~ compared ~~in~~ to *Δarf-bar* strain~~,~~ where no such internalization ~~was~~ observed~~,~~ even after

10–15 min of FM4-64 incubation (Fig ~~4A~~6A). To validate these findings, the mean fluorescence intensity area was determined in biological replicates (details in sub-section ‘quantification and statistical analysis’ of Materials and Methods).~~ ~~The result suggested that the endocytic activity from plasma membrane is impaired in Δ*arf-bar* (Fig 6B). The result provides evidence for the involvement of *ArF-BAR* in the endocytosis.

The central components of the endocytic pathway are the EEs, where the small GTPase Rab5 plays a major regulatory role in biogenesis [37]. *ArRab5*, an orthologue of *Rab5*, was identified in the *A. rabiei* genome ~~through NCBI blast searc,~~ using *Rab5* of *M. oryzae*~~,~~ and *U. maydis* as query at NCBI. The relationship between ArF-BAR-associated endocytosis and ArRab5-associated early endosomes was determined using a double-labeling experiment. *ArF-BAR* was tagged with *mCherry* and *ArRab5* with *EGFP*. This was followed by sequential transformation into the WT. The coalescence of fluorescence obtained showed perfect positive correlation between the two fusion proteins ~~(Fig 4B)~~ (Fig 6B).

Early endosomes mature to late endosomes followed by the replacement of Rab5 by Rab7 [38]. Thus, we aimed to determine whether the punctate distribution of ArF-BAR was associated with all endocytic vesicles or specifically to the EEs. In this context, similar to *Rab5* orthologue, an orthologue of *Rab7* was identified in *A. rabiei* and was tagged with EYFP (*ArRab7:EYFP*). A similar double-labeling experiment was performed that showed ~~that least correlation between the ArF-BAR::mCherry and ArRab7::EYFP fusion proteins (S10 Fig).~~ separate intensity and localization of ArF-BAR::mCherry and ArRab7::EYFP chimeric proteins (Fig 6C). In summary, these results uncover the role of ArF-BAR as ~~protein; they specifically bind to early endocytic vesicles, regulate the biogenesis of EEs, and mediate their motility during endocytosis.~~ it associates with early endocytic vesicle protein complexes and mediate endocytosis.

Formatted: Not Highlight

ArF-BAR modulates the actin cytoskeleton

The presence of SH3 (SRC homology 3) domains in F-BAR proteins is well documented for their relationship with the actin cytoskeleton via interactions with the Arp2/3 complex activator Wiskott-Aldrich syndrome protein (WASp) [39]. Since, ArF-BAR in *A. rabiei*, contains two consecutive SH3 domain at its C-terminu, we initially hypothesized for the probable existence of interactions of ArF-BAR with ArActin.. . Using Y2H system, it was shown that ArF-BAR does not interact directly with actin (Fig 75A), rather it physically interacts with WASp through its SH3 domains (572-760 amino acidFig 5B, S11A and S11B) (Amino acids 572-760; Fig 7B; S12A and S12B Fig). Further, to check the role of ArF-BAR in actin polymerization, a well-established *in vitro* actin polymerization assay was performed. The kinetics of actin polymerization was monitored by increase in the fluorescence of pyrene-labeled actin. The effect of purified ArF-BAR protein on actin nucleation (actin, Arp2/3 and WASp) was tested using a minimal set of components for all reactions. Interestingly, the addition of purified recombinant protein led to an increase in the actin polymerization rate (Fig 75C). By increasing the concentration of purified protein, a significant gradual activation in the rate of actin polymerization was observed (Fig 75C). These results strongly suggest that ArF-BAR plays an active role in WASp-dependent actin polymerization.

Appropriate organization of actin is required for vesicular dynamics, organelle movement and cytokinesis. Actin microfilaments or F-actin are organized into higher order structures comprising of actin patches, cables and rings that serve as the track for long distance transport [40]. To elucidate the relative importance of ArF-BAR in actin organization, the actin dynamics was were compared in the WT and Δ*arf-bar* mutants. Here, we took advantage of LifeAact, an

actin binding peptide fused with a fluorescence protein. LifeAact has been successfully employed for *in vivo* visualization of actin filaments and dynamics in a variety of organisms including fungi and plants [41]. In this study, a *LifeAact:mCherry* fusion construct was generated and transformed into both the WT and Δ*arf-bar* mutant to visualize cytoplasmic actin dynamics in fungal hyphae. Confocal microscopy revealed discrete actin patches and cables in the WT hyphae (Fig 5D),. In contrast, the while actin patches were rarely visible in the Δ*arf-bar* mutant (Fig 75D and 75E). Moreover, the actin cables were dramatically disorganized in the Δ*arf-bar* mutant. Although ArF-BAR does not directly interact with actin, it regulates actin polymerization and actin cytoskeleton through its association with WASp in the growing fungal hyphae.

ArCRZ1 is a potential transcriptional regulator of *ArF-BAR*

Thus far, the findings of the present study have revealed the importance of *ArF-BAR* in the regulation of endocytic pathways, which is crucial for the pathogenesis of *A. rabiei*. However, the transcriptional regulatory machinery associated with the endocytic pathway in filamentous pathogenic fungi is largely unknown. Hence, the transcriptional mechanism associated with the regulation of *ArF-BAR* expression in response to infection was subsequently analyzed. The *in-silico* bBinding motifs for fungal (TFs) were identified in the upstream regulatory sequences of *ArF-BAR*. The YEASTRACT online tool [42] revealed seven different putative TF binding sites were identified (S1 Table) in 1 kb promoter region of *ArF-BAR* gene. Among these, three binding sites were found for calcineurin-responsive zinc finger transcription factor 1 (CRZ1; Fig 6A), positioned at -106, -136, and -254 upstream toof the ArF-BAR translational start site ATG (Fig 8A) promoter, were found for calcineurin-responsive zinc finger transcription factor 1 (CRZ1; Fig 6A).

CRZ1 is an evolutionarily conserved TF from yeast to mammals. CRZ1 was chosen for analysis because it regulates the expression of various genes involved in stress tolerance [43] and is widely known to translocate inside the nucleus with an increase in cytosolic Ca^{2+} ion concentration [44]. *Ascochyta rabiei* ~~CRZ1 of~~ ~~*A. rabiei*~~ *gene* (*ArCRZ1*; ST47_g3738) encodes a protein that possesses a serine-rich region (SRR), two consecutive calcineurin docking domains (CDD), characterized by PxIxIT motif (PRILPQ and PEINID) and a single C_2H_2 zinc finger motif (S13~~2~~A Fig). To determine the role of ArCRZ1 in the transcriptional regulation of *ArF-BAR*, the binding of ArCRZ1 to the regulatory sequences of *ArF-BAR* was confirmed. This confirmation was performed via an electrophoretic mobility shift assay (EMSA) using recombinant His-tagged ArCRZ1 proteins. Shifting was observed for the DNA fragment possessing the calcineurin-dependent response element (CDRE) in the presence of purified His-ArCRZ1. However, mutations in this CDRE resulted in the abolishment of binding (Fig ~~6~~8B and ~~6~~8C).

Subsequently, the sub-cellular localization of ArCRZ1 under Ca^{2+} and oxidative stress conditions was determined. The ~~WT~~ *A. rabiei* wild-type (WT) was transformed with a translational ~~of~~ fusion of *ArCRZ1* with *EYFP* towards C-terminus. After, 0.2 M $CaCl_2$ treatment, ~~C~~confocal microscopy revealed ~~the elegantly concentrated~~ nuclear localization of the ArCRZ1::EYFP signal ~~under stress conditions~~ (Fig 8~~6~~D). Interestingly, in absence of any treatment, the ArCRZ1::EYFP fusion protein was uniformly distributed within the cytoplasm during normal growth on glass slides (Fig 8D). Since, the nuclear translocation of ~~Ar~~CRZ1 is mediated by a phosphatase (calcineurin) [45], a chemical genetics approach was used to confirm the relationship between calcineurin and ArCRZ1. ~~Here, the immunosuppressant~~ A potent calcineurin inhibitor, FK506 (Tacrolimus) was used to silence the enzymatic activity of

calcineurin [43]~~[43]~~. FK506 potentially inhibited nuclear translocation of ArCRZ1::EYFP, which ~~indicates~~ suggests that calcineurin plays a role in the nuclear translocation of ArCRZ1 under stress conditions (Fig ~~6~~8D).

To corroborate and extend these findings during infection, susceptible plants were challenged with fungal conidia expressing ArCRZ1::EYFP and nuclear localization chimeric protein was observed ~~(S12B and S12C Fig)~~(Fig 8E). Overall, chemical genetic results confirm the evolutionarily conserved regulation of ArCRZ1 by calcineurin under stress conditions.

To uncover the transcriptional regulation of the *ArF-BAR* gene mediated by ArCRZ1, a targeted deletion of the *ArCRZ1* gene was generated (Δ*arcrz1*), followed by complementation of mutant with *ArCRZ1* controlled by native promoter (Δ*arcrz1/ArCRZ1*; S13~~2~~C,-S1~~2~~3D Fig). Interestingly, no significant difference in radial growth diameter was observed between Δ*arcrz1* mutant and ~~WT~~ *A. rabiei* wild-type (WT) (Fig 7A). The expression pattern of *ArF-BAR* in the WT and Δ*arcrz1* was analyzed using semi-quantitative RT-PCR. The results clearly showed significant reduction in *ArF-BAR* ~~gene expression~~ transcription in the *Δarcrz1* mutant (Fig 9B). Imposing oxidative stress to both the WT and the Δ*arcrz1* mutant via menadione treatment revealed an upregulation of the *ArF-BAR* transcript in the wild-type. However, ~~T~~this upregulation was found to be completely abolished in the Δ*arcrz1* mutant (Fig ~~7~~9B). Overall, these results substantiate that under oxidative stress condition, the ~~novel~~ transcriptional regulation of *ArF-BAR* gene ~~expression~~ is mediated by the activated ~~TF~~ ArCRZ1.

Formatted: Font: Italic

Loss-of-function of ArCRZ1 compromises virulence similar to *Δarf-bar*

~~As ArCRZ1 transcriptionally regulates the expression of ArF-BAR,~~ As *ArF-BAR* gene was transcriptionally regulated by ArCRZ1, it was hypothesized that the pathogenicity phenotypes of

Δ*arcrz1* should be similar to those of the Δ*arf-bar* mutants. Consistent with this hypothesis, it was observed that *Δarcrz1* mutants displayed compromised virulence during chickpea infection bioassay. Results showed that t~~T~~he number and size of lesions were lower in the Δ*arcrz1* mutant than in the *A. rabiei* wild-type (WT)~~WT~~. This virulence~~pathogenicity~~ defect was rescued in Δ*arcrz1/ArCRZ1* complementation strain (Fig 9~~7~~C–9~~7~~E). The radial growth patterns of Δ*arcrz1* mutants grown on PDA supplemented with Ca^{2+}, menadione and sodium dodecyl sulfate (SDS) were also monitored. Under these tested stress conditions, the growth inhibition was significantly greater for Δ*arcrz1* than for *A. rabiei* wild-type (WT)~~WT~~. However, the growth phenotype was restored in the Δ*arcrz1/ArCRZ1* strain, which suggests that *ArCRZ1* plays a crucial role in calcium ion signaling, oxidative stress response and maintaining cell-wall integrity during infection (~~S13~~ S14 Fig). Taken together,~~These results~~ infection assay and stress treatments demonstrate that ArCRZ1 is a key regulator of the ArF-BAR,~~endocytic process.~~ which further regulates various cellular processes like endocytosis, effector secretion and promotes fungal survival under host generated stress condition. ArCRZ1 induces the expression of *ArF-BAR* by directly binding to its ~~gene promoter~~ *cis*-regulatory region and is critical for *ArF-BAR* dependent virulence.

Discussion

Dynamic membrane remodeling is essential for maintaining the integrity and identity of cells and cellular compartments [40]. Biological macromolecules, such as BAR superfamily proteins, can sense or induce membrane curvature. They are well-known modulators of transient membrane deformation [39]. Emerging evidence strongly suggests that EEs are crucial for long-distance intracellular communication. Thus, EEs and their roles have broad implications for a wealth of

cellular processes such as growth, development, and virulence in filamentous fungi [4,46,13]. The results of the present study further corroborate this conclusion and provide new information regarding signaling and transcriptional control of F-BAR proteins in phytopathogenic fungi. To date, our understanding of the role played by F-BAR proteins in membrane curvature generation and efficient long-range endosome trafficking in fungi during plant-pathogen interactions remains very limited.

~~This study unravels the intrinsic mechanism of EE formation in phytopathogenic fungi during polarized hyphal tip growth and host penetration.~~ To ~~the~~ best of our knowledge, this study provides the ~~first~~ evidence that an F-BAR domain protein ~~can~~ act as a key mandate for virulence of a phytopathogenic fungi~~pathogenicity~~. ~~In phytopathogenic fungi, *Ar*F-BAR navigates endosome trafficking via the coordinated action of cell membrane remodeling and actin reorganization. The present findings suggest a model in which *Ar*F-BAR modulates the invagination step and recruits the apical plasma membrane for EE formation during filamentous growth and host penetration of *A. rabiei*.~~ Based on the evidences provided in the present study, we propose that ArF-BAR performs ~~two different but well-coordinated functions~~ events that result in ~~EE formation~~endocytosis, effector secretion, septa formation and actin dynamics during the *A. rabiei* hyphal growth (Fig 10~~8~~).

In a first set of evidences, our results indicate the role of membrane remodeling in septa formation and hyphal tip growth. Previous studies, largely conducted in animal models, have highlighted that the F-BAR domain is a membrane-deforming module and is involved in endocytosis [47,29]. The endocytic event is crucial for the uptake of signal cues and nutrition from the host, and aids apical recycling of membrane receptors and proteins. This process thus helps to maintain the overall polarity of the hyphae that is required for fungal growth and

Formatted: Font: Not Italic

virulence [6,43]. The generation of EEs and their trafficking involves microtubule dynamics, actin cytoskeleton rearrangements and most importantly, extensive membrane remodeling [48]. Actin dynamics and microtubule organization have been extensively studied. However, the detailed mechanism underlying the functional regulation of fungal EE biogenesis and EE trafficking at the hyphal tip during plant pathogenesis remains poorly understood.

The present study identifies ~~an unprecedented~~the role for the ArF-BAR protein as an essential component of endocytosis ~~that positively regulates EE biogenesis~~. Unveiling other networks associated with this system, ArF-BAR in coordination with Arp2/3-WASp, was found to mediate actin cytoskeleton assembly at the hyphal tip. The Arp2/3-WASp assembly is a prerequisite for host penetration [33, 49]. The distribution of the F-actin network, which serves as the molecular track for endosome trafficking [8,39], was disorganized in the ~~*Δ*~~Δ*arf-bar* mutant, affecting endocytic transport.

Formatted: Font: Not Italic

Th~~is phenomenon would explain why the~~e loss of function mutant of the ArF-BAR function leads to the substantial attenuation of virulence compared to WT. This loss in virulence is similar to that observed in the rice blast fungus *M. oryzae* and *U. maydis* where endocytosis is crucial for the recognition of host partners during the early stages of pathogenic development [7]. However, the slow growth of the mutant (Fig 3 and S8 Fig) may be argued for a possible cause of the reduced virulence by itself. We observed that the symptoms in plant bioassay (Fig2 and 6 Fig) are severe in case of WT. The Δ*arf-bar* mutant showed only small or almos no lesions on plants. These observations clearly indicated that such severity not mere due to slow growth of hyphae. Therefore, we hypothesized that the knock-out mutant may have impaired in ability to secrete effectors of *A. rabiei*. Fungal pathogens secrete an array effector protein to counter host immunity and promote infection process [50]. Δ*arf-bar* mutant strain was found defective in

Formatted: Font: Not Italic

Formatted: Font: Not Italic

secretion of a secretary signal sequence containing effector, Ar93 (Fig 5D). This experiment clearly shows that cumulative effect of both the slow growth and effector secretion are the primary cause of severe virulence defects in Δ*arf-bar* mutant. Additionally, some of the fungal effectors can change the structural and functional conformations as well as the localization of host target proteins that are key factors in host ROS production [51]. Pathogen survival under host-generated ROS is important for fungal growth and virulence. We also observed high transcript accumulation of under oxidative stress conditions (Fig 1B). However, it will be interesting to decipher the entire mechanism of pathogen survival correlating all the components together.

Therefore, we conclude that ArF-BAR proteins act as membrane-bound tethers to properly recruit the apical plasma membrane for EE formation during endosome trafficking in *A. rabiei*.

ADs discussed above, during host-pathogen interactions, the key to successful pathogenesis is to overcome the rigid defense responses of the host. Collectively, the first challenge the pathogen encounters is an oxidative burst at the site of infection, which initiates various signaling cascades in the pathogen that aid its survival. Calcium, an essential secondary messenger, mediates one such signaling cascade [52]. In response to stimuli, the cytosolic Ca^{2+} concentration increases [53] and modulates various Ca^{2+}-binding proteins such as calmodulin. The Ca^{2+} and calmodulin complex activates calcineurin. In many eukaryotes, calcineurin is known to regulate the activity of CRZ1, which is usually localized in the cytosol in phosphorylated form. Upon activation, CRZ1 gets relocated to the nucleus. In pathogenic fungi, many of the CRZ1-dependent targets, such as those involved in the maintenance of cell wall integrity, thermo-tolerance, cation homeostasis, azole tolerance and hyphal growth have been identified [54]. However, in the present study *ArF-BAR* was identified as a novel ArCRZ1target. Here, our study assumes

Formatted: Font: Not Italic

functional significance for plant infection as we identified an F-BAR protein that is regulated by transcriptional network. We have identified the calcium-regulated CRZ1 transcription factor that regulates the transcription of F-BAR. These results,~~The identification of *ArF-BAR* as a target~~ improves our understanding of the novel regulatory mechanism of endocytosis in filamentous fungi, where *ArF-BAR* functions downstream of ArCRZ1.

The development of septa is an important event during hyphal differentiation that is required for the formation of sexual structures and asexual spores [53]. Septation is comparable to cytokinesis that additionally includes cell separation. A cascade of events is involved in septum biogenesis, which includes assembly of the contractile actomyosin ring (CAR), plasma membrane ingression and cell wall constriction [55]. In fission yeast, Cdc15 and Imp2, and in budding yeast, Hof1 are the major F-BAR domain-containing proteins implicated in the formation of the CAR, and the primary and secondary septa during cytokinesis [56,57]. Additionally, Cdc15 of *U. maydis* also implicated for similar phenotype [16]. Therefore, the localization of ArF-BAR along the septum ring indicates that endocytosis is one of the pathways responsible for regulating the development of the septa. Septation is initiated at the definitive size of the hyphae [58]. With the initial recognition and establishment of disease in the host, the pathogen needs to proliferate at an enormously increased rate. Therefore, maintaining proper hyphal architecture and polarity is fundamental for pathogenesis that demands rapid coordinated internalization and recycling events [59]. T~~T~~he fungal mutant Δ*arf-bar*, deficient in ArF-BAR protein, ~~displayed~~ revealed~~a~~ delay in septa~~um~~ formation and displayed decreased virulence in the absence of proper hyphal structure. ~~*In silico* analysis of F-BAR proteins from various filamentous fungi, revealed the evolution of the conserved protein kinase C1 domain. The PKC proteins are phospholipid- and DAG-dependent kinases involved in various intracellular signaling cascades. Fungal PKC has a~~

Formatted: Font: Italic

Formatted: Font: Italic

Formatted: Font: Not Italic

~~serine/threonine kinase domain. In *Saccharomy cescerevisae* and *S. pombe*, the PKC is known to activate MAP kinase signaling that helps in cell wall damage repair and in *C. albicans*, PKC helps to provide osmotic tolerance [54]. Preliminary studies from other filamentous pathogens, such as *Aspergillus fumigates* and *Neurosspora crassa*, suggest the involvement of PKC proteins in cellular integrity maintenance. PKC family members have three regulatory domains C1, C2 and HR1, where the C1 domain of classic PKC enzymes has the ability to bind to DAG and phorbol esters [55]. Studies have reported the presence of two different cysteine-rich (C1) motifs, C1A and C1B, termed as "typical" and "atypical," respectively, depending on whether they "do" or "do not" bind to DAG and phorbol esters [57]. Interestingly, the presence of a specific C1 domain provides uniqueness to the F-BAR protein of filamentous fungi. However, in the current study, the targeted mutation of this domain did not have any functional relevance for virulence. This lack of relevance may have been for two reasons; a) C1 might be present in an "atypical" form or b) the lack of the C2 domain in ArF-BAR; both the C1 and C2 domains are required for full-enzymatic activation of PKC [58].~~

In summary, we propose that the ArF-BAR protein of *A. rabiei* has the potential to interactively affect the hyphal growth and ~~pathogenic~~ virulence ~~developmental trajectories~~ of filamentous fungi. This evolving model provides mechanistic insight into the role of a membrane scaffolding protein in the process of endosome trafficking in fungal pathogenesis. In turn, this provides many additional potential targets for the development of effective and durable strategies to control AB fungal disease. Therefore, the observations of the present study in context to the intracellular trafficking, during the early stages of plant-pathogen interactions, have broad relevance for shaping disease-control strategies. These findings may also be helpful for the disease control of animal-infecting fungal pathogens such as *A. fumigatus* and *C. albicans*. Thus, further studies

will be directed to characterize the interacting partners influenced by ArF-BAR during endosome formation. ~~A diverse spectrum of studies has revealed the existence of two parallel independent endocytic mechanisms: a) clathrin-mediated and b) clathrin-independent [59]. Similarly, extensive studies will be required to fully elucidate the ArF-BAR-mediated endocytic mechanism. Such studies would help us to understand the complex interplay underlying the endosome formation required for fungal virulence.~~ Further, the understanding of its regulatory mechanism would help in scrutinizing the molecular and cellular basis of disease development, which would subsequently help develop disease-control strategies for filamentous fungi.

Field Code Changed

Materials and Methods

Fungal strains and growth conditions

A virulent isolate of *Ascochyta rabiei* (ArD2; ITCC No. 4638) was procured from IARI, New Delhi. The single spore culture of this mating type 2 isolate was generated and maintained as wild type fungi for research work. The WT and its derivative fungal strains (S2 Table) were maintained on potato dextrose agar (PDA; Difco Laboratories, pH 5.2-5.5) at 22ºC for 10-15 days to assess the growth pattern and colony characteristics [26]. The fungus was routinely subcultured on the PDA plates supplemented with chickpea extract to maintain virulence. To determine the vegetative growth pattern of fungal mycelia in response to oxidative stress, PDA plates supplemented with menadione (250 µM and 500 µM; Sigma-Aldrich, USA) and H_2O_2 (2 mM; Sigma-Aldrich, USA) were used. The conidial suspensions of 10 µl ($1x10^3$ conidia/mL~~l~~) were inoculated at the centre of PDA plates for growth assay. After 10 days of incubation under optimum growth conditions, the diameters of fungal colonies were measured using ImageJ software. Three independent biological experiments were performed with three technical replicates each time.

RNA extraction and expression analysis

Total RNA was extracted from 5-6 days old fungal mycelia grown in potato dextrose broth (PDB; Difco Laboratories, USA) or from plant tissues inoculated with *A. rabiei* wild-type (WT)~~WT~~ using TRIzol reagent (Invitrogen, USA). The isolated total RNA was subjected to DNase1 (Promega, USA) treatment and subsequently used for first-strand cDNA synthesis using SuperScript IV reverse transcriptase (ThermoFisher Scientific). Targeted gene expression was determined by qRT-PCR with ABI7900 (Applied Biosystems, USA), using SYBR Green PCR master mix (Applied Biosystems, USA). Relative expression of fungal genes was calculated after normalization~~ed~~ with elongation factor α (*ArEFα*; ST47_g4052) using this $2^{-\Delta\Delta ct}$ method [60]. The data were analysed from three biological replicates each having three technical replicates.

Site-directed mutagenesis

The mutations at ~~requisite~~ required sites of *ArF-BAR* were achieved by PCR amplification of pET28a(+):*ArF-BAR* clone with pre-designed primers containing mutations of interest. Mutagenesis was performed using the QuikChange II Site-directed mutagenesis kit (Agilent, USA). Web-based Quik Change Primer Design tool (www.agilent.com/genomics/qcpd) was used to design primers. The presence of mutations in clones was confirmed by Sanger sequencing.

Targeted gene knockout and complementation in *A. rabiei*

Homologous gene replacement with *hph* cassette strategy was used to generate knockout (KO) constructs for *A. rabiei* genes. Genomic DNA was isolated from 5-day grown PDB culture of *A. rabiei* using GenElute~~TM~~ Plant Genomic DNA miniprep kit (Sigma-Aldrich, USA). The 5′ flanking genomic sequences of *ArF-BAR* were amplified from *A. rabiei* genomic DNA using primer pairs of ArF-BARKOif5F and ArF-BARKOif5R while the 3′ flanking sequences were

amplified using Ar72KO3 and Ar72KO4 (S3 Table). These amplified 5′ and 3′ flanking sequences were cloned sequentially into pGKO2 vector at *Kpn*I/*Pst*I and *Bam*HI/*Eco*RI sites, respectively. The cloned *ArF-BAR* gene replacement cassette of ~3.4 kb, including 5′ and 3′ flanking sequence along with *hph*, was amplified using primer pair ArF-BARKOif5F and Ar72KO4, and transformed into *A. rabiei* protoplasts. The *A. rabiei* protoplast transfection was performed as described earlier [61] , with some minor modifications. The putative transformants were selected on a PDA plate supplemented with 50 µg/µl hygromycin. To generate the complementation constructs of *ArF-BAR* and its mutated (mut1 and mut2) versions, about 4.4 kb DNA fragment having the *ArF-BAR* native promoter, ORF region, and TrpC terminator was amplified and cloned into pBIF2 vector (Bacterial selection- kanamycin; Fungal selection- G418) at *Eco*RI and *Hind*III sites. These three constructs of *ArF-BAR* were independently transformed into *Δarf-bar* mutant strain by ~~ATMT~~ *Agrobacterium tumefaciens*-mediated transformation (ATMT) ~~—~~[27]. Similar~~ly~~, to generation of ArF-BAR knockout and complementation mutant, *A. rabiei ArCRZ1* gene knockout mutant and complementation strains were also developed. The knockout primers used for the same enlisted in S3 Table.

Gene knockout confirmation by PCR and Southern blot

The single spore culture of putative KOs selected on hygromycin was initially screened by genomic PCR. A primer ~~set~~ binding at position 5′ to the homologous recombination region and TrpC promoter was used for gene upstream integrity check~~region~~ while a primer ~~set~~ binding at *Hph* gene and position 3′ to the homologous recombination region was used for gene downstream integrity check (S3 Table). The putative transformants for complementation strains were also screened by genomic PCR. ~~The complemented strains single spore culture was also confirmed by genomic PCR.~~ The PCR positive *Δarf-bar*, and *Δarcrz1* mutants (KOs) and

Formatted: Font: Italic, Not Highlight

Formatted: Not Highlight

Formatted: Not Highlight

complementation~~ed~~ strains were further verified by Southern blot. The genomic DNA of WT, *Δarf-bar* and *Δarcrz1* mutants was digested with *Eco*RI enzyme while genomic DNA was digested with *Eco*RI and *Hind*III for the complementation~~ed~~ ~~mutant~~ strains (*Δarf-bar/ArF-BAR*, *Δarf-bar/ArF-BAR*^{mut1}, *Δarf-bar/ArF-BAR*^{mut2}, and *Δarcrz1/ArCRZ1*). The digested DNA was separated along with λ DNA/*Hind*III marker (ThermoFisher Scientific) and blotted to a membrane followed by hybridization with radioactive probe ~~[57]~~ prepared using the random primers ~~labeling~~labeling NEBlot® kit (New England Biolabs, USA). The band detection was carried out using Typhoon® phosphor imager (GE Healthcare, USA).

Plant infection assay

Two-week-old susceptible chickpea (Pusa 362) plants grown in plant growth chambers under controlled conditions (D/N temperature: 24ºC/18ºC; Relative Humidity: 80%; light intensity 250 µE/m^2/s; D/N light duration: 14/10 h) were used for infection assays. Conidial suspensions of *A. rabiei* strains were collected separately from a 20-days-old PDA plate grown culture. Two-weeks-old plants were spray inoculated with conidial suspensions diluted to 2x10^6 conidia/mL~~l~~. Plants were again kept under optimum conditions. Disease lesions were examined 5-7 days after spray inoculation.

***In vitro* Protein purification**

Respective cDNAs were cloned in pET28a (+) and transformed into *E. coli* BL21-CodonPlus (DE3)-RIPL cells. ~~The p~~Protein expression was induced with 0.5 mM Isopropyl β-D-thiogalactoside (IPTG) for 6 h at 23ºC. Bacterial pellet was lysed in buffer [500 mMNaCl, 50 mM $NaPO_4$ (pH 8.0), 10 mM Imidazole, 1mM β-Mercaptoethanol, 1 mg/mL~~l~~ lysozyme and 1mM Phenylmethylsulfonyl fluoride (PMSF)] by incubating 30 min on ice, followed by sonication (10 cycle, 70% amplitute). The cell lysates were precipitated followed by 0.45 µm

filtration. The cleared lysate was incubated with Ni-NTA resin for 30 min at 4ºC. The His-tagged fusion proteins was purified using a Ni-NTA column (Bio-Rad, USA). Proteins were~~as~~ eluted in elution buffer [500 mMNaCl, 50 mM $NaPO_4$ (pH 8.0), 10% glycerol and 200 mM imidazole]. The quality and quantity of eluted protein were checked by SDS/PAGE and Bradford assay, respectively. The proteins were dialyzed in respective compatible buffers followed by concentration.

Liposome preparation and Tubulation assay

Liposomes were prepared as described previously [63] using 70% Phosphatidylethanolamine (POPE), 20% Phosphatidylcholine (POPC), and 10% Rhodamine B-conjugated PE (Echelon Biosciences, USA). In an amber vials, all the lipids were initially dissolved in chloroform: methanol (65:35;v/v) mixture and vials were kept under liquid nitrogen for 10 min before being immediately subjected to vacuum desiccation/lyophilisation for 2 h at 60 mTorr. The lipids were hydrated with buffer [25mM Tris-HCl (pH 6.8) and 100mM NaCl] and subjected to three freeze-thaw cycles of 5 min each at 68 ºC and liquid nitrogen. Extrusion was performed at 68 ºC on a pre-heated mini extruder (Avanti Polar Lipids, USA). The prepared liposomes were diluted as desired and immediately proceeded for tubulation assay. Before use, the purified proteins were subjected to 100,000 g centrifugation for 20 min at 4 ºC to remove the aggregates~~ proteins~~. To examine tubule formation, the mixed liposome and protein samples were analysed in Lumox® 24-well plate (Millipore, USA) by live-cell imaging on Axio Examiner.Z1 (Zeiss microscope).

Liposome co-sedimentation assay

The purified fusion proteins were pre-centrifuged, before the assay, at 100,000 g for 15 min to remove protein aggregates. Protein from the supernatant was mixed with freshly prepared synthetic liposomes with gentle tapping. Ultracentrifugation was performed at 100,000 g for 15

min at 4ºC and the supernatant and the pellet were carefully separated. The supernatant was mixed with 1:1 loading buffer while the pellet was re-suspended in 2x loading buffer. Samples were analysed on Coomassie stained SDS-PAGE.

Yeast two-hybrid assays

The interactions of various combinations between ArF-BAR, ArActin, ArWASP, and ArF-BAR domains in yeast cytoplasm were examined using the split-ubiquitin based DUALhunter system (Dualsystems Biotech). The ORFs were cloned at *Not*I and *Asc*I restriction sites or by LR clonase II into pGDHB1 and pGPR3-N vectors. The cloned plasmids were co-transformed along with necessary controls into NMY51 strain using the EZ- Yeast transformation kit (MP Biomedicals, USA) and plated on SD- L/- W plates. The plating of yeast clones on required synthetic media to check protein-protein interactions in yeast was done as described previously [22]. All the interactions were verified by three independent experiments.

Effector secretion assay

For total fungal protein extraction, fungal mycelia were grown in PDB at 22˚C, 120 rpm for 7 days and harvested by filtering through three layers of sterile Mira cloth (EMD Millipore Corp, Germany). Tissues were snap freezed in liquid Nitrogen and stored for future use. The mycelial tissue were grounded to fine powder and resuspended in Tris-glycine buffer pH 8.3 (3g Trizma, Sigma-Aldrich, USA and 14.4 g Glycine, Sigma-Aldrich, USA in1Lit MQ). Resuspension were then centrifuged at 16 x 1000g for 40 min at 4˚C. The supernatant or cell lysate were collected and the total protein was separated on SDS PAGE followed by immune blot with anti-Flag Ab (Cohesion biosciences, Singapore). For the extraction of secreted proteins, 7 days old grown mycelia in PDB was treated with 250 µM menadione (Sigma-Aldrich, USA), to mimic host induced oxidative stress condition. Axenic culture filtrate (CF) was collected and filtered through

Formatted: Space After: 8 pt

three layered Mira cloth (EMD Millipore Corp, Germany) to separate fungal mycelia. Further, CF was sequentially filtered with 0.45 and 0.22 μm Durapore PVDF membrane filters (Sigma-Aldrich, USA). Filtered CF was then concentrated using 3kDa Amicon® Ultra-15 Centrifugal Filter Units (Merck, USA). Secreted proteins in concentrate were then separated on SDS-PAGE followed by immunoblot with anti-Flag Ab.

Formatted: Font: Bold

Actin polymerization assay

The actin polymerization modulation activity of the proteins was checked using Actin Polymerization Biochem Kit (Cytoskeleton, USA), using the manufacturer's instruction. Freshly solubilized components-; 13 nM Arp2/3 protein complex and 15 nMWASp-VCA domain-GST purified (Cytoskeleton, USA) along with freshly purified ArF-BAR protein in concentrations of 200, 400 and 600 nM were used. The reaction was carried out in an opaque 96-well plate and kept in the dark. The actin polymerization rate was recorded by monitoring the pyrene fluorescence signals using CLARIO® star plate reader (BMG Labtech, Germany) with the following settings; slow kinetics, 60 sec interval, λ_{ex} = 360±15 nm and λ_{em} = 420±20 nm.

Electrophoretic mobility shift assay (EMSA)

Formatted: Left, Space After: 0 pt, Line spacing: single

His-tagged ArCRZ1 (*His-ArCRZ1*) expression construct was developed generated and recombinant protein was purified. The wild type and mutated CDRE DNA fragments were assembled by annealing oligonucleotide pairs in a thermal cycler by heating at 95º C for 5 min followed by cooling at RT for 15 min. End labelling of DNA fragments was performed by [γ^{32}P] dATP (Board of Radiation & Isotope Technologies, BRIT, India), and Polynucleotide Kinase (Thermo Fisher Scientific, USA). Additionally, for competition assays, two complementary oligonucleotides were annealed at equimolar concentration. Purified His-ArCRZ1 protein was

Formatted: Font: (Default) Arial, Not Bold, Underline, Font color: Custom Color(RGB(26,13,171)), Pattern: Clear (White)

incubated with 10 ng of labelled DNA fragment in the presence of 1 mg of poly-deoxy-inosinic-deoxy-cytidylic acid [poly (dI-dC)] and 1X binding buffer (15mM HEPES (pH 7.6), 0.2 mM $MgCl_2$, 35 mMKCl, 1 mM DTT and 1% glycerol) in a reaction volume of 30 µl for 25 min at room temperature. DNA loading dye was used to terminate the reaction. The competitive assays were performed using 50, 100 and 200 times of specific fragments in excess. To identify the relative binding, the complexes were resolved on 6% native PAGE, dried, and autoradiographed on X-ray films.

Microscopic methods

Sample preparation for microscopic analysis

The conidia from the respective fungal strains were harvested in 1 mL~~l~~ sterile distilled water from 15-day-old fungal mycelia grown on PDA plates. The conidial suspension was filtered through Mira cloth. Ten microlitres (1×10^6 conidia/mL~~l~~) of suspension was kept on a sterile glass coverslip and allowed to grow under the optimal conditions for 24 h in dark. To investigate the localization of chimeric proteins in fungal hyphae, growing on chickpea stem peel, the conidia were allowed to grow for 36 h under optimum conditions of infection. The grown hyphae samples were then used for confocal laser scanning microscopy.

Confocal laser scanning microscopy

For microscopic studies, TCS SP5 and TCS SP8 confocal laser scanning microscopes (Leica Microsystems, Germany) were used. For subcellular localization, ~~the~~ conidia were harvested from transgenic fungal strains expressing fluorescent-tagged proteins. The Z-stacked images with 1 µm step size were acquired using a high-resolution CCD camera. For calcofluor-white

(CW), the hyphae grown on the glass slide were incubated for 10 min in the CW solution (Sigma-Aldrich, USA). After incubation, the stained hyphae were rinsed with PBS (pH 7.4) followed by rinsing with sterile distilled water, before image acquisition. For FM4-64 uptake, 1 mL~~l~~ of harvested conidial suspension was centrifuged at 2,500 g, washed twice with sterile distilled water and then allowed to germinate on glass slides. The aqueous solution (10 µM) of FM4-64 dye (Invitrogen, USA) was added directly to the fungal mycelia. After 10 min incubation, FM4-64 dye was rinsed from slides thoroughly before imaging fungal hyphae. Images were captured with a~~in~~ TCS SP8.

For host penetration assay, infected chickpea leaves (WT and Δ*arf-bar*) were placed in 100 % ethanol for 48 h to undergo bleaching for the complete removal of chlorophyll. Subsequently, leaves were incubated for 4 h in 10 % KOH at RT followed by washing 4-5 times in Phosphate buffer saline (PBS, pH 7.4). The processed leaves were then stained with chitin specific dye WGA-AF 488 (Invitrogen, USA). The leaf samples were rinsed in PBS (pH 7.5) before microscopic visualization. Confocal images were captured with a~~on~~ a TCS SP5 confocal microscope.

For sub-cellular localization of ArCRZ1::EYFP under oxidative stress conditions, fungal conidia expressing chimeric protein~~ expressing fungal conidia~~ were isolated and allowed to grow for 12 h. The hyphae were exposed to $CaCl_2$ (200 mM, Sigma-Aldrich, USA) for 1 min prior to microscopy. To assess the involvement of calcineurin in nuclear localization of ArCRZ1::EYFP, the hyphae were~~as~~ exposed to 5 µg/µL~~l~~ FK506 (Sigma-Aldrich, USA), 5 min prior to the addition of $CaCl_2$. The confocal images were acquired with a~~in~~ TCS SP5.

For subcellular localization of ArCRZ1::EYFP during infection, susceptible plants were challenged with conidia expressing ArCRZ1::EYFP. The images were acquired after 48 h using TCS SP5.

Quantification and Statistical analysis

Quantification analysis of relative fluorescent intensity, lesion size, radial diameter, the distance of septa from the hyphal tip ~~and distance travelled~~ were analysed by ImageJ/Fiji software. To calculate the significance of means/differences between two groups, Student's t-test and oneway ANOVA followed by Tukey test between multiple groups were performed using GraphPad Prism 6. Significance was accepted at $p < 0.05$, as noted in the text of legends. Replicates are indicated in the legends.

Bioinformatic analysis

All the gene and protein sequences were acquired from NCBI server (http://www.ncbi.nlm.nih.gov). Multiple sequence alignment was performed by Praline search (http://www.ibi.vu.nl/programs/pralinewww/). Phylogenetic analysis was performed with MEGA7.0.21 software. Distinctive domain organization of the protein was determined by SMART search (http://smart.embl-heidelberg.de/). The putative TFs binding sites were identified by YEASTRACT-DISCOVERER Database (http;//yeastract.com). The theoretical pI and molecular weight of the chimeric proteins were determined by Expasy compute pI/Mw tool (http://web.expasy.org/cgi-bin/compute_pi/pi_tool). Protein IDs of protein sequences used in this study are: ArF-BAR: KZM20872.1, ArRab5: KZM19760.1, ArRab7: KZM26450.1, ArActin: KZM21342.1, ArWASP: KZM19192.1, ArCRZ1: KZM25117.1 and ArSNC1: KZM18806.1.

Acknowledgements

We gratefully acknowledge a research grant from Department of Biotechnology, Government of India (File No: BT/PR10605/PBD/16/791/2008 and BT/AGR/CG-Phase II/01/2014) and a core grant from National Institute of Plant Genome Research, New Delhi, India for funding this work. MS, AS, and KS acknowledge Council of Scientific and Industrial Research and University Grants Commission, Government of India for their fellowships.

Author contributions

PKV and VK designed the project; MS, AS, KS and SKS carried out experiments; PKV, KK and KS supervised the work; all authors analysed and discussed the data; MS, AS, KK and VK wrote the manuscript; all the authors reviewed and approved the manuscript; MS and AS contributed equally to this work.

References

1. Takeshita N, Evangelinos M, Zhou L, Serizawa T, Somera-Fajardo RA, Lu L, et al. Pulses of Ca^{2+} coordinate actin assembly and exocytosis for stepwise cell extension. Proc Natl Acad Sci U S A. 2017;114: 5701–5706. doi:10.1073/pnas.1700204114
2. Riquelme M, Aguirre J, Bartnicki-García S, Braus GH, Feldbrügge M, Fleig U, et al. Fungal Morphogenesis, from the Polarized Growth of Hyphae to Complex Reproduction and Infection Structures. Microbiol Mol Biol Rev. 2018. doi:10.1128/mmbr.00068-17
3. Etxebeste O, Espeso EA. Neurons show the path: Tip-to-nucleus communication in

filamentous fungal development and pathogenesisa. FEMS Microbiology Reviews. 2016. doi:10.1093/femsre/fuw021

4. Bielska E, Higuchi Y, Schuster M, Steinberg N, Kilaru S, Talbot NJ, et al. Long-distance endosome trafficking drives fungal effector production during plant infection. Nat Commun. 2014. doi:10.1038/ncomms6097

5. Higuchi Y, Arioka M, Kitamoto K. Endocytic recycling at the tip region in the filamentous fungus *Aspergillus oryzae*. Commun Integr Biol. 2009. doi:10.4161/cib.2.4.8385

6. Higuchi Y, Shoji JY, Arioka M, Kitamoto K. Endocytosis is crucial for cell polarity and apical membrane recycling in the filamentous fungus *Aspergillus oryzae*. Eukaryot Cell. 2009;8: 37–46. doi:10.1128/EC.00207-08

7. Takeshita N. Coordinated process of polarized growth in filamentous fungi. Biosci Biotechnol Biochem. 2016;80: 1693–1699. doi:10.1080/09168451.2016.1179092

8. Takeshita N, Higashitsuji Y, Konzack S, Fischer R. Apical sterol-rich membranes are essential for localizing cell end markers that determine growth directionality in the filamentous fungus *Aspergillus nidulans*. Mol Biol Cell. 2008. doi:10.1091/mbc.E07-06-0523

9. Rao Y, Rückert C, Saenger W, Haucke V. The early steps of endocytosis: From cargo selection to membrane deformation. European Journal of Cell Biology. 2012. doi:10.1016/j.ejcb.2011.02.004

10. Frost A, Perera R, Roux A, Spasov K, Destaing O, Egelman EH, et al. Structural Basis of Membrane Invagination by F-BAR Domains. Cell. 2008. doi:10.1016/j.cell.2007.12.041

11. Habermann B. The BAR-domain family of proteins: a case of bending and binding? The membrane bending and GTPase-binding functions of proteins from the BAR-domain family. EMBO Rep. 2004. doi:10.1038/sj.embor.7400105

12. Krauss M, Haucke V. A novel twist in membrane dephormation. Developmental Cell. 2014. doi:10.1016/j.devcel.2014.09.016

13. Zimmerberg J, Kozlov MM. How proteins produce cellular membrane curvature. Nature Reviews Molecular Cell Biology. 2006. doi:10.1038/nrm1784

14. Saarikangas J, Zhao H, Pykäläinen A, Laurinmäki P, Mattila PK, Kinnunen PKJ, et al. Molecular Mechanisms of Membrane Deformation by I-BAR Domain Proteins. Curr Biol. 2009. doi:10.1016/j.cub.2008.12.029

15. Fuchs U, Hause G, Schuchardt I, Steinberg G. Endocytosis is essential for pathogenic development in the corn smut fungus *Ustilago maydis*. Plant Cell. 2006. doi:10.1105/tpc.105.039388

16. Böhmer C, Ripp C, Bölker M. The germinal centre kinase Don3 triggers the dynamic rearrangement of higher-order septin structures during cytokinesis in *Ustilago maydis*. Mol Microbiol. 2009. doi:10.1111/j.1365-2958.2009.06948.x

17. Lin L, Chen X, Shabbir A, Chen S, Chen X, Wang Z, et al. A putative N-BAR-domain protein is crucially required for the development of hyphae tip appressorium-like structure and its plant infection in *Magnaporthe oryzae*. Phytopathol Res. 2019;1: 1–15. doi:10.1186/s42483-019-0038-2

18. Dagdas YF, Yoshino K, Dagdas G, Ryder LS, Bielska E, Steinberg G, et al. Septin-mediated plant cell invasion by the rice blast fungus, *Magnaporthe oryzae*. Science (80-).

2012;336: 1590–1595. doi:10.1126/science.1222934

19. Soulard A, Lechler T, Spiridonov V, Shevchenko A, Shevchenko A, Li R, et al. Saccharomyces cerevisiae Bzz1p Is Implicated with Type I Myosins in Actin Patch Polarization and Is Able To Recruit Actin-Polymerizing Machinery In Vitro. Mol Cell Biol. 2002. doi:10.1128/mcb.22.22.7889-7906.2002

20. Fricke R, Gohl C, Dharmalingam E, Grevelhörster A, Zahedi B, Harden N, et al. Drosophila Cip4/Toca-1 Integrates Membrane Trafficking and Actin Dynamics through WASP and SCAR/WAVE. Curr Biol. 2009. doi:10.1016/j.cub.2009.07.058

21. Arasada R, Pollard TD. Distinct roles for F-BAR proteins Cdc15p and Bzz1p in actin polymerization at sites of endocytosis in fission yeast. Curr Biol. 2011. doi:10.1016/j.cub.2011.07.046

22. Kumar K, Purayannur S, Kaladhar VC, Parida SK, Verma PK. mQTL-seq and classical mapping implicates the role of an AT-HOOK MOTIF CONTAINING NUCLEAR LOCALIZED (AHL) family gene in *Ascochyta* blight resistance of chickpea. Plant Cell Environ. 2018;41: 2128–2140. doi:10.1111/pce.13177

23. Pandey BK, Singh US, Chaube HS. Mode of Infection of *Ascochyta* Blight of Chickpea Caused by Ascochyta rabiei. J Phytopathol. 1987. doi:10.1111/j.1439-0434.1987.tb04387.x

24. Ilarslan H, Dolar FS. Histological and ultrastructural changes in leaves and stems of resistant and susceptible chickpea cultivars to *Ascochyta rabiei*. J Phytopathol. 2002. doi:10.1046/j.1439-0434.2002.00763.x

25. Verma S, Gazara RK, Nizam S, Parween S, Chattopadhyay D, Verma PK. Draft genome

sequencing and secretome analysis of fungal phytopathogen *Ascochyta rabiei* provides insight into the necrotrophic effector repertoire. Sci Rep. 2016. doi:10.1038/srep24638

26. Singh K, Nizam S, Sinha M, Verma PK. Comparative transcriptome analysis of the necrotrophic fungus *Ascochyta rabiei* during oxidative stress: Insight for fungal survival in the host plant. PLoS One. 2012. doi:10.1371/journal.pone.0033128

27. Nizam S, Singh K, Verma PK. Expression of the fluorescent proteins DsRed and EGFP to visualize early events of colonization of the chickpea blight fungus *Ascochyta rabiei*. Curr Genet. 2010;56: 391–399. doi:10.1007/s00294-010-0305-3

28. Tsujita K, Suetsugu S, Sasaki N, Furutani M, Oikawa T, Takenawa T. Coordination between the actin cytoskeleton and membrane deformation by a novel membrane tubulation domain of PCH proteins is involved in endocytosis. J Cell Biol. 2006. doi:10.1083/jcb.200508091

29. Kumar V, Fricke R, Bhar D, Reddy-Alla S, Krishnan KS, Bogdan S, et al. Syndapin promotes formation of a postsynaptic membrane system in *Drosophila*. Mol Biol Cell. 2009. doi:10.1091/mbc.E08-10-1072

30. Heung LJ, Luberto C, Plowden A, Hannun YA, Del Poeta M. The sphingolipid pathway regulates Pkc1 through the formation of diacylglycerol in *Cryptococcus neoformans*. J Biol Chem. 2004. doi:10.1074/jbc.M312995200

31. Lanver D, Mendoza-Mendoza A, Brachmann A, Kahmann R. Sho1 and Msb2-related proteins regulate appressorium development in the smut fungus *Ustilago maydis*. Plant Cell. 2010. doi:10.1105/tpc.109.073734

32. Valkonen M, Kalkman ER, Saloheimo M, Penttilä M, Read ND, Duncan RR. Spatially

segregated SNARE protein interactions in living fungal cells. J Biol Chem. 2007. doi:10.1074/jbc.M700916200

33. Li YB, Xu R, Liu C, Shen N, Han LB, Tang D. *Magnaporthe oryzae* fimbrin organizes actin networks in the hyphal tip during polar growth and pathogenesis. PLoS Pathog. 2020;16: 1–27. doi:10.1371/journal.ppat.1008437

34. Giraldo MC, Dagdas YF, Gupta YK, Mentlak TA, Yi M, Martinez-Rocha AL, et al. Two distinct secretion systems facilitate tissue invasion by the rice blast fungus *Magnaporthe oryzae*. Nat Commun. 2013. doi:10.1038/ncomms2996

35. Qualmann B, Koch D, Kessels MM. Let's go bananas: Revisiting the endocytic BAR code. EMBO Journal. 2011. doi:10.1038/emboj.2011.266

36. Fischer-Parton S, Parton RM, Hickey PC, Dijksterhuis J, Atkinson HA, Read ND. Confocal microscopy of FM4-64 as a tool for analysing endocytosis and vesicle trafficking in living fungal hyphae. J Microsc. 2000;198: 246–259. doi:10.1046/j.1365-2818.2000.00708.x

37. Zeigerer A, Gilleron J, Bogorad RL, Marsico G, Nonaka H, Seifert S, et al. Rab5 is necessary for the biogenesis of the endolysosomal system in vivo. Nature. 2012. doi:10.1038/nature11133

38. Steinberg G. Endocytosis and early endosome motility in filamentous fungi. Curr Opin Microbiol. 2014;20: 10–18. doi:10.1016/j.mib.2014.04.001

39. Almeida-Souza L, Frank RAW, García-Nafría J, Colussi A, Gunawardana N, Johnson CM, et al. A Flat BAR Protein Promotes Actin Polymerization at the Base of Clathrin-Coated Pits. Cell. 2018. doi:10.1016/j.cell.2018.05.020

40. Berepiki A, Lichius A, Read ND. Actin organization and dynamics in filamentous fungi. Nat Rev Microbiol. 2011;9: 876–887. doi:10.1038/nrmicro2666

41. Riedl J, Crevenna AH, Kessenbrock K, Yu JH, Neukirchen D, Bista M, et al. Lifeact: A versatile marker to visualize F-actin. Nat Methods. 2008. doi:10.1038/nmeth.1220

42. Monteiro PT, Oliveira J, Pais P, Antunes M, Palma M, Cavalheiro M, et al. YEASTRACT+: A portal for cross-species comparative genomics of transcription regulation in yeasts. Nucleic Acids Res. 2020. doi:10.1093/nar/gkz859

43. Park HS, Lee SC, Cardenas ME, Heitman J. Calcium-Calmodulin-Calcineurin Signaling: A Globally Conserved Virulence Cascade in Eukaryotic Microbial Pathogens. Cell Host Microbe. 2019;26: 453–462. doi:10.1016/j.chom.2019.08.004

44. Hernández-Ortiz P, Espeso EA. Spatiotemporal dynamics of the calcineurin target CrzA. Cell Signal. 2017. doi:10.1016/j.cellsig.2016.11.005

45. Yoshimoto H, Saltsman K, Gasch AP, Li HX, Ogawa N, Botstein D, et al. . Genome-wide analysis of gene expression regulated by the calcineurin/Crz1p signalingpathway in Saccharomyces cerevisiae. J Biol Chem. 2002.doi:https://doi.org/10.1074/jbc.M202718200

46. Li X, Gao C, Li L, Liu M, Yin Z, Zhang H, et al. MoEnd3 regulates appressorium formation and virulence through mediating endocytosis in rice blast fungus *Magnaporthe oryzae*. PLoS Pathog. 2017. doi:10.1371/journal.ppat.1006449

47. Leibfried A, Fricke R, Morgan MJ, Bogdan S, Bellaiche Y. Drosophila Cip4 and WASp Define a Branch of the Cdc42-Par6-aPKC Pathway Regulating E-Cadherin Endocytosis. Curr Biol. 2008. doi:10.1016/j.cub.2008.09.063

48. Grove SN, Bracker CE. Protoplasmic organization of hyphal tips among fungi: vesicles and Spitzenkörper. J Bacteriol. 1970. doi:10.1128/jb.104.2.989-1009.1970

49. Peñalva MÁ. Endocytosis in filamentous fungi: Cinderella gets her reward. Curr Opin Microbiol. 2010;13: 684–692. doi:10.1016/j.mib.2010.09.005

50. Lo Presti L, Lanver D, Schweizer G, Tanaka S, Liang L, Tollot M, et al. Fungal effectors and plant susceptibility. Annu Rev Plant Biol. 2015. doi:10.1146/annurev-arplant-043014-114623

51. Singh Y, Nair AM, Verma PK. Surviving the odds: from perception to survival of fungal phytopathogens under host-generated oxidative burst. Plant Communications. 2021. doi:10.1016/j.xplc.2021.100142

52. de Castro PA, Colabardini AC, Manfiolli AO, Chiaratto J, Silva LP, Mattos EC, et al. *Aspergillus fumigatus* calcium-responsive transcription factors regulate cell wall architecture promoting stress tolerance, virulence and caspofungin resistance. PLoS Genetics. 2019. doi:10.1371/journal.pgen.1008551

53. Harris SD. Septum formation in *Aspergillus nidulans*. Curr Opin Microbiol. 2001;4: 736–739. doi:10.1016/S1369-5274(01)00276-4

54. Frost A, Unger VM, De Camilli P. The BAR Domain Superfamily: Membrane-Molding Macromolecules. Cell. 2009. doi:10.1016/j.cell.2009.04.010

55. Delgado-Álvarez DL, Bartnicki-García S, Seiler S, Mouriño-Pérez RR. Septum development in *Neurospora crassa*: The septal actomyosin tangle. PLoS One. 2014;9: 29–30. doi:10.1371/journal.pone.0096744

56. Guertin DA, Trautmann S, McCollum D. (CUL-ID:2436029) Cytokinesis in Eukaryotes.

Microbiol Mol Biol Rev. 2002. doi:10.1128/MMBR.66.2.155-178.2002

57. Wu JQ, Kuhn JR, Kovar DR, Pollard TD. Spatial and temporal pathway for assembly and constriction of the contractile ring in fission yeast cytokinesis. Dev Cell. 2003. doi:10.1016/S1534-5807(03)00324-1

58. Martín-García R, Arribas V, Coll PM, Pinar M, Viana RA, Rincón SA, et al. Paxillin-Mediated Recruitment of Calcineurin to the Contractile Ring Is Required for the Correct Progression of Cytokinesis in Fission Yeast. Cell Rep. 2018. doi:10.1016/j.celrep.2018.09.062

59. Lamb CJ, Lawton MA, Dron M, Dixon RA. Signals and transduction mechanisms for activation of plant defenses against microbial attack. Cell. 1989. doi:10.1016/0092-8674(89)90894-5

60. Livak KJ, Schmittgen TD. Analysis of relative gene expression data using real-time quantitative PCR and the $2^{-\Delta\Delta CT}$ method. Methods. 2001. doi:10.1006/meth.2001.1262

61. Talbot NJ, Ebbole DJ, Hamer JE. Identification and characterization of MPG1, a gene involved in pathogenicity from the rice blast fungus *Magnaporthe grisea*. Plant Cell. 1993. doi:10.1105/tpc.5.11.1575
