# Supplementary material for: Modulation of fungal virulence through CRZ1 regulated F-BAR-dependent actin remodeling and endocytosis in chickpea infecting phytopathogen Ascochyta rabiei
Source: PLoS Genet. 2021 May 17;17(5):e1009137. doi: 10.1371/journal.pgen.1009137 (PMC8158962; doi:10.1371/journal.pgen.1009137)
Supplement: Attachment — Submitted filename: Reviewer Recommendation and Comments for Manuscript Number PGENETICS.pdf [file pgen.1009137.s022.pdf]

Reviewer Recommendation and Comments for Manuscript Number PGENETICS-D-20-01505

Modulation of fungal virulence through CRZ1 regulated F-BAR-dependent actin remodelling and endocytosis in chickpea infecting phytopathogen Ascochyta rabiei

March 2021

The study by Sihna *et al.,* reports the identification and characterization of a Bin1/Amphiphysin/Rvs167 (BAR) domain containing protein called ArF-BAR in the ascomycete fungus *Ascochyta rabiei*. In this study it is shown that ArF-BAR is induced by oxidative stress and regulated by the Calcineurin-responsive zinc finger 1 transcription factor CRZ1. ArF-BAR controls endocytosis, actin dynamics and septum formation. But ArF-BAR is also is involved in secretion and pathogenicity. Authors now showed that that secretion of effector Ar93 is ArF-BAR dependent.

In my view this study is well executed. Experiments and evidence shown meet the conclusions and findings proposed.

I have just few minor comments.

L28- “ArF results in delayed formation of apical septum from the growing hyphal tip, crucial for host penetration and impaired secretion of candidate effector having signal peptide”. Sentence confusing.

Fig 5B- increase size of the panels. They are too small.

Fig 5A- Quantification cell size in panel 5A inside the plant. Could authors quantify cell1 vs cell 2? How many hyphae move from cell 1 vs cell 2 iin WT vs. mutant?

L303 EEs state first that are Early Endosomes.

L292 This should be in lower case after coma.

L326-327 “ interactions of ArF-BAR with ArActin.g Y2H system, it was shown that ArF-BAR does not interact directly with actin (Fig 7A),” This sentences is confusing

L357-361 How was the length of the ArF-BAR promoter chosen on the first place? Was it arbitrary to take 1kb? It will be useful to show all the putative binding sites of each transcription factor. This is a beautiful analysis that needs to be better explained and shown.

L382- to silence- change to inhibit
