# Supplementary material for: Modulation of fungal virulence through CRZ1 regulated F-BAR-dependent actin remodeling and endocytosis in chickpea infecting phytopathogen Ascochyta rabiei
Source: PLoS Genet. 2021 May 17;17(5):e1009137. doi: 10.1371/journal.pgen.1009137 (PMC8158962; doi:10.1371/journal.pgen.1009137)
Supplement: Attachment — Submitted filename: PGENETICS-D-20-01505.docx [file pgen.1009137.s019.docx]

**Reviewer Recommendation and Comments for Manuscript Number PGENETICS-D-20-01505**

Modulation of fungal virulence through CRZ1 regulated F-BAR-dependent actin remodeling and endocytosis in chickpea infecting phytopathogen Ascochyta rabiei

The manuscript by Sihna *et al.,* reports the identification and characterization of a Bin1/Amphiphysin/Rvs167 (BAR) domain containing protein called ArF-BAR in the ascomycete fungus *Ascochyta rabiei*. Authors report that ArF-BAR is involved in endocytosis and actin dynamics, and that it is required for fungal pathogenicity. The study also shows that the Calcineurin-responsive zinc finger 1 transcription factor called CRZ1 regulates ArF-BAR transcription upon oxidative stress by binding to ArF-BAR promoter region.

Generally, I found this manuscript enjoyable to read. The study is well executed, and well written. But I have a few comments. I feel that the text has to explain experiments and conclusions in more detail. It lacks specificity when stating conclusions and when experiments are described. Improving this will help strengthening the manuscript.

- In paragraph from L123- 132, and Fig.1B authors show that ArF-BAR expression is triggered by oxidative stress. In Fig.1C authors show that the relative gene expression of ArF-BAR is increased at 24hpi which is the time at which spores germinate on the host surface. Does this mean that at 24h the fungus has not penetrated the host tissue? According to Fig.S7 the formation of primary invasive hyphae happens at 48h. How is the developmental stage of the interaction between the fungus and the host over a time course? The way I understood this was that oxidative stress happened after penetration of the host tissue. Please could authors maybe provide detailed micrographs of a time course of the invasion of the host? Also, why ArF-BAR will be induced just at the host surface? Also, null mutants of DArF-BAR were not able to penetrate more than 9.83 um. This could suggest that the mutant has a defect in coping with oxidative stress inside the plant. Can authors stain with Diaminobenzidine (DAB) to see if ROS production is increased in DArF-BAR during epidermis infection? It might well be that as DArF-BAR is impaired in growth and also in coping with plant defence responses, have authors tested whether DArF-BAR has impaired secretion?
- The tubulation micrograph of Fig. S3B is much better than the one used on the main Fig 1D-G. Please can you provide better images for Fig 1? It will be also good to include zoomed areas such as in Fig. 3SB to show the difference between the mutants and the wild type, as current images are not very clear. Also, please can this be quantified to denote the relevance? Tubular vs. Non-tubular
- Authors have not found differences in liposome tubulation when W428 and L430 residues were mutated, however what happens if the entire PKC domain is deleted? Maybe those exact residues are not purely relevant, but the study of the entire domain could provide more substantial information. What will happen to the pathogenicity, the localization?
- Further explanation on how the list of putative transcription factors regulating ArF-BAR should be included. It is very good that a list of candidate transcription factors was identified. However it is necessary to explain better what was used as query to identify this list? Was CRZ1 just selected based on the relationship with stress responses or was there any other evidence? Were other TFs tested? More elaboration on this part is necessary as it is such as important finding that it is hard to understand from the manuscript how CRZ1 was identified. Elaborate materials and methods section too.
- Show localization of ArF-BAR, Rab5 and Rab7 in Dcrz1 mutant. If CRZ1 mutant operates upstream of ArF-BAR, they should be miss-localized.
- In the model, authors suggest that upon ROS perception, intracellular Ca+2 increases to activate CRZ1. Authors show that CRZ1 localises to nuclei during infection (Fig. S12B). I feel that Fig. S12B in which the nuclear localization of CRZ1 is shown during invasive growth should be in the main figure. It is an important and fits with the model. I think it is necessary to show better imaging and include a DIC panel. Also over a time course, is CRZ1 translocated once the fungus has penetrated the host tissue penetrates?

Minor points:

- L115- reference 27 doesn’t correspond to the reference of the genome
- L171- instead of infection bioassay, it will be better to specify that it is a spray inoculation
- L225- ArF-BAR::EYFP was transiently expressed? Could authors state how, which promoter? According to Fig. S5 is native promoter. This needs clarification.
- Fig1B- was this done in mycelium treatment with menadione or spores? State it in the legend of the figure. Also, please could authors state what was the standard used for the qPCR?
- Fig S10B- Line scan legend very small. Please increase font.
- Y2H experiments need to be explained better, at least in the legend or in materials and methods. Please state what are the controls too.
- State what is A.U. in the figure legends.
- L129- “Consistent with previous observations”
- Legend pannel D fig 1 is missing.
- State what is A.U. in legends of figures.
- Was fluorescence intensity calculated per area? E.g. Figure 4.
- Explain legend and controls Fig. 5
- Fig. S4. Southern blot, explain better the output of the sizes.
