# Supplementary material for: Modulation of fungal virulence through CRZ1 regulated F-BAR-dependent actin remodeling and endocytosis in chickpea infecting phytopathogen Ascochyta rabiei
Source: PLoS Genet. 2021 May 17;17(5):e1009137. doi: 10.1371/journal.pgen.1009137 (PMC8158962; doi:10.1371/journal.pgen.1009137)
Supplement: Attachment — Submitted filename: Response to reviewers.doc [file pgen.1009137.s020.doc]

**Response to reviewers**

Authors are thankful to the editor and anonymous reviewers for giving us positive and encouraging comments, and the opportunity to revise our research article manuscript (PGENETICS-D-20-01505). We hope that this improved manuscript will be accepted now for publication in PloS Genetics journal.

**Response to Reviewer #1**

**General comments by Reviewer #1:**

The paper setup is ok and the microscopy, and figures are pretty decent.

**Response:** Authors are thankful to the reviewer for the suggestions to improve the quality of our manuscript and words of encouragement. In the revised manuscript, we have made several changes in text and figures as per the overall suggestions of all the three reviewers. Point-by-point authors’ responses on comments are given below;

**Comment 1:** The species name should include a mention of *Didymella* *rabiei*. Also, the pathovar should be indicated.

**Response:** *Didymella* *rabiei* is the teleomorph of *Ascochyta* *rabiei*, which is the anamorph. We had included ‘*Didymella* *rabiei*’ name in the original manuscript under introduction section since both names are used in literature. We used *A*. *rabiei* mating type 2 (MAT1–2) isolate (ArD2; ITCC No. 4638) in the current study and based on Ascochyta blight disease development on chickpea differential lines it is ‘pathotype II’. This is now mentioned in the revised manuscript’s sub-section ‘Fungal strains and growth conditions’. The same isolate was previously used in published articles for genome sequence (Verma et al., 2016; Scientific Reports 6, 24638) and resistance QTLs analysis in chickpea (Kumar et al, 2018; Plant Cell & Environ. 41:2128–2140) by our research group.

**Comment 2:** L 23-25 The authors state “The loss-of- function of ArF-BAR results in delayed formation of first septa from the hyphal tip, crucial for host penetration and proliferation” Yet, a direct microscopic observation of appressorium formation/penetration of the plant cuticula is not presented. Instead, the paper turns to genetic regulation of ArF-BAR and identifies ArCrz1. The importance of this regulation is not clear, however, as a role for ArCrz1 in virulence has not been determined.

**Response:** We have shown in Fig 5B that Δ*arf*-*bar* mutant has delayed septa formation. However, we have quantified this data to make it more convincing, which is presented in the revised manuscript Fig 5C graph. It shows that Δ*arf*-*bar* mutant has average size (length) ~3 times of 1^st^ cell and ~2 times of 2^nd^ cell from the hyphal tip as compared to wild-type *A*. *rabiei* after *in* *vitro* spore germination. We had analyzed the fungal growth of WT and Δ*arf*-*bar* mutant on plant host in several independent experiments. Based on those observations, we have shown in Fig 5A and Fig S7 that the penetration of chickpea cuticula and internal cells is reduced to half in Δ*arf*-*bar* mutant as compared to wild-type *A*. *rabiei*. We had quantified the depth of chickpea tissue penetration by WT and Δ*arf*-*bar* mutant strains till 48 hpi so that most of Δ*arf*-*bar* hyphae can have enough time to penetrate host tissue. Still, majority of the Δ*arf*-*bar* mutant hyphae failed to penetrate beyond 10–15 µm.

In the manuscript, we aimed towards showing role of ArF-BAR in fungal disease development on chickpea and other molecular aspects of ArF-BAR based on its domains. As per our literature survey, the transcriptional regulation of *ArF*-*BAR* gene has not been shown in any of fungal phytopathogen so we focused on its transcriptional regulation by ArCRZ1. In the manuscript, we showed the role of ArCRZ1 in *Ascochyta* *rabiei* virulence in Fig 9. Our result suggests that Δ*arf*-*bar* mutant is less virulent as compared to WT. It can be inferred from reduced number of *Ascochyta* blight disease lesions per plant and the lesion diameter.

**Comment 3:** Fig 1A: beyond a comparison in suppl figures S1 and S2 it is of more interest to see if the domain structure is also conserved. The ScBzz1/Las7 actually interacts with type I myosins…

**Response:** As the current manuscript deals with initial characterization of ArF-BAR, therefore, we focused ourselves to identify the role of *Ascochyta* *rabiei* F-BAR on pathogen growth and virulence on host tissue. The molecular functions of tubulation, co-localization with endocytosis markers, actin-polymerization, SH3 interaction and dimerization, presented in this manuscript are based on the domains present in the ArF-BAR. Reviewers may agree with us on the point that the identification of novel interacting partners for ArF-BAR and ascertain novel function to it can itself become an independent study on ArF-BAR. We hope to carry out such interesting study in near future independent of the current manuscript.

**Comment 4:** Fig 1B, the authors show that expression of *ArF*-*BAR* is induced upon germination. Yet they state that this equal, “transcript induction during pathogenesis”. I tend to disagree.

**Response:** The Fig 1B of our manuscript represents the qRT-PCR data of relative *ArF*-*BAR* transcript level expression in mycelia after menadione treatment as compared to the non-treated control mycelia. Here, the transcripts get induced due to oxidative stress (menadione treatment). However, we have shown the *ArF*-*BAR* transcript level expression during the host hyphae/mycelia growth on host tissue in Fig 1C. The time points selected in Fig 1C are up to 72 hrs post conidial inoculation on chickpea host. As during chickpea-*Ascochyta* interaction, basal defence response of chickpea generates oxidative stress, so we have presented this data in Fig 1C and *in* *vitro* oxidative stress treatment data in Fig 1B. Hope, now the reviewer will agree that *ArF*-*BAR* transcript show higher expression during oxidative stress (*in* *vitro* menadione treatment) and during proliferation on host tissue.

**Comment 5:** The mutant shows slow growth (Fig. 2). This could reduce virulence by itself as an indirect effect. This should be discussed more clearly.

**Response:** The Δ*arf*-*bar* mutant growth on PDA plate after 7 days of incubation show reduced growth (Fig 2A and Fig 3; radial diameter on PDA plates). However, our repeated analysis of *in* *vitro* conidial hyphae growth does not show such significant difference during early growth up to 48 hrs post conidial germination although septa formation is delayed. However, other stresses (like osmotic stress) during *in* *vitro* growth on PDA plates (up to 7 days) may hamper mycelial growth. The Δ*arf*-*bar* mutant strain was found to be defective in effector secretion as compared to WT in a new experiment performed during the revision (Fig 5D). Now, it is clear that inability of mutant strain to cope with the host generated stresses may reduce Δ*arf*-*bar* mutant growth and penetration of the host tissue. Thus, *in* *vitro* growth reduction in Δ*arf*-*bar* mutant is not comparable to the growth on host plant tissue. Although, indirect effect of delayed septation on virulence cannot be neglected that we have included this in the ‘discussion’ section of the revised manuscript.

**Comment 6:** Figure 2C shows that there are a few lesions formed in the plant virulence assay. Thus, the authors should not state that ArF is essential for virulence, but required for full virulence as stated in L179+.

**Response:** We agree with reviewer’s suggested sentence and have modified it in the revised manuscript.

**Comment 7:** L172, Typical AB disease symptoms were observed “what is meant with AB?”

**Response:** *A. rabiei* infects all the above ground parts of the chickpea plant. Initially, disease appears as water-soaked lesions on the leaves, which at later stage turns brown. Disease symptoms become visible as ‘small circular necrotic lesions’ on leaves and ‘elongated brown lesions’ on stems. These are called typical *Ascochyta* blight symptoms. However, in the revised manuscript we have elaborated this point in more detail for the general readers.

**Comment 8:** L191, Again, the authors state that *ArF*-*BAR* is indispensable for fungal pathogenicity. This is double ungood. First, it is dispensable, but required for full virulence; second, the authors make an extrapolation on fungal virulence in general that is not warranted. In light of the results also the PKC domain is required for full virulence, a point the authors just dropped.

**Response:** We have toned down on some of these statements that talk about pathogenicity. In L191, of the original manuscript, we wanted to convey that *ArF*-*BAR* gene is required for full virulence as its mutant shows reduced *Ascochyta* blight (AB) symptoms (Fig 2B-D) on chickpea. Besides this, the complementation of Δ*arf*-*bar* mutant with *ArF*-*BAR*^mut1^ construct also has similar level of reduced symptoms. Hence, in L191, we also wanted to convey that ArF-BAR domain residues are important for virulence on chickpea. We have made appropriate changes in the revised manuscript as per your suggestions. The role of C1 domain of ArF-BAR (wrongly marked as PKC domain in the previous version of manuscript as it showed high similarity with C1 domain of PKC1 protein) has also been discussed in *Ascochyta* *rabiei* virulence.

**Comment 9:** The genetic analysis is ok – yet, there may be issues with protein stability of the mutants that at least need to be discussed.

**Response:** We had not tagged the complementation constructs with any of the epitope so checking protein stability is difficult. We avoided epitope tag so that in complementation constructs the protein expresses similar to wild-type. However, fluorescence of chimeric mutants with eYFP (Fig 4A) rules out the question of mutant proteins stability. We hope reviewer must be now satisfied that mutant proteins are stable. We have discussed the same in revised manuscript.

**Comment 10:** L193-205, the observed effect of reduced penetration could also be a consequence of the slower growth, this needs to be discussed.

**Response:** Our views on this point may have been cleared by our response to your comment#5. Still, we have appropriately taken this point in the discussion section of revised manuscript considering delayed septa formation in Δ*arf*-*bar* mutant.

**Comment 11:** L206, ArF-BAR contains a PKC domain. To me this hint to a cell wall remodelling role. Oxidative stress is not the first stress that comes to mind then but rather osmotic stresses.

**Response:** We regret for mentioning C1 domain (~50 amino acids) as PKC domain in the original manuscript as explained in the author response of your comment#8. Now, this anomaly has been corrected in the revised manuscript. We did not mention osmotic stress during conidial germination on host tissue as during conidial inoculation enough humidity is maintained in growth chambers. The chickpea aerial tissue also traps lot of moisture due to high trichome density. In field conditions too, the conidia start germinating on chickpea host only with the help of moisture from dew/ intermittent rain in the winter growing season. We hope now that the reviewer will agree with our reason of not considering osmotic stress. Although, role of ArF-BAR in cell wall remodeling can’t be ruled out and the same has been integrated in ‘discussion’ section of the revised manuscript.

**Comment 12:** L206-221: if the authors want to stress this, they should move the data from supplemental to results. I suggest to be more critical with these results. Stats need to include the general growth defect of the mutant and recalculate the relative growth inhibition on top of that.

**Response:** We agree with the reviewer and have appropriately placed the figure in main results. The point of relative growth inhibition is also well taken. The growth inhibition by menadione and H_2_O_2_ (in %; relative to PDA only) has been shown in the revised manuscript Fig S8.

**Comment 13:** L225: how was transient expression of YFP achieved. This is not clear from the methods section.

**Response:** In our study, the expression of all the T-DNA constructs in *Ascochyta* *rabiei* was performed by stable transformation by *Agrobacterium* mediated T-DNA transfer and antibiotic selection. The ‘transient expression’ was inadvertently written in this sentence and the word has been appropriately corrected in the revised manuscript. A published reference of *A*. *rabiei* transformation (Nizam et al. 2010; Curr Genet. 56:391–399.) has also been provided with the revised text.

**Comment 14:** L238-245: a statistic on the distribution of septa in the hyphae seems warranted to see if the spacing is random, irregular or if the compartments are simply larger than in WT.

**Response:** A full statistic data on the length of 1^st^ and 2^nd^ cell (in µm) from hyphae tip is now presented in the revised manuscript Fig 5B and 5C.

**Comment 15:** The YFP data would be improved by a co-staining of the actin cytoskeleton. This would show that ArF-BAR colocalizes with actin patches.

**Response:** To visualize actin in WT and Δ*arf*-*bar* mutant, we had tagged the mCherry fluorescent protein with Lifeact, a 17-amino-acid peptide, which decorates filamentous actin (F-actin) structures in eukaryotic cells and tissues (Fig 7D). To better visualize the colocalization of actin patches with EYFP in ArF-BAR::EYFP transformed *A*. *rabiei* cells, the hyphae were co-stained with Alexa Fluor^TM^ 635 Phalloidin (Catalog number: A34054, ThermoFisher Scientific). However, after repeated experiments we could not observe the dye labelled signals of actin cytoskeleton after fixation as the fluorescence was not properly obtained. This may be due to the technical glitches as *A*. *rabiei* fixation protocol is not well standardized.

**Comment 16:** The colocalization data of ArF-BAR with Rab5 and not with Rab7 is interesting. The suppl material on Rab7 needs to be moved to the results next to Rab5.

**Response:** As per suggestion, we have appropriately moved the Rab5 and Rab7 data together in Fig 6B and 6C of the revised manuscript.

**Comment 17:** Overall: The paper comes with a ton of results. This is very nice. Yet, the authors also produce a ton of figures and suppl material that overwhelms and distracts from the novel findings. The authors should better indicate what is known from ArF homologs and build on that. The knowns may go to supplemental and the novel stuff should be highlighted in the figures.

**Response:** We agree on the overall views expressed by the reviewer and accordingly placed the data and improved manuscript text too. We hope that the new experiments carried out during revision and implementations of suggestions from all three reviewers have significantly improved the quality of our revised manuscript.

**Response to Reviewer #2**

**General comments by Reviewer #2:**

In my opinion, the work done is of interest for the readers of PloS Genetics. However, some points need to be addressed before I recommend its publication.

**Response:** We thank reviewer for giving his positive comments about our manuscript and study. We have taken a note of all your queries and suggestions along with other reviewers’ views to improve our revised manuscript.

**Comment 1:** Although the manuscript is well written, it needs improvement and rewriting of several sentences.

**Response:** We have made several changes as per your suggestions mentioned in the original manuscript pdf file. We extend our thanks for such detailed analysis of our manuscript and hope you will find these points appropriately corrected in the revised manuscript.

**Comment 2:** For a better characterization of ArF-BAR, the double point mutant should be generated and its phenotype compared with those of Mut1 and Mut2.

**Response:** In our study, we generated knock-out mutant (Δ*arf*-*bar*) to characterize the role of *ArF*-*BAR* in *Ascochyta* *rabiei* pathogen. The Δ*arf*-*bar* mutant showed drastically reduced virulence as compared to WT (Fig 2B-D). As the ArF-BAR protein has two important domains of F-BAR and C1, we generated mut1 and mut2 complementation constructs to check the roles of these two domains, individually. However, the lack of intact F-BAR (residues of F-BAR mutated in complementation construct mut1) and Δ*arf*-*bar* mutant showed comparable reduced virulence (Fig 2B-D) as compared to the mut2 complementation construct. Therefore, we believe that generation of double mutations complementation construct (mut1 and mut2 together) would not reduce *A*. *rabiei* virulence beyond Δ*arf*-*bar* mutant. We hope the reviewer will now agree with our clarification.

**Comment 3:** The apical localization of ArF-BAR should be tracked in more detail. Additional markers of exocytosis and the Spitzenkörper should be used with that aim.

**Response:** We have examined the ArF-BAR localization in more detail during the period of this revision by tagging ArF-BAR with YFP and transforming it with ArSNC1::mCherry marker (Fig 4C). The SNC1 has been used extensively in living fungal cells (Valkonen et al. 2007; J Biol Chem. 282:22775–22785) and *M*. *oryzae* (Giraldo et al. 2013; Nat Commun. 4:1996) as a marker for exocytosis marker. The result is now presented in Fig 4C of the revised manuscript. More number of markers for endocytosis, exocytosis and Spitzenkörper are yet to be standardized for *A*. *rabiei* since the N-terminal or C-terminal tagging and overexpression of chimeric proteins also changes their localization pattern.

**Comment 4:** How do the authors explain the difference in ArF-BAR localization in Figure 3A compared to that shown in Figure 4B? Is it only a matter of the Z-position? In addition, the authors should indicate the hyphal region plotted in the graph of panel 4B.

**Response:** The difference is original manuscript’s Fig 3A and 4B (Revised manuscript’s Fig4A and 6B) is only due to the Z-position and different regions of hyphae targeted during image acquisition. The difference between Fig 4A and Fig 6B is about magnification and region of hyphae/mycelium. The Fig 4A, at higher magnification, of the hyphae is to show vesicle formation towards the growing tip, while in Fig 6B, the same phenomenon is shown at the other phase/growth region of mycelia at lower magnification. Otherwise, the localization of mCherry and YFP tagged ArF-BAR remains similar. The Fig 6C can be also considered for better comparison of all the three figures. The hyphal region plotted in fluorescence intensity graphs has been now been marked with a magenta line in the merged images of revised Fig 6B and 6C.

**Comment 5:** I recommend to tone down any mention to a hypothetic direct role of ArF-BAR in endocytosis. I would say that what the authors have shown is a defect in endocytosis in the null arf-bar mutant, and that not necessarily means that ArF-BAR is directly involved in early endosome biogenesis and endocytosis.

**Response:** We have taken note of your point and appropriately corrected the sentences in the revised manuscript.

**Comment 6:** I would show the phenotype of the null crz mutant under stress conditions as a panel of Figure 7 and not as supplementary material.

**Response:** The data shown for Δ*arcrz1* phenotype under stress conditions in Fig S14 (original manuscript’s Fig S13) was just to ascertain that *ArCRZ1* behaves similar under to the other published stresses in fungal system. Hence, we have kept the data in supplementary data so that new data about *A. rabiei* genes remain in main figures. We hope you will agree with our explanation.

**Comment 6:** Finally, I recommend a more detailed description of some of the procedures used. I attach a pdf file of the manuscript with all my comments and corrections. Hope it will help.

**Response:** The authors are very much obliged by such detailed evaluation and point to point suggestions in the pdf file. We have improved upon all your suggestions in the revised manuscript.

**Authors Response to Reviewer # 3**

**General comments by Reviewer # 3:**

Generally, I found this manuscript enjoyable to read. The study is well executed, and well written. But I have a few comments. I feel that the text has to explain experiments and conclusions in more detail. It lacks specificity when stating conclusions and when experiments are described. Improving this will help strengthening the manuscript.

**Response:** Authors thank the anonymous reviewer for praising our manuscript. Your words of praise have made us set up some new experiments for the revision. We have made changes in the revised manuscript as per your suggestions. Hope you will applaud these changes in the revised manuscript. Below we have written our responses to your concerns and explained about the changes that we have made in the revised manuscript.

**Major Comment 1a:** In paragraph from L123- 132, and Fig.1B authors show that ArF-BAR expression is triggered by oxidative stress. In Fig.1C authors show that the relative gene expression of ArF-BAR is increased at 24 hpi which is the time at which spores germinate on the host surface. Does this mean that at 24h the fungus has not penetrated the host tissue? According to Fig.S7 the formation of primary invasive hyphae happens at 48h. How is the developmental stage of the interaction between the fungus and the host over a time course? The way I understood this was that oxidative stress happened after penetration of the host tissue. Please could authors maybe provide detailed micrographs of a time course of the invasion of the host? Also, why ArF-BAR will be induced just at the host surface?

**Response 1a:** Our group has been studying *Ascochyta* *rabiei* development on various accessions of chickpea (susceptible, tolerant and resistant). A general finding of our group and other researchers is that on chickpea foliar tissue, the conidia start germinating on most chickpea accessions at ~ 6 hpi under suitable conditions of moisture and temperature. Although a variation in germination between biological replicates and isolates is observed on chickpea host accessions, which is acceptable. Generally, the *A*. *rabiei* spores germinate at different rate from 6 to 18 hpi. However, most hyphae that germinated early starts penetrating host tissue ~24 hpi and about the same time an increased H_2_O_2_ accumulation has been observed at site of fungal penetration. Our qRT-PCR results on *ArF*-*BAR* transcript expression suggested that its transcripts get induced ~5 fold at 1 h post menadione treatment of fungal hyphae (Fig. 1B) while on chickpea host it gets induced ~6 fold at 24 hpi (Fig. 1C). The H_2_O_2_ accumulation in chickpea host at ~24 hpi near the sites of penetration and highest accumulation of *ArF*-*BAR* happens around the same time. This observation suggests that on chickpea tissue, the host basal defense response generated oxidative stress or H_2_O_2_ initiates the fungal signaling for *ArF*-*BAR* transcriptional-level induction. The original EST clone (*Ar72*) of *ArF*-*BAR* was also isolated as an menadione induced transcript from *A*. *rabiei* mycelium through suppression subtractive hybridization (SSH) EST library (Singh *et* *al*., 2012; PLoS ONE 7(3): e33128).

In Fig S7, the WT and Δ*arf*-*bar* are compared for growth and penetration of host tissue at 48 hpi by observing the penetrations at different depths (Z: 0 to -23 µm). The previous observations of a slower growth of Δ*arf*-*bar* strain as compared to *A*. *rabiei* WT strain on PDA plates persuaded us to consider 48 hpi as the time point so that most of Δ*arf*-*bar* hyphae can have enough time to penetrate host tissue. As per our observation for WT strain, many primary invasive hyphae have penetrated host tissue ~24 hpi and started proliferating between epidermal cells while for Δ*arf*-*bar* the hyphae have not penetrated below 10-15 µm. This can be due to mutant (Δ*arf*-*bar*) incapability to grow under the adverse conditions of host-generated defenses and inability to suppress host defense.

To examine a possible reason behind it, we performed secretion of a candidate effector having classical signal sequences in WT and Δ*arf*-*bar* mutant. In a new experiment performed during the manuscript revision, we have shown that Δ*arf*-*bar* mutant has defect in fungal effector secretion (Fig 5D). The details of this experiment are mentioned in our response to your comment #1C and main text of the revised manuscript. The results of this new experiment clearly suggest that Δ*arf*-*bar* mutant has impaired secretion of effectors or other secreted biomolecules. This impaired secretion may result in its inability to properly strengthen its cell wall or inability to overcome the stresses caused by host basal defense leading towards a slower growth on chickpea tissue. We have incorporated the new experiment results and made several changes in the revised manuscript as per suggestions in your comment to make it understandable for readers.

**Major Comment 1b:** Also, null mutants of Δ*arf*-*bar* were not able to penetrate more than 9.83 µm. This could suggest that the mutant has a defect in coping with oxidative stress inside the plant.

**Response 1b:** We agree with reviewer on this point as the Δ*arf*-*bar* mutant strain showed less growth under H_2_O_2_ and menadione treatments as compared to WT and complemented strains on the PDA plates Fig 3 (Fig S8 of original manuscript). Beside this defect, the Δ*arf*-*bar* mutant strain is defective in effector secretion as revealed by the newly incorporated results obtained during the revision of manuscript (explained in response to your comment #1C). This inability to secrete effectors, resulting into less suppression of host defense response, may be responsible for delayed growth and limited penetration of host tissue. The cell wall integrity of fungal penetrating hyphae can also be questioned as cell wall repair is important for hyphal protection. These reasons have been discussed in text of the revised manuscript.

**Comment 1c:** Can authors stain with Diaminobenzidine (DAB) to see if ROS production is increased in Δ*arf*-*bar* during epidermis infection? It might well be that as Δ*arf*-*bar* is impaired in growth and also in coping with plant defence responses, have authors tested whether Δ*arf*-*bar* has impaired secretion?

**Response 1c:** This is a nice suggestion and we tried the same in various chickpea-*A.* *rabiei* interaction studies with 3,3′-Diaminobenzidine (DAB) but could only observe H_2_O_2_ accumulation in the chickpea cells, around the site of fungal invasion, and not inside the fungal cells (Singh *et* *al*., 2012; PLoS ONE 7(3): e33128). Technically it is very difficult to monitor intracellular H_2_O_2_-level fluctuations inside *Ascochyta* *rabiei* cells during chickpea epidermis growth with staining methods like DAB and 2′,7′-dichlorodihydrofluorescein diacetate (H_2_DCFDA). The main difficulty with H_2_DCFDA is that fluorogenic dye signals cannot be differentiated between chickpea host and fungal cells.

We have considered reviewer’s suggestion and introduced a new experiment to check whether Δ*arf*-*bar* mutant has impaired secretion of fungal effectors. Since, effectors help in successful colonization of plant tissue through various modifications in host cells. In this exocytosis experiment, a candidate *A*. *rabiei* effector (Ar93; unpublished data), possessing a typical secretory signal peptide was transformed into Δ*arf*-*bar* mutant and WT *A*. *rabiei* strains. The effector was constitutively expressed in WT and Δ*arf*-*bar* mutant strains with a c-terminal epitope to detect chimeric effector protein in the culture filtrate. The secreted proteins from *A*. *rabiei* cells were concentrated from culture filtrate. The total cell lysate of fungi was used as expression control along with untransformed strains as negative controls. We were unable to detect secreted Ar93 effector in the culture filtrate of Δ*arf*-*bar* mutant in various biological replicates. However, in wild type the Ar93 effector was secreted (Fig 5D). These results point towards the inability of Δ*arf*-*bar* mutant to secrete Ar93 or similar effectors. Therefore, impaired secretion of effectors or other secreted components must be resulting in Δ*arf*-*bar* mutant’s slow growth and inability to cope with the chickpea basal defense as compared to WT. The entire new experiment has been incorporated in revised manuscript.

**Major Comment 2:** The tubulation micrograph of Fig. S3B is much better than the one used on the main Fig 1D-G. Please can you provide better images for Fig 1? It will be also good to include zoomed areas such as in Fig. S3B to show the difference between the mutants and the wild type, as current images are not very clear. Also, please can this be quantified to denote the relevance? Tubular vs. Non-tubular

**Response 2:** In the revised manuscript, we have changed Fig 1D-G images as per your suggestion so as to provide a better observation of liposome tubulation. Also, we have provided the quantitative data on tubulation in the updated Fig 1H, when liposomes are incubated with buffer control, bacterial purified ArF-BAR protein and its mutant versions. The data clearly shows significant differences in tubulation.

**Major Comment 3:** Authors have not found differences in liposome tubulation when W428 and L430 residues were mutated, however what happens if the entire PKC domain is deleted? Maybe those exact residues are not purely relevant, but the study of the entire domain could provide more substantial information. What will happen to the pathogenicity, the localization?

**Response 3:** We regret for the mistakenly marking C1 domain as PKC domain in Fig 1A. Now in updated Fig 1A, it has been corrected to C1 domain, which is ~ 50 amino acids domain showing similarity to the C1 domain of PKC1. The mutations of W428 and L430 (in *ArF*-*BAR*^mut2^) were created as per previous observation that they are important in C1 domain of hPKC1 (Heung et al. 2004; 279: 21144-21153). Besides the conserved zinc-finger forming cysteine and histidine residues in C1 domain of ArF-BAR, the amino acids W428 and L430 plays a key role in maintaining the DAG-binding groove and the surrounding hydrophobic wall. Thus, they were mutated accordingly. We have checked the localization of ArF-BAR^mut2^ and as compared to WT and it shows no significant difference in localization (Fig 3A). The complementation of Δ*arf*-*bar* mutant with *ArF*-*BAR*^mut2^ construct has defect in virulence (revealed by decreased lesions and their size in Fig. 2C and 2D) as compared to the complementation of Δ*arf*-*bar* mutant with WT *ArF*-*BAR* construct. Therefore, we believe that mutated C1 domain residues play some role in fungal virulence. The wrong marking of PKC domain in ArF-BAR, instead of C1 domain only, created doubts which we regret.

**Major Comment 4:** Further explanation on how the list of putative transcription factors regulating ArF-BAR should be included. It is very good that a list of candidate transcription factors was identified. However, it is necessary to explain better what was used as query to identify this list? Was CRZ1 just selected based on the relationship with stress responses or was there any other evidence? Were other TFs tested? More elaboration on this part is necessary as it is such as important finding that it is hard to understand from the manuscript how CRZ1 was identified. Elaborate materials and methods section too.

**Response:** We agree with your observation on these points and have tried to explain all the mentioned points in the revised manuscript text under results, discussion and methods section.

**Major Comment 5:** Show localization of ArF-BAR, Rab5 and Rab7 in Δ*crz1* mutant. If CRZ1 mutant operates upstream of ArF-BAR, they should be mis-localized.

**Response:** In our study, we have observed that *ArCRZ1* regulates *ArF*-*BAR* at transcriptional level and not at protein-protein interaction level. The ArCRZ1 mediated transcription regulation of *ArF*-*BAR* is proved by protein-DNA interaction and checking expression of *ArF*-*BAR* transcripts in Δ*crz1* mutant. The *ArCRZ1* knock-out mutant has reduced or no expression of *ArF*-*BAR* (Fig. 9B). Hence, ArF-BAR localization was not checked in Δ*crz1* mutant. Moreover, ArCRZ1 regulates other genes too in filamentous fungi so the mis-localization of ArF-BAR, Rab5 and Rab7 (if found in suggested experiments) could not be directly assigned to the ArCRZ1 and ArF-BAR signaling. Although FM 4-64 uptake assay in WT and Δ*arf*-*bar* mutant shows that ArF-BAR regulates endocytosis (Fig 6A).

**Major Comment 6:** In the model, authors suggest that upon ROS perception, intracellular Ca^2+^ increases to activate CRZ1. Authors show that CRZ1 localizes to nuclei during infection (Fig. S12B). I feel that Fig. S12B in which the nuclear localization of CRZ1 is shown during invasive growth should be in the main figure. It is an important and fits with the model. I think it is necessary to show better imaging and include a DIC panel. Also, over a time course, is CRZ1 translocated once the fungus has penetrated the host tissue penetrates?

**Response:** As per your suggestion, we have shifted Fig S12B (of the original manuscript) to the main article figure (Fig. 8E). The figures have been revised and incorporated in revised manuscript (Fig 8E). After conidia germination on chickpea host, we have seen that *ArCRZ1* localizes very early into nucleus of hyphal cells, possibly due to various unknown stresses on host tissue (Fig. 8E) while during normal *in*-*vitro* growth of *A*. *rabiei* it primarily localizes in cytoplasm and moves to nucleus after calcium treatment (Fig 8D).

**Minor point 1:** L115- reference 27 doesn’t correspond to the reference of the genome.

**Response:** The mistake has now been corrected in the revised manuscript. The *A*. *rabiei* genome sequence article referred is Verma et al., 2016 Scientific Reports. The reference is at position [25] in the revised manuscript.

**Minor point 2:** L171- instead of infection bioassay, it will be better to specify that it is a spray inoculation.

**Response:** The sentence has been corrected as per your suggestion and now it mentions ‘conidial suspension spray inoculation’.

**Minor point 3:** L225-ArF-BAR::EYFP was transiently expressed? Could authors state how, which promoter? According to Fig. S5 is native promoter. This needs clarification.

**Response:** The sentence was inadvertently written wrong and we have revised and corrected it in the updated manuscript. The ArF-BAR::EYFP’s stable ectopic expression was achieved by *Agrobacterium* *tumefaciens*-mediated transformation (ATMT), where T-DNA cassette of chimeric construct under *ArF*-*BAR*’s native promoter was integrated into the genome of Δ*arf*-*bar* mutant strain.

**Minor point 4:** Fig1B- was this done in mycelium treatment with menadione or spores? State it in the legend of the figure. Also, please could authors state what was the standard used for the qPCR?

**Response:** Thank you for pointing this mistake in the legend of Fig 1B. The menadione treatment was performed after growing conidia for 7 days. The legend has been appropriately corrected in the revised manuscript. The *A*. *rabiei* elongation factor alpha (*EF1*α) was used as an internal standard for normalization in qRT-PCR. The same was mentioned in original manuscript under ‘RNA extraction and expression analysis’ subheading of materials and methods.

**Minor point 5:** Fig S10B- Line scan legend very small. Please increase font.

**Response:** We have improved this figure by increasing the font size of axis units and symbols. The fig S10B of original manuscript has now been shifted to main results (Fig 6C) of the revised manuscript.

**Minor point 6:** Y2H experiments need to be explained better, at least in the legend or in materials and methods. Please state what are the controls too.

**Response:** The Y2H experiments were performed using split-ubiquitin based Dualhunter system and modified vectors that have been previously explained in detail in our published article (Purayannur et al., 2017; Scientific Reports 7:5026). However, to make our manuscript more understandable, we have explained Y2H experiments in more detail in revised manuscript.

**Minor point 7:** State what is A.U. in the figure legends.

**Response:** The A.U. was fluorescence intensity unit that has been updated in revised figures (Fig 6A-C).

**Minor point 8:** L129- “Consistent with previous observations”

**Response:** By mistake we have not included the reference of previous article (Singh et al., 2012; PLoS ONE 7(3): e33128) where the clone of *ArF*-*BAR* was originally isolated and checked for *in*-*planta* transcript expression after *A*. *rabiei* conidial suspension inoculation on a susceptible chickpea variety Pusa 362. The reference has now been included at this position in the revised manuscript.

**Minor point 9:** Legend panel D fig 1 is missing.

**Response:** In Fig. 1D the liposomes were not supplemented with any protein and only protein resuspension buffer was added. We have modified the legend of Fig 1D for clarity.

**Minor point 10:** Was fluorescence intensity calculated per area? e.g. Figure 4.

**Response:** In Fig. 6A (Fig 4B of the original manuscript) the fluorescence intensity was calculated per area. However, in Fig. 6B and 6C (Fig 4B and S10B of the original manuscript), we forgot to add the line through which the fluorescence intensity was measured in merged image. We have made changes accordingly in the revised manuscript.

**Minor point 11:** Explain legend and controls Fig. 5

**Response:** The legend of Fig 7A and 7B (Fig 5 of original manuscript) has been revised to make better explain the controls and experiment of split-ubiquitin Y2H assay.

**Minor point 12:** Fig. S4. Southern blot, explain better the output of the sizes.

**Response:** Thanks for pointing this issue. We have explained the output sizes in the Southern blot of Δ*arf*-*bar* and Δ*arcrz1* mutant strains in supplementary data of the revised manuscript.
