# Supplementary material for: Modulation of fungal virulence through CRZ1 regulated F-BAR-dependent actin remodeling and endocytosis in chickpea infecting phytopathogen Ascochyta rabiei
Source: PLoS Genet. 2021 May 17;17(5):e1009137. doi: 10.1371/journal.pgen.1009137 (PMC8158962; doi:10.1371/journal.pgen.1009137)
Supplement: Attachment — Submitted filename: Authors Response to Reviewers.doc [file pgen.1009137.s023.doc]

**Authors Response to Editors’ comments**

**Comments:**

L. 28: “..secretion of a candidate effector” (please add “a” to sentence).

L. 65: “for motor-dependent retrograde signaling, endosomes are involved…” (replace “they” with “endosomes”)

L.82: End of line typo (“t)

L. 88: “…virulence of the filamentous fungus…”

**Authors response:** We have incorporated these corrections in the revised manuscript.

**Comment**: L. 119: What is meant with “unigenes”. Please clarify what this term defines.

**Authors response:** The statement made in this line describe about one of our previous study (Singh et al., 2012) where we identified 70 expressed sequence tags (ESTs) that were upregulated in *Ascochyta* *rabiei* under oxidative stress. The ESTs were redundant in cDNA library so a non-redundant set of ESTs (unigenes) was created. The term unigene is commonly used in scientific literature for non-redundant set of sequences derived from redundant sequences of a cDNA library or assembly of sequenced reads (http://www.bioinfo.org.cn/relative/NCBI-UniGene.htm). We have edited this sentence in the revised manuscript to make it comprehensible.

**Comment**: L. 146: It is confusing that the ArF-BAR^mut2^ strain is mentioned here as it is only explained further down what it is. You may either remove the mentioning of this particular mutant here, or you may add “(see below)”.

**Authors response:** As per your and reviewer#2 comments, we have moved ArF-BAR^mut2^ strain to the lower lines of same paragraph, where it has been explained in detail.

**Comments**

L. 170: “..with lipid membranes”

L. 180: “..To assess the effect…”

L. 260: Please remove “However, “. I believe this should be “The localization pattern…”

L. 325: “hypothesized the existence of…”

L. 326: type of first “.”

L. 433: “we propose that ArF-BAR contributes to endocytosis….”

L. 456: “..may be the possible cause…”

**Authors response:** We have incorporated these corrections in the revised manuscript.

**Authors Response to Reviewer #2**

**General comment:** The authors have upgraded the manuscript and have addressed most of the comments of the reviewers. However, after reviewing this second version of the manuscript, I have still found several points and sentences that need rewriting. This should be addressed before acceptance. Taking everything into consideration, my recommendation is Minor Review. I attach a pdf copy of the manuscript with all my comments and corrections. Besides that, I only have minor comments.

**Authors response:** We extend our sincere thanks for such a detailed analysis of our revised manuscript. The sentences that you have marked for rewriting in pdf file has been edited in this revised manuscript. Your marking in pdf file has helped us a lot in editing and improving this manuscript during both revisions. We have also incorporated comprehensive sentences in PGENETICS-D-20-01505R2 wherever you have raised points to improve quality of this manuscript

**Comment 1:** At some points, I wonder if the number of replicates analyzed by the authors is enough.

**Authors response:** In the study whose data is presented in this manuscript, we have performed at least three biological replicates with each biological replicate having many technical replicates. During chickpea host infection assay under controlled conditions, we have performed three biological replicates with each replicate having at least 20 plants. Considering use of 14 fungal strains and taking at least 20 plants for each fungal strain infection during a biological replicate, we believe it is good number in controlled conditions of a growth chamber (Fig 2, Fig 9). For the developmental and stress-related studies of fungi, we have performed at least three biological replicates (Fig 3, Fig 5, supplementary data). However, fungi experiments can be performed in many replicates therefore technical replicates are more in them. To make it stronger, we have revised the main text under various sections to incorporate information about biological and technical replicates. In Fig 1, Fig 2, Fig 7E, etc too, the red dots are of the means from at least three biological replicates.

**Comment 2:** I still recommend to move the figure showing the phenotype of the null *crz1* strain to the main text.

**Authors response:** As per your suggestion, we have moved the figure of Δ*crz1* phenotype under stress conditions to the main Fig 9 panel.

**Comment 3:** I still recommend to give quantitative data, SD and *p* values in the main text. It is done in some cases but I believe that this should be the general rule.

**Authors response:** We have incorporated the quantitative data along with SD and *p* values wherever they were missing in the main text.

**Authors Response to Reviewer # 3**

**General comments by Reviewer # 3:**

In my view this study is well executed. Experiments and evidence shown meet the conclusions and findings proposed. I have just few minor comments.

**Authors Response:** Thank you for your encouraging words. We have revised the manuscript as per comments from editor and reviewers. Hope you will find its quality enhanced now.

**Comment**: L28- “ArF results in delayed formation of apical septum from the growing hyphal tip, crucial for host penetration and impaired secretion of candidate effector having signal peptide”. Sentence confusing.

**Authors Response:** We have edited this sentence so that it can be now intelligible for the general readers.

**Comment**: Fig 5B- increase size of the panels. They are too small.

**Authors Response:** We agree that the panel size was less as compared to other panels of this figure so we have increased size of Fig 5B panel.

**Comment**: Fig 5A- Quantification cell size in panel 5A inside the plant. Could authors quantify cell1 vs cell 2? How many hyphae move from cell 1 vs cell 2 in WT vs. mutant?

**Authors response:** During the growth of fungal cells on glass slides, the cell to cell demarcation can be checked by using dyes (we have used calcofluor white) as septa is visible (Fig 5B). Thus, we were able to quantify septa formation or fungal cell sizes from the growing tip end on glass slides when conidia germinated under hydrated conditions. However, during *in* *planta* growth experiments we had to stain the fungal strains with WGA-488 (Invitrogen, USA) to visualize fungi and measure the depth of their penetration inside susceptible chickpea host tissue. We have noted that wild-type *A*. *rabiei* strain was able to penetrate the host tissue and proliferate while Δ*arf*-*bar* was unable to penetrate beyond a limit (Fig 5A). However, during *in* *planta* growth it is difficult to mark the individual cells of *Ascochyta* *rabiei* hyphae. Thus, we were unable to quantify the cell size and delayed septa formation during *in* *planta* growth. Hope you will acknowledge these technical difficulties.

**Comment**: L303 EEs state first that are Early Endosomes.

**Authors response:** The term early endosomes (EEs) have been mentioned with full- and short-forms under the introduction section.

**Comments**:

L292 This should be in lower case after coma.

L326-327 “interactions of ArF-BAR with ArActin.g Y2H system, it was shown that ArF-BAR does not interact directly with actin (Fig 7A),” This sentence is confusing

L382- to silence- change to inhibit

**Authors response:** We have incorporated required corrections and revised the text of these sentences so that they become clearer to readers.

**Comment**: L357-361 How was the length of the ArF-BAR promoter chosen on the first place? Was it arbitrary to take 1kb? It will be useful to show all the putative binding sites of each transcription factor. This is a beautiful analysis that needs to be better explained and shown.

**Authors response:** The *ArF*-*BAR* (ST47_g8005) and ST47_g8004 genes are placed in opposite orientation in the assembled genome of *A*. *rabiei*. Hence, they have a common 5′ upstream region of 727 bp between their translational start site (ATG). In the revised supplementary Table 1, we have highlighted the position of various fungal transcription factors core-binding sites in 5’ upstream region of *ArF*-*BAR* gene. A cartoon representation has also been presented in the revised supplementary Table 1. The core binding site of CRZ1 has also been shown in main text of revised manuscript along with their positions at three places. We hope the readers would be able to understand it in a much better way.
